# Dual-Alpha: a large EEG study for dual-frequency SSVEP brain–computer interface

**DOI:** 10.1093/gigascience/giae041

**Published:** 2024-08-07

**Authors:** Yike Sun, Liyan Liang, Yuhan Li, Xiaogang Chen, Xiaorong Gao

**Affiliations:** The School of Biomedical Engineering, Tsinghua University, Beijing 100084, China; The China Academy of Information and Communications Technology, Beijing 100191, China; Institute of Biomedical Engineering, Chinese Academy of Medical Sciences and Peking Union Medical College, Tianjin 300192, China; The School of Life Sciences, Tiangong University, Tianjin 300387, China; Institute of Biomedical Engineering, Chinese Academy of Medical Sciences and Peking Union Medical College, Tianjin 300192, China; The School of Biomedical Engineering, Tsinghua University, Beijing 100084, China

**Keywords:** brain–computer interface, dual-frequency, SSVEP, EEG, dataset

## Abstract

**Background:**

The domain of brain–computer interface (BCI) technology has experienced significant expansion in recent years. However, the field continues to face a pivotal challenge due to the dearth of high-quality datasets. This lack of robust datasets serves as a bottleneck, constraining the progression of algorithmic innovations and, by extension, the maturation of the BCI field.

**Findings:**

This study details the acquisition and compilation of electroencephalogram data across 3 distinct dual-frequency steady-state visual evoked potential (SSVEP) paradigms, encompassing over 100 participants. Each experimental condition featured 40 individual targets with 5 repetitions per target, culminating in a comprehensive dataset consisting of 21,000 trials of dual-frequency SSVEP recordings. We performed an exhaustive validation of the dataset through signal-to-noise ratio analyses and task-related component analysis, thereby substantiating its reliability and effectiveness for classification tasks.

**Conclusions:**

The extensive dataset presented is set to be a catalyst for the accelerated development of BCI technologies. Its significance extends beyond the BCI sphere and holds considerable promise for propelling research in psychology and neuroscience. The dataset is particularly invaluable for discerning the complex dynamics of binocular visual resource distribution.

## Graphical Abstract

**Graphical Abstract:**
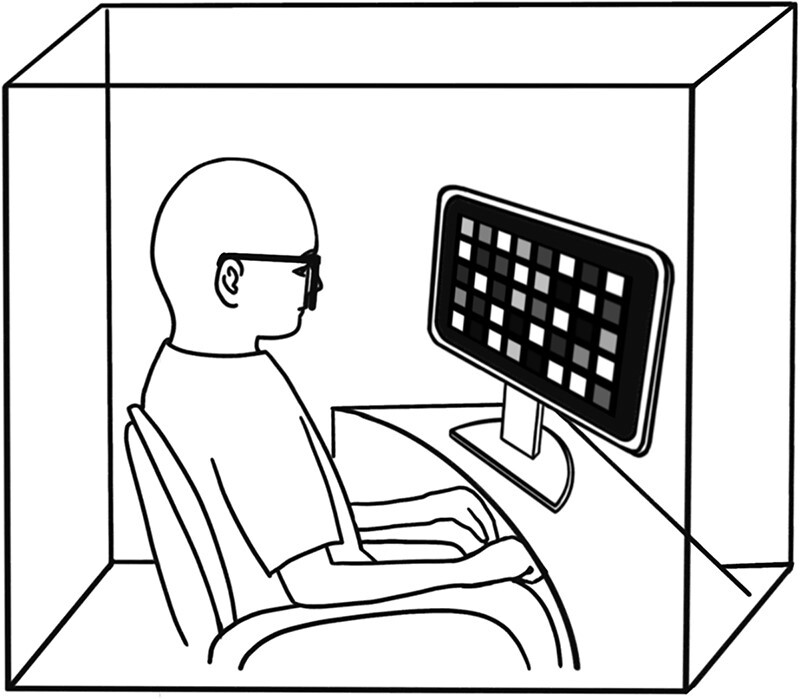

## Data Description

Brain–computer interface (BCI) research is currently one of the most vibrant fields of study [1, 2]. Among various BCI technologies, electroencephalogram (EEG)–based interfaces are deemed particularly suitable for consumer electronics applications in sectors like education due to their noninvasive nature and ease of use [3, 4]. Within this domain, steady-state visual evoked potential (SSVEP)–based BCIs have emerged as some of the most accurate and stable systems available [5, 6].

SSVEPs are frequency-locked and phase-locked brain activities predominantly occurring in the occipital region when an individual observes a flickering light stimulus at a fixed frequency [7]. These signals are extensively utilized in BCI research for functions such as typing and device control. Given that SSVEP responses are typically confined to specific frequency bands [8, 9], dual-frequency SSVEP studies have become a focal point, aiming to enhance the capacity of SSVEP systems to handle more extensive target selections [10]. The exploration of dual-frequency SSVEP represents one of the most promising areas of current research.

Recent years have seen the proposal of various dual-frequency stimulation techniques by researchers, encompassing methods like the checkerboard arrangement (CA) paradigm [10] and the left-right visual field paradigm, among others. A notable advancement is the enhanced CA introduced in 2020 [11]. However, a persistent challenge across these paradigms is the generation of unpredictable intermodulation harmonic components (UIHCs) in the form $a*f1\,\, + \,\,b*f2$, where *a* and *b* are arbitrary integers [10–13]. Attempts to harness these intermodulation frequencies for coding have largely been unsuccessful due to their instability and individual variability [12, 13].

In response, a study in 2022 introduced a dual-frequency SSVEP paradigm named binocular vision (BV) using 3-dimensional (3D) display technology, leveraging polarized light to effectively separate the dual frequencies and reduce UIHC generation [14]. Furthering this approach, the 2024 introduction of the binocular-swap vision (BsV) paradigm utilizes a similar stimulation strategy but incorporates a specialized coding and decoding algorithm to efficiently utilize the differential visual capacities of the 2 eyes, making it one of the most effective dual-frequency SSVEP BCI systems to date [15]. Both the BV and BsV paradigms employ identical stimulus and data acquisition methods; however, they differ significantly in their coding schemes. The BV paradigm continues to rely on traditional frequency identification for decoding, whereas the BsV paradigm, facing the presence of targets with identical frequencies, places greater emphasis on the differences in the spatial distribution of dominant eye effects for decoding purposes. Consequently, the BsV paradigm exhibits enhanced potential for coding and decoding within dual-frequency paradigms.

The progression of algorithmic research in BCIs is increasingly leaning toward data-driven approaches, underscoring the critical need for high-quality datasets [16]. There is a plethora of SSVEP datasets covering diverse aspects, including real-world usage scenarios [17], motion-based datasets [18], and multifrequency SSVEP datasets [19], along with mixed-paradigm datasets [20].

However, high-quality datasets specifically crafted for the prevalent 40-target SSVEP input keyboards are notably scarce. This is particularly critical given that one of the primary applications of SSVEP technology is currently the development of these 40-target keyboards [21, 22]. Despite this, the field still faces a significant shortage of comprehensive dual-frequency 40-target SSVEP datasets, which are essential for the advancement of BCI technologies. To bridge this gap, we have developed the Dual-Alpha dataset. This dataset is uniquely designed for the 3 most effective dual-band paradigms—CA, BV, and BsV—and is distinguished as the largest and only dual-frequency SSVEP dataset tailored specifically for 40-target applications.

## Methods

### Participant information and experimental setup

Our study included over 100 participants. Detailed demographic information is presented in Table 1. The experiments for the 3 paradigms were conducted independently, with voluntary enrollment, and each subject was numbered in the order of enrollment. For the CA paradigm, 35 individuals participated, with an average age of 23.9 years, comprising 22 males and 13 females. In the BsV paradigm, 35 participants were involved, with a mean age of 23.3 years, including 21 males and 14 females. Similarly, in the BV paradigm, 35 participants were involved, with a mean age of 23.2 years, including 23 males and 12 females. Notably, the majority of participants were unfamiliar with SSVEP-based BCI technologies. None of the participants had any ophthalmic or neurological conditions. Some subjects participated in multiple paradigm experiments; this information is provided in Supplementary Table S1.

**Table 1:** Participant information statistics

Paradigms	Number of subjects	Age, mean ± standard deviation	Gender	Dominant eye
Checkerboard arrangement	35	23.9 ± 3.0	22 M 13F	
Binocular-swap vision	35	23.3 ± 1.2	21 M 14F	Left: 9 Right: 26
Binocular Vision	35	23.2 ± 1.8	23 M 12F	Left: 8 Right: 27

**Figure 1:**
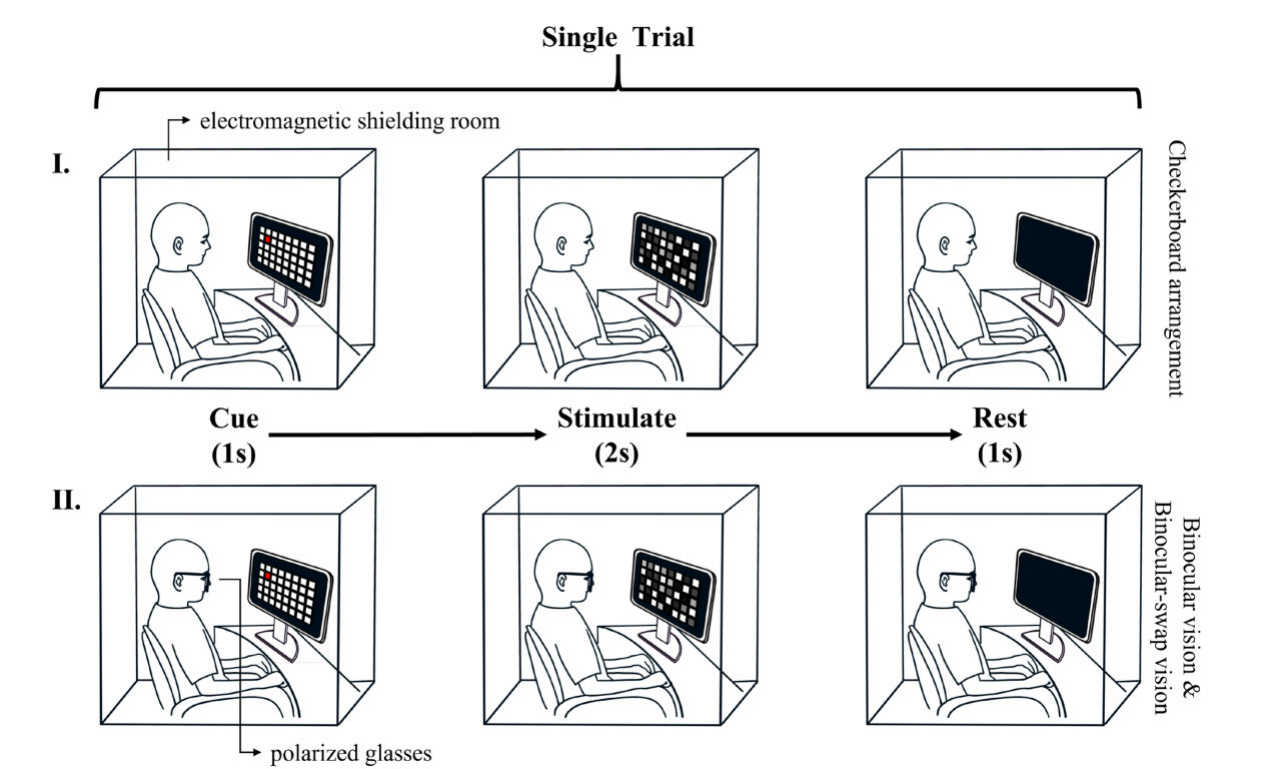
Schematic representation of the single-trial flow of the experiment, divided into 3 phases: cue, stimulate, and rest, lasting 1 second, 2 seconds, and 1 second, respectively. Panel I outlines the experimental flow for the CA paradigm. Conversely, Panel II provides a common schematic for both the BsV and BV paradigms.

As illustrated in Fig. 1, each participant was seated in a dark, electromagnetically shielded room, maintaining a fixed distance of 80 cm from the stimulus screen. The trial commenced with a 1-second cue period, during which the target for the next stimulus was highlighted in red, allowing the participant to focus. This was followed by a 2-second stimulation period, wherein the participants concentrated solely on the previously cued target. A subsequent 1-second rest period was observed, during which participants were advised to remain still and avoid any movements or blinking. The stimulus and signal acquisition methods for CV is shown in Fig. 1(I). And The stimulus and signal acquisition methods for both the BV and BsV paradigms were identical, thus the diagrams of these two paradigms are presented in Fig. 1(II). Participants wore polarizing glasses throughout the experiments. For subjects who wore glasses, clip-on polarized glasses were used, and for those who did not wear glasses, frame polarized glasses were provided. Each participant underwent a total of 200 trials, with each of the 40 targets being presented in 5 distinct trials. The sequence of stimulus targets was randomized by the computer system to prevent anticipatory biases.

### Stimulation systems

The experimental setup incorporated a stimulation host running on a Windows operating system (NVIDIA GeForce RTX 3080, Intel Core i7-10700 CPU 2.90 GHz) and utilized a 27-inch stimulation screen (Model D2769Vh, 1920 × 1080 resolution, $60\,\,Hz$). This screen supports a polarized light 3D display. The stimulus presentation software was developed using MATLAB 2021a (RRID:SCR_001622) in conjunction with the Psychophysics Toolbox version 3 (RRID:SCR_002881) [23].

**Figure 2:**
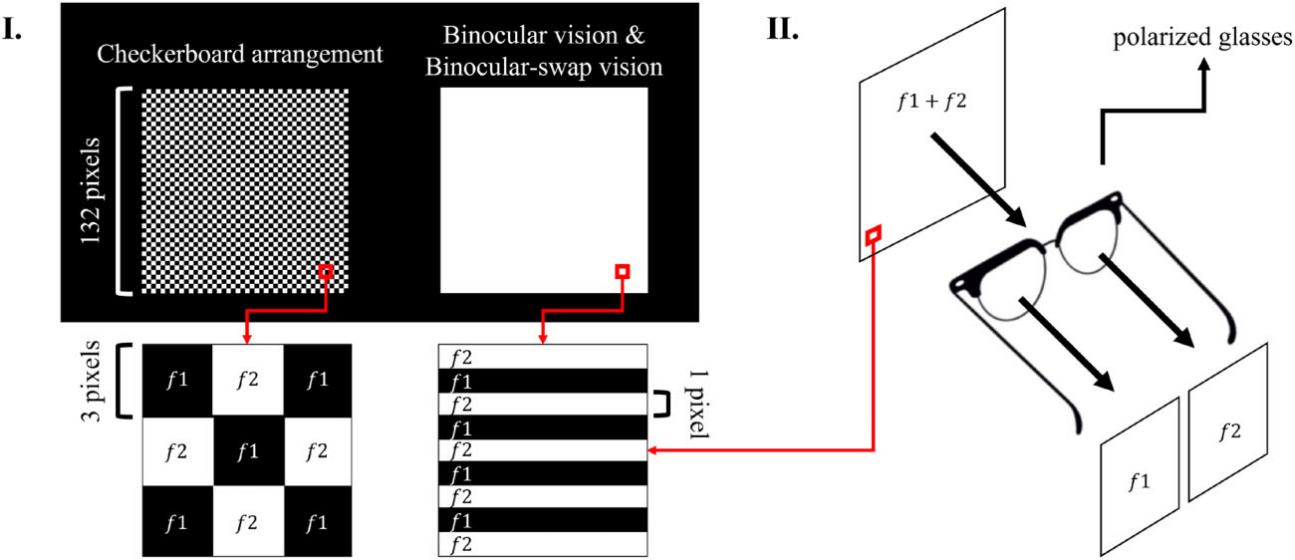
Schematic representation of the single-target composition of the dual-frequency SSVEP. Panel I on the left illustrates the single-target composition of the CA paradigm, with a partially zoomed-in schematic showing the alternating frequencies resembling a chessboard grid. Panel I on the right depicts the single-target composition of the BV and BsV paradigms, with a partially zoomed-in view where the difference between the 2 stimulus frequencies is not directly detectable. Panel II details the demodulation process of the stimulus targets for the BV and BsV paradigms, where the fused frequency combinations $f1$ and $f2$ in the human eye are demodulated by polarized light and displayed to the subject’s left and right eyes, respectively.

The configuration of the stimulus targets is depicted in Fig. 2. The luminance sequences for all the targets were designed based on the joint frequency-phase modulation (JFPM) technique [24]. In the dual-frequency stimulus configuration, involving frequencies $f1$ and $f2$, the luminance sequences are mathematically expressed as:


(1) \begin{eqnarray*}
S\left( {f,\sigma ,\varphi } \right) = int\left\{ {255{\mathrm{*}}\left[ {0.5 + 0.5{\mathrm{*}}\cos \left( {\frac{{2\pi *f*\sigma }}{r} + \varphi } \right)} \right]} \right\}
\end{eqnarray*}

where *S* denotes the luminance sequence of each frame, with values representing the gray levels on the display ranging from 0 to 255. The variable $\sigma $ represents the number of frames, with the display refresh rate being $60\,\,Hz$, and hence $\sigma $ varies from 1 to 60 multiplied by the stimulation duration. $\varphi $ denotes the phase, and *f* represents the stimulation frequency. *r* indicates the display refresh rate.

**Figure 3:**
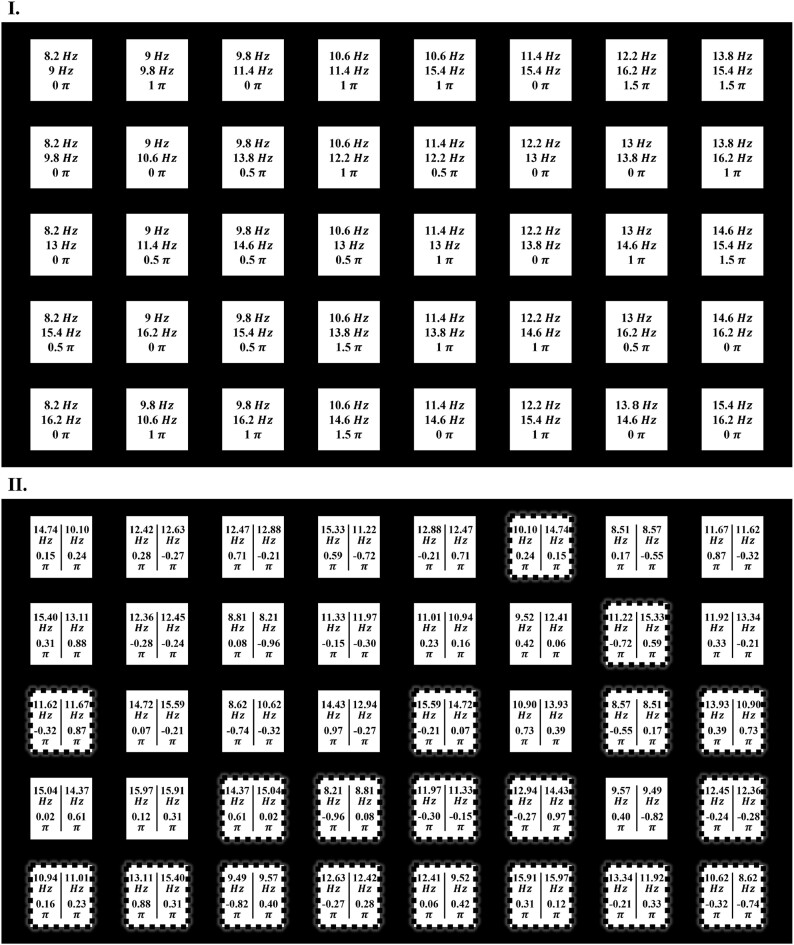
Schematic representation of the stimulus interface with encoding details. Panel I illustrates the frequency-phase encoding scheme used for the CA and BV paradigms, featuring a total of 40 targets organized into 5 rows and 8 columns. Panel II displays the encoding scheme for the BsV paradigm. Here, the stimuli on the left side correspond to those assigned to the left eye, and those on the right to the right eye. The stimuli within dashed boxes indicate the target groups postfrequency swap between the eyes, whereas those within solid boxes represent the original target groups.

Regarding the spatial configuration of the stimulus targets, 3 paradigms are addressed in this study. For the CA paradigm, the stimulus target is illustrated on the left side of Fig. 2(I) and is structured similarly to a chessboard grid. For the 2 frequencies $f1$ and $f2$, they are alternated among the stimulus targets, with each small grid measuring 3 pixels, totaling a stimulus target size of 132 × 132 pixels. To human perception, the stimulus target appears as a combination of 2 distinct frequencies. The stimulus targets were arranged at equal intervals across the screen in the form of 5 rows and 8 columns, and the luminance of the interval portion was always 0. The specific arrangement can be seen in Fig. 3.

The BV paradigm and the BsV paradigm constructions are presented on the right side of Fig. 2(I). To the human eye, the stimulus appears as a summation of $f1$ and $f2$ frequencies. However, upon closer inspection, the stimuli are interlaced, with only 1 pixel per line, making the spatial differences imperceptible to the human eye. The demodulation processes for BV and BsV are illustrated in Fig. 2(II), where the vibrational phases of the polarized light emitted by $f1$ and $f2$ stimuli differ. These can be remodulated through the demodulation of polarizing glasses to $f1$ and $f2$, with $f1$ presented to the left eye and $f2$ to the right eye. It is worth noting that the polarized light technique causes each eye to see only half of the pixels on the stimulus screen emitting light, the other half being filtered out by the polarizer due to a phase mismatch. Thus, the intensity of light for stimuli in the BV and BsV paradigms is actually only half that of the CV paradigm.

### Stimulus interface and encoding

Figure 3 illustrates the stimulus configuration and encoding methodologies employed in this research. The coding of CV and BV paradigms adopts the optimal coding scheme from the study by Liang et al. in 2020 [11]. The BsV paradigm utilizes the coding scheme derived using the global optimization method from the study by Sun et al. in 2022 [14]. Among the 3 paradigms examined, the CV and BV paradigms adhere to traditional frequency-identification coding schemes. These schemes necessitate unique frequency combinations for each target, ensuring that the responses evoked by each target differ significantly in the frequency spectrum. The coding scheme utilized in this study represents the optimal combination of frequencies, as proposed in prior research. The specific arrangement of frequencies and phases, along with their values, is detailed in Fig. 3(I).

Conversely, the BsV paradigm leverages the spatial differences arising from the prevalent dominant eye effect in the population to facilitate classification. Within this paradigm, some stimulus targets share identical frequency combinations, resulting in nearly indistinguishable evoked response spectra. However, the assignment of frequencies to the left and right eyes differs (e.g., for stimulus target A: left eye $f1$, right eye $f2$; for stimulus target swap-A: left eye $f2$, right eye $f1$), which creates distinct spatial patterns in the responses that are used for classification tasks. The encoding scheme for the BsV is depicted in Fig. 3(II), where targets enclosed in solid borders represent the top 20-group encoding scheme derived from global optimization in previous studies. Targets within dashed borders indicate new target groups resulting from a swap of stimulus frequencies between the left and right eyes.

### Data acquisition and processing

For the data acquisition in this study, a NEUROSCAN EEG amplifier (RRID:SCR_015818) and a 64-lead Neuroscan Quik-Cap EEG Cap (RRID:SCR_015817) were employed, adhering to the international 10–20 system for electrode placement. In the case of the CA and BV paradigms, which primarily involve the occipital region, only the 9 electrodes located in this area were utilized, specifically Pz, PO5, PO3, POz, PO4, PO6, O1, Oz, and O2. For the BsV paradigm, owing to the broader distribution of significant interclass differences across the brain regions [15], data from all 64 electrodes were collected.

The acquired experimental data underwent a downsampling process to decrease the sampling rate from 1,000 Hz to 250 Hz. This was followed by the application of comb filters to eliminate direct current signals and reduce intermediate frequency interference, utilizing the MNE toolbox (RRID:SCR_005972) [25, 26]. The data preprocessing was carried out from raw data using the EEGLAB (RRID:SCR_007292) toolkits, known for their computational efficiency [27].

The assessment of the signal-to-noise ratio (SNR) was performed to better evaluate the performance of the dual-band paradigm. The calculations for wideband SNR, narrowband SNR, and intermodulation SNR [14] were conducted as per the following formulas:


(2) \begin{eqnarray*}
\left\{ {\begin{array}{@{}*{1}{c}@{}} {{\mathrm{SN}}{{\mathrm{R}}_{{\mathrm{Broadband}}}} = \frac{{\mathop \sum \nolimits_{\delta = 1}^h N\left( {\delta {f_1}} \right) + N\left( {\delta {f_2}} \right)}}{{\mathop \sum \nolimits_{f = 5Hz}^{100Hz} N\left( f \right) - \mathop \sum \nolimits_{\delta = 1}^5 N\left( {\delta {f_1}} \right) + N\left( {\delta {f_2}} \right)}}}\\ {{\mathrm{SN}}{{\mathrm{R}}_{{\mathrm{Narrowband\,\,}}}} = \frac{{\mathop \sum \nolimits_{\delta = 1}^h \left[ {N\left( {\delta {f_1}} \right) + N\left( {\delta {f_2}} \right)} \right]}}{{\mathop \sum \nolimits_{k = - {f_b}/\Delta f}^{{f_b}/\Delta f} \mathop \sum \nolimits_{\delta = 1}^h \left[ {N\left( {\delta {f_1} + k\Delta f} \right) + N\left( {\delta + k\Delta f} \right)} \right] - \mathop \sum \nolimits_{\delta = 1}^5 [N\left( {\delta {f_1}} \right) + N\left( {\delta {f_2}} \right)]}}}\\ {{\mathrm{SN}}{{\mathrm{R}}_{{\mathrm{Intermodulation}}}} = \frac{{\mathop \sum \nolimits_{\delta = 1}^h [N\left( {\delta {f_1}} \right) + N\left( {\delta {f_2}} \right)]}}{{\mathop \sum \nolimits_{a = - h}^h \mathop \sum \nolimits_{b = - h}^h N\left( {a{f_1} + b{f_2}} \right) - \mathop \sum \nolimits_{\delta = 1}^5 \left[ {N\left( {\delta {f_1}} \right) + N\left( {\delta {f_2}} \right)} \right]}}} \end{array}} \right. \end{eqnarray*}

In the given study, the variables ${\mathrm{SN}}{{\mathrm{R}}_{{\mathrm{Broadband}}}}$, ${\mathrm{SN}}{{\mathrm{R}}_{{\mathrm{Narrowband\,\,}}}}$, and ${\mathrm{SN}}{{\mathrm{R}}_{{\mathrm{Intermodulation}}}}$ denote the values of the wideband SNR, narrowband SNR, and intermodulation SNR, respectively. The terms $f1$ and $f2$ correspond to the combination of stimulus frequencies utilized in the dual-band configuration. The function *N* indicates the energy associated with these frequency points. The symbol *h* signifies the number of harmonics considered, which, for this research, is set at 5. The parameter ${f_b}$, defined as 2 in this study, represents the bandwidth utilized for the narrowband evaluations. $\Delta f$ is the reciprocal of the sampling time length of the signal, which is $1/2$ in this study.

The wideband SNR quantifies the ratio of the energies of $f1$ and $f2$, along with their harmonics, relative to the entire frequency spectrum, thus reflecting the strength of the SSVEP signal. The narrowband SNR, pivotal for SSVEP classification accuracy, is calculated as the ratio of the energy of $f1$ and $f2$, including their harmonics, to the energy within a $4\,\,Hz$ bandwidth centered on these frequencies.

Furthermore, the intermodulation SNR, which is crucial for assessing the strength of the UIHC specific to dual-band stimuli, is measured as the ratio of the energies of $f1$ and $f2$, and their harmonics, to the energy at the frequency band where UIHC ($af1 + bf2$, where *a, b* range from −5 to 5) is observed. It is noteworthy that higher values of intermodulation SNR correspond to weaker representations of the UIHC, which implicates its diminished influence in the presence of strong intermodulation components.

### SSVEP classification algorithm

To evaluate the quality of the dataset further, classification analysis was performed using established algorithms within the domain. The SSVEP classification algorithms fall into 2 primary categories: nontraining and training-based methods [28]. However, due to the limited adaptation of many algorithms to the dual-frequency paradigm, we selected 1 representative algorithm from each category for our analysis.

For the nontraining category, we utilized the filter bank dual-frequency canonical correlation analysis (FBDCCA) [14]. This method is an adaptation of the classical filter bank canonical correlation analysis (FBCCA) [29], specifically modified to handle dual-frequency SSVEP systems. The FBDCCA algorithm enhances the detection of dual-frequency targets by modifying the templates of FBCCA to accommodate dual frequencies. In our study, we constructed templates using sine–cosine matrices derived from the first to fifth harmonics of the 2 frequencies associated with the stimulus targets. These templates were then processed through a filter bank composed of 5 bandpass filters, with ranges set at [5, 95 Hz], [12, 95 Hz], [19, 95 Hz], [27, 95 Hz], and [35, 95 Hz]. Subsequently, a canonical correlation analysis (CCA) was performed, and the outputs were linearly weighted to generate the final correlation sequence. The template displaying the highest correlation was identified as the predicted result.

In the training-based category, we employed the task-related component analysis (TRCA) algorithm [30]. TRCA enhances classification performance by using training data to compute a null-domain filter, thus optimizing the detection of task-related components. In this research, the validation was conducted using the leave-one-out method, and the aggregate results were expressed as mean values. The filter banks were configured with the following frequency ranges: [5, 95 Hz], [12, 95 Hz], [19, 95 Hz], and [27, 95 Hz]. Additionally, an ensemble strategy was implemented for the computations.

Additionally, due to the constraints in time length for plotting traditional spectra, we opted to use CCA spectra instead. This approach utilizes the correlation values calculated by the CCA algorithm [31], denoted as ρ, plotted against frequency, providing a spectrum-like representation but with higher resolution [32]. The method first constructs the desired sine–cosine template and subsequently performs a CCA operation with the corresponding EEG data time series to obtain correlation values. Although this method does not provide phase information, its frequency resolution is higher. We consider that this method sacrifices phase information to enhance frequency resolution. The template $Temple( {f,\,\,t} )$ can be represented by the following equation:


(3) \begin{eqnarray*}
\textit{Temple}\left( {f,\,\,t} \right) = \left[ {\begin{array}{@{}*{1}{c}@{}} {{\mathrm{cos}}\left( {2\pi ft} \right)}\\ {{\mathrm{sin}}\left( {2\pi ft} \right)} \end{array}} \right]
\end{eqnarray*}

The computation of this spectrum is described by the following equation:


(4) \begin{eqnarray*}
\rho \left( f \right) = CCA\left[ {x\left( t \right),\textit{Temple}\left( {f,\,\,t} \right)} \right]
\end{eqnarray*}

Here, $\rho $ represents the value on the vertical axis of the CCA spectrum, and *f* denotes the frequency, ranging from $5$ to $35\,\,Hz$ with increments of $0.1\,\,Hz$ in our analysis. The variable *t* represents the time-series data, and $x( t )$ represents EEG data time series.

In addition, we use the ITR metric in measuring the classification accuracy of the SSVEP system, which is calculated as in Equation 4, where *T* is the length of the selected time window (in seconds), and an additional 0.5 seconds will be used as the target search time to simulate the real situation. *n* is the number of stimulus targets. *p* is the classification accuracy, with a value between 0 and 1.


(5) \begin{eqnarray*}
ITR = \frac{{60}}{{T + 0.5}}\left\{ {{{\log }_2}n + P{{\log }_2}P + \left( {1 - P} \right){{\log }_2}\left[ {\frac{{1 - P}}{{n - 1}}} \right]} \right\}
\end{eqnarray*}

## Data Validation and Quality Control

### Frequency domain analysis validation

**Figure 4:**
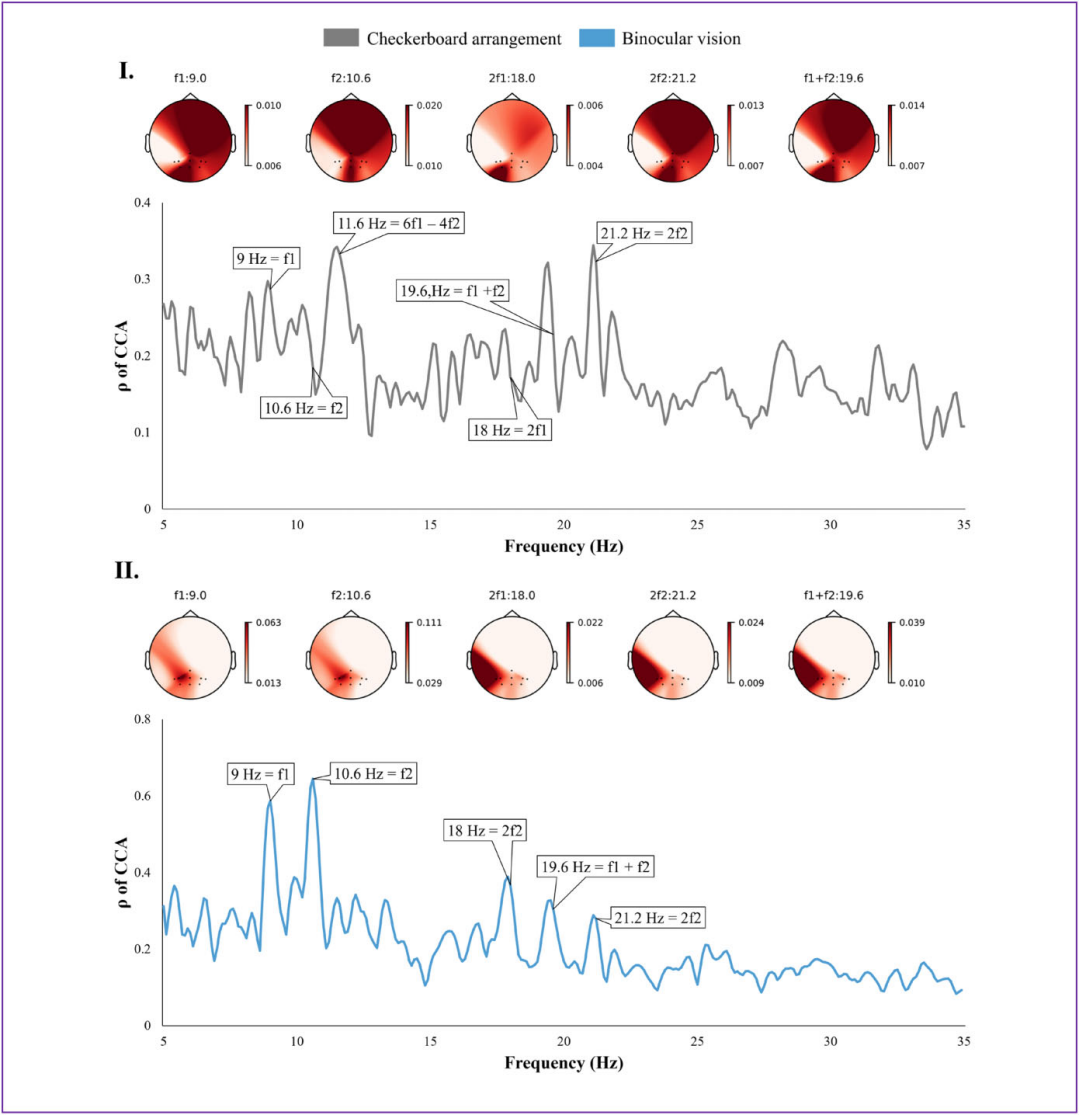
CCA spectra and normalized PSD topography for the CA and BV paradigms at frequencies $f1$ of $9.0\,\,Hz$ and $f2$ of $10.6\,\,Hz$. Panel I: Gray lines denote the results from the CA analysis, sourced from CA paradigm group subject 01. Panel II: Blue lines denote the results from the BV paradigm analysis, sourced from BV paradigm group subject 01.

To ascertain the integrity of the dataset, we initially engaged in the analysis of time-domain signals and distributions, presenting representative results in Fig. 4. As depicted in Fig. 4(I), the UIHC in the CA paradigm exhibits significant strength, and there is considerable variability both within and between subjects regarding the evoked frequencies. For instance, the subject illustrated in Fig. 4(I) demonstrated a UIHC at a frequency of $11.6\,\,Hz$, calculated as $6*f1 - 4*f2$. Conversely, the primary frequencies of the CA paradigm, specifically components $f1$ and $f2$, displayed instability; for example, the $10.6\,\,Hz$ stimulus in Fig. 4(I) was nearly imperceptible, yet its second harmonic at $21.2\,\,Hz$ was pronounced.

The UIHC in the BV paradigm was comparatively less prevalent, and its main frequency component appeared more stable, as evidenced in Fig. 4(II). This stability can be attributed to the application of polarized light technology, which effectively prevents the overlap of the 2 stimulus frequencies before reaching the retina. Nonetheless, the BV paradigm did not eliminate the occurrence of UIHC, as demonstrated by the presence of a $19.6\,\,Hz$ frequency ($f1 + f2$) in Fig. 4(II). These findings align with previous research [14], underscoring the high quality of the dataset.

**Figure 5:**
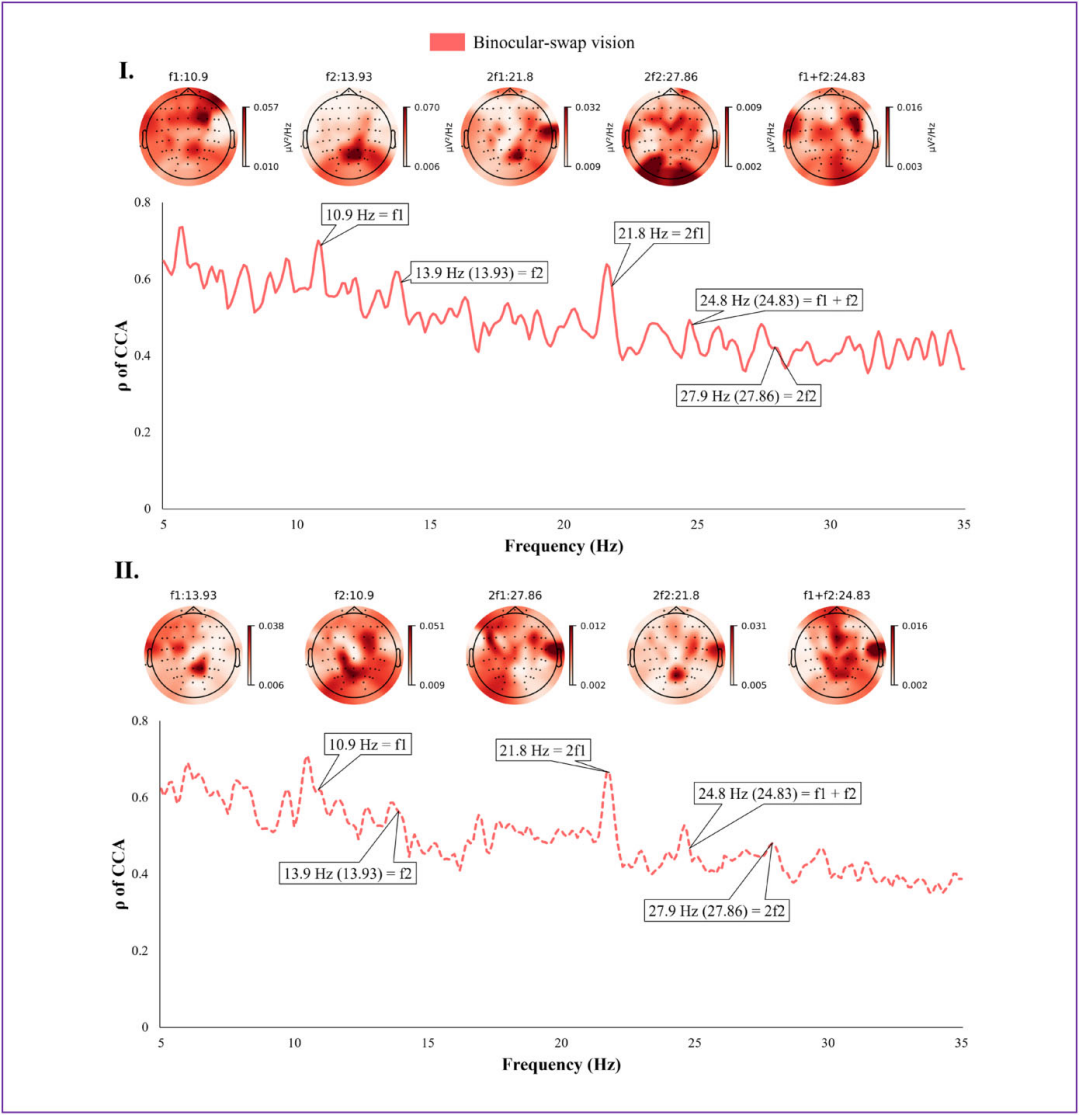
CCA spectra of the BsV paradigm with normalized PSD topography. Panel I: Solid lines represent results for a stimulus target with the left eye frequency $f1$ of $13.93\,\,Hz$ and the right eye frequency $f2$ of $10.9\,\,Hz$. Panel II: Dashed lines represent results for stimulus targets with the left eye frequency $f1$ of $10.9\,\,Hz$ and the right eye frequency $f2$ of $13.93\,\,Hz$. All data sourced from BsV paradigm group subject 01.

For the BsV paradigm, evaluations were conducted independently due to its distinct encoding approach and the acquisition of a more extensive array of leads. Figure 5 illustrates typical frequency domain and topographic map schematics; Fig. 5(I) displays the left eye stimulus analysis results at frequency $f1$ of $10.9\,\,Hz$ and the right eye at frequency $f2$ of $13.93\,\,Hz$, while Fig. 5(II) presents the inverse. These results highlight that the frequency characteristics evoked by these stimulus targets are remarkably similar and nearly identical. However, there is a notable difference in their PSD topography, attributed to the disparate allocation of visual resources between the 2 eyes. This differential resource distribution underscores the efficacy of the BsV paradigm in performing classifications.

### SNR ratio distribution analysis

**Figure 6:**
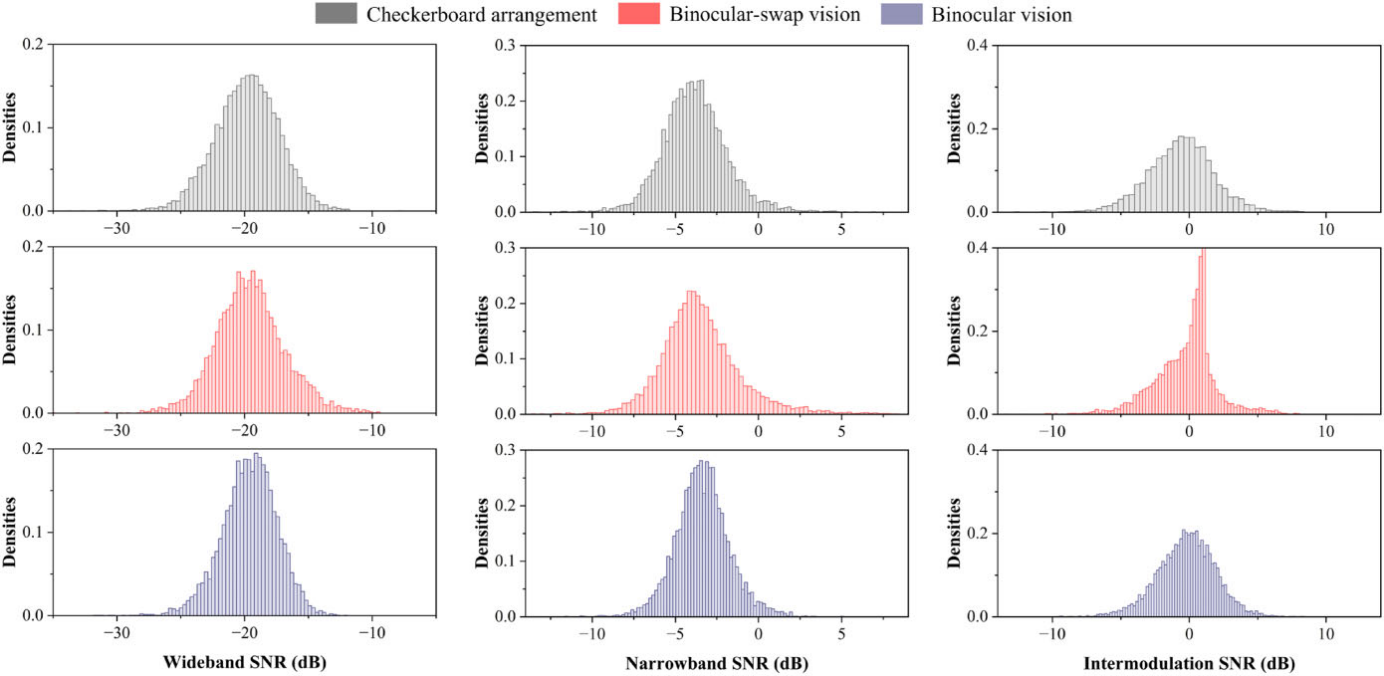
Signal-to-noise ratio distribution for a single trial: gray represents the CA paradigm, red denotes the BsV paradigm, and blue indicates the BV paradigm. The first column shows the wideband SNR distribution, the second column the narrowband SNR distribution, and the third column the intermodulation SNR distribution.

To assess the overall quality of the dataset, we computed the wideband SNR, narrowband SNR, and intermodulation SNR for a single trial across each of the 3 paradigms, with the results depicted in Fig. 6. When compared to datasets such as Beta [33], our SNR distributions are all normal but overall more skewed. This skewness correlates with the presence of UIHC in the dual-frequency paradigm, among other factors. Compared to the same multifrequency dataset study [19], our SNR distributions are very similar. These findings confirm the stability and quality of our dataset. Notably, the distribution of the BsV paradigm in the intermodulation signal-to-noise ratio exhibited a significant shift. This shift is thought to be associated with the distribution of the dominant eye among the subject population, predominantly right-eyed, as detailed in Table 1. This factor likely influenced the generation of the UIHC, underscoring the dataset’s considerable potential for psychological and neurobiological research. Noting that although the BsV paradigm was acquired for 64 leads at the time of acquisition, only data from the 9 leads of the occipital region were used in the calculation of SNR as in the other 2 paradigms.

### Average SNR

**Figure 7:**
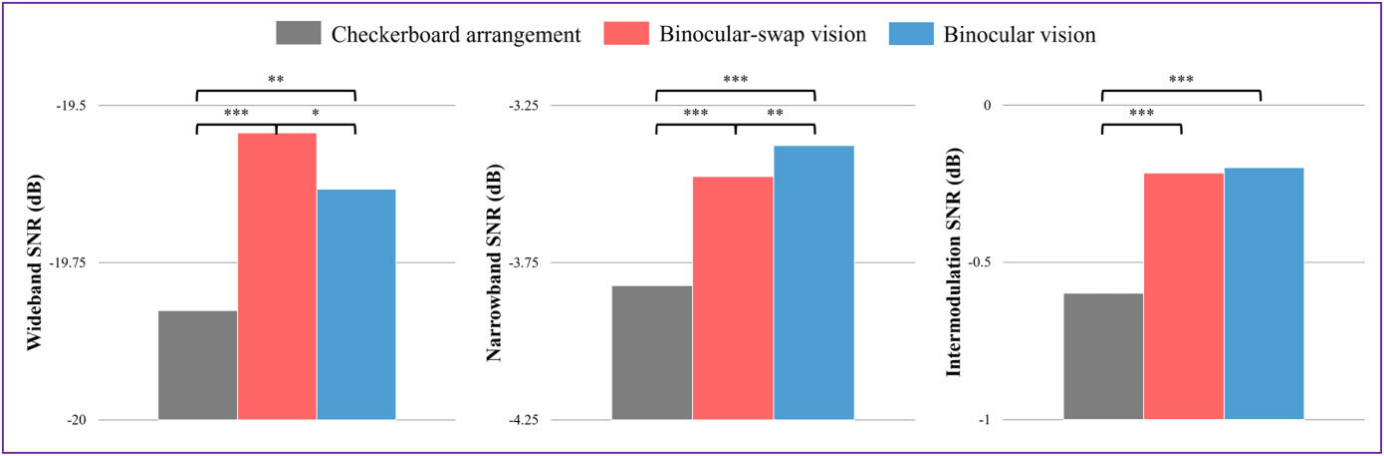
Bar chart of the mean values of wideband SNR, narrowband SNR, and intermodulation SNR: gray corresponds to the CA paradigm, red to the BsV paradigm, and blue to the BV paradigm. The asterisks denote the results of Welch’s independent *t*-tests for significant differences.

Further analysis involved calculating the average SNR, with findings presented in Fig. 7. The BsV paradigm exhibited relatively high values for both wideband and narrowband SNR, followed by the BV paradigm, while the CA paradigm recorded the lowest values, likely due to the instability of the dominant frequency in this paradigm. In terms of intermodulation SNR, both the BV and BsV paradigms outperformed the CA paradigm, suggesting a lower generation of UIHC in these paradigms. These results align with previous research, affirming the dataset’s quality [14]. However, it is important to note that both the wideband and narrowband SNR of the current dataset are lower than those reported in single-frequency SSVEP datasets, potentially due to the diversion of UIHC for total stimulus response energy.

### Classification results without training

**Figure 8:**
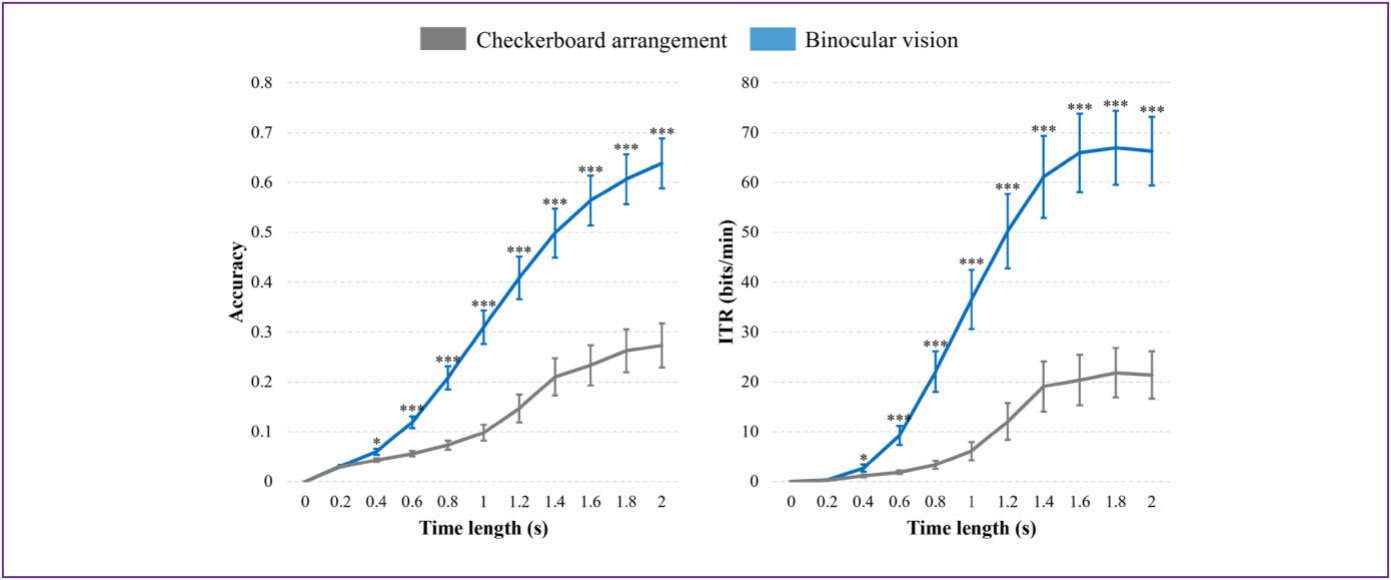
The plot of untrained classification results over time, where blue represents the BV paradigm and gray represents the CA paradigm. The left graph illustrates the correctness curve and the right graph displays the ITR curve. Error bars indicate standard errors. The asterisks denote the results of Welch’s independent *t*-tests for significant differences.

Given that the SSVEP paradigm predominantly serves classification tasks, we analyzed the dataset accordingly. For the no-training scenario, we implemented the FBDCCA method. Due to the inherent characteristics of the BsV paradigm, which encodes the same for 2 sets of targets, it precludes the feasibility of no-training classification. Therefore, our analysis was confined to the CA and BV paradigms, with the findings depicted in Fig. 8. It is evident from the figure that the BV paradigm, benefiting from a stable principal frequency, retains some utility even without training. In contrast, the CV paradigm proves virtually inapplicable without training due to significant individual variability in the UIHC. Specific categorization results can be found in Supplementary Table S2.

### Classification results with training

**Figure 9:**
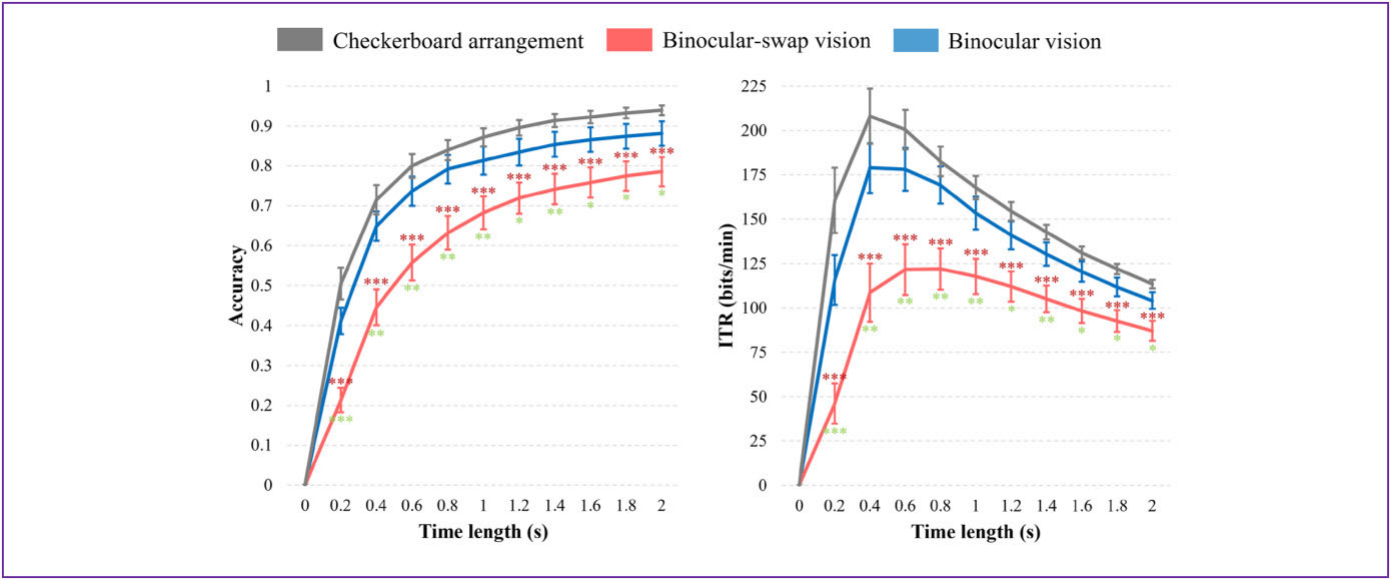
The plot of trained classification results over time, where blue indicates the BV paradigm, red indicates the BsV paradigm, and gray indicates the CA paradigm. The left plot shows the correctness curve and the right plot shows the ITR curve. Error bars are standard errors. The green asterisks are the results of Welch’s independent *t*-tests for significant differences between the BV paradigm and the BsV paradigm, and the dark red asterisks are the results of Welch’s independent *t*-tests for significant differences between the CA paradigm and the BsV paradigm. There was no significant difference between the results of the CA and BV paradigms.

Subsequently, we conducted an algorithmic analysis incorporating training, employing the TRCA algorithm within the SSVEP framework. This computation was executed using the leave-one-out approach, utilizing 4 trials for training and 1 for testing at each instance. The average outcomes and the results of statistical tests are illustrated in Fig. 9. The results indicate that the performance metrics of correctness and ITR for both the CA and BV paradigms are closely matched, with no significant difference observed. The CA paradigm slightly outperformed, possibly due to the polarized light technique used in both the BV and BsV paradigms, which reduces light intensity by half. Despite the BsV’s close frequency resemblance and its focus primarily on the null domain, it does not match the efficacy of TRCA algorithms. The BsV paradigm is trained and tested with 64-lead data. While there are specialized algorithms enhancing performance in the null domain [15], they do not apply to the other paradigms and thus are not discussed in this article. Specific categorization results can be found in Supplementary Table S3.

### Effect of the number of channels on classification

**Figure 10:**
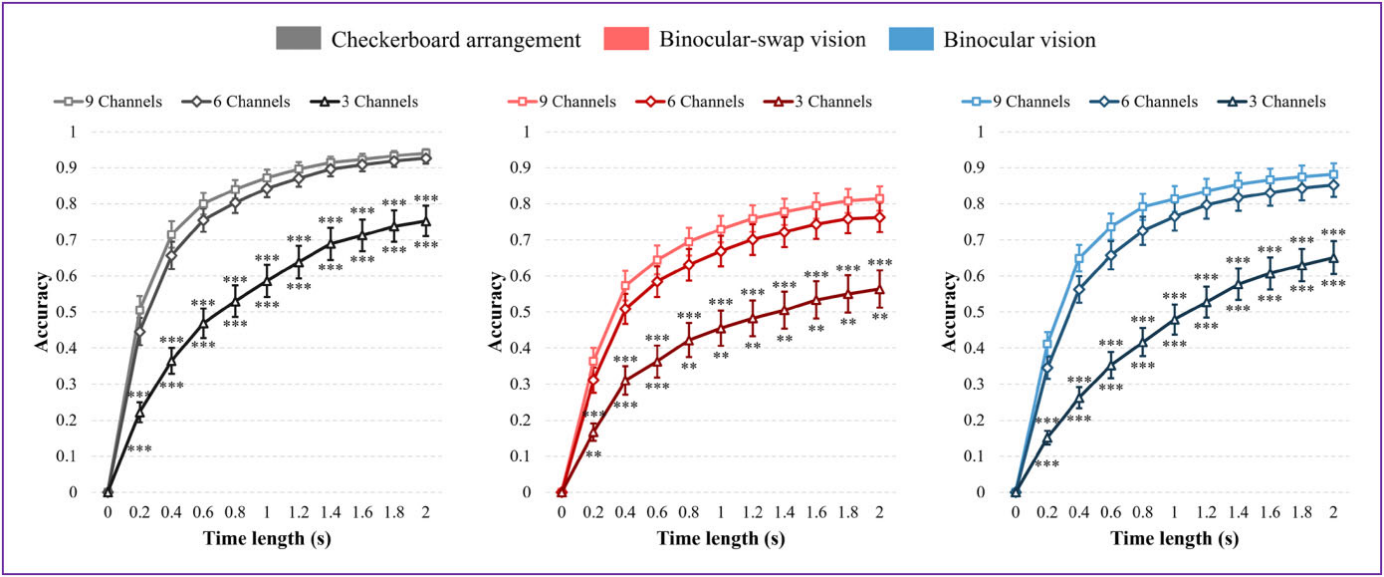
Classification accuracy of the 3 paradigms under the TRCA algorithm for different channel configurations. The left graph represents the CA paradigm, the middle graph represents the BsV paradigm, and the right graph represents the BV paradigm. The channel configurations are 3-channel, 6-channel, and 9-channel. Results marked with an asterisk indicate significance as determined by Welch’s independent *t*-test. The asterisks on the top side indicate the results of the statistical test between the 3-channel results and the 9-channel results, and the asterisks on the bottom side indicate the results of the statistical test between the 3-channel results and the 6-channel results. There is no significant difference between the 6-channel results and the 9-channel results.

Lead selection is a critical factor in BCI studies focusing on SSVEP [34]. This study employed a 9-channel acquisition system targeting the occipital region, based on findings from a previous single-frequency study. To determine whether the 9-channel configuration provides nonredundant information across the 3 paradigms investigated, classification tasks were conducted sequentially using 3-channel (O1, Oz, and O2), 6-channel (PO3, POz, PO4, O1, Oz, and O2), and 9-channel (Pz, PO5, PO3, POz, PO4, PO6, O1, Oz, and O2) setups, utilizing the TRCA algorithm. The results, depicted in Fig. 10, indicate that the classification accuracy for all 3 paradigms improves with an increasing number of channels.

## Conclusion and Discussion

In this study, we introduced the Dual-Alpha dataset, the largest and only dual-frequency SSVEP dataset specifically tailored for 40-target applications. Our comprehensive dataset, encompassing over 100 participants, underwent rigorous validation through SNR analyses and classification. The validation results highlighted the dataset’s high quality and stability.

Despite these strengths, our dataset has certain limitations. One significant issue is the synchronous nature of the data, which restricts its applicability to specific types of BCI systems. The current dataset lacks asynchronous system data, which is crucial for developing more flexible and practical BCI applications [35–37]. Asynchronous systems allow for more natural and spontaneous interactions, better mimicking real-life scenarios where users can switch tasks and modes of operation without predefined time constraints.

Future research should focus on expanding the dataset to include asynchronous system data. This would enable the development of more sophisticated algorithms capable of handling the dynamic and unpredictable nature of real-world BCI applications. Additionally, investigating methods to further reduce the presence of UIHC and enhance the robustness of frequency-locked responses across different paradigms will be essential for advancing research of dual-frequency SSVEP BCI.

## Usage Notes

The dataset is organized into 3 folders, each corresponding to a different paradigm:

*Binocular Vision/*

*Binocular-Swap Vision/*

*Checkerboard Arrangement/*

Each folder contains data files for individual subjects, named in the format “SUBJECT X.csv.” The structure of these CSV files is as follows:

*First Column: Number*

*Second Column: Timestamp*

*Third Column: Condition (corresponds to the stimulus code in “Stimulate Code.txt”)*

*Fourth Column: Epoch number*

*Subsequent* Columns*: EEG data, with the first row indicating the name of each lead*

Each CSV file contains EEG data from 5 blocks, covering 40 targets, resulting in a total of 200 trials per subject. The data are sampled at 250 Hz, with timestamps ranging from 0.14 to 2.14 seconds after stimulus onset. These CSV files can be easily read using the Pandas package in Python (RRID:SCR_018214).

Additionally, each subject has a corresponding information file named “SUBJECT X.txt.”

## Supplementary Material

giae041_GIGA-D-24-00125_Original_Submission

giae041_GIGA-D-24-00125_Revision_1

giae041_Response_to_Reviewer_Comments_Original_Submission

giae041_Reviewer_1_Report_Original_Submission Yichuan Jiang -- 5/13/2024 Reviewed

giae041_Reviewer_2_Report_Original_Submission Marc Van Hulle -- 5/15/2024 Reviewed

giae041_Reviewer_3_Report_Original_Submission Jing Mu -- 5/16/2024 Reviewed

giae041_Supplemental_File

## Data Availability

The datasets supporting the results of this article are available in the *GigaScience* repository, GigaDB [38].

## Additional Files

**Supplementary Table S1**. Duplicate participants’ information statistics.

**Supplementary Table S2**. Classification results of FBDCCA.

**Supplementary Table S3**. Classification results of TRCA.

## Abbreviations

BCI: brain–computer interface; BV: binocular vision; BsV: binocular-swap vision; CA: checkerboard arrangement; CCA: canonical correlation analysis; EEG: electroencephalogram; FBCCA: filter bank canonical correlation analysis; FBDCCA: filter bank dual-frequency canonical correlation analysis; ITR: information transfer rate; SSVEP: steady-state visually evoked potential; TRCA: task-related component analysis; UIHC: unpredictable intermodulation harmonic components
