# Supplementary material for: Dual-Alpha: a large EEG study for dual-frequency SSVEP brain–computer interface
Source: Gigascience. 2024 Aug 7;13:giae041. doi: 10.1093/gigascience/giae041 (PMC11304967; doi:10.1093/gigascience/giae041)

GigaScience

Dual-Alpha: A Large EEG Study for Dual-Frequency SSVEP Brain-Computer Interface

--Manuscript Draft--

**Manuscript Number:**	GIGA-D-24-00125
**Full Title:**	Dual-Alpha: A Large EEG Study for Dual-Frequency SSVEP Brain-Computer Interface
**Article Type:**	Data Note
**Funding Information:**	National Natural Science Foundation of China (U2241208, 62171473) — Dr Yike Sun<br>National Key Research and Development Program of China (2023YFF1205300, 2022YFC3602803) — Dr Yike Sun<br>Key Research and Development Program of Ningxia (2023BEG02063) — Dr Yike Sun
**Abstract:**	Background: The domain of brain-computer interface (BCI) technology has experienced significant expansion in recent years. However, the field continues to face a pivotal challenge due to the dearth of high-quality datasets. This lack of robust datasets serves as a bottleneck, constraining the progression of algorithmic innovations and, by extension, the maturation of the BCI field.<br>Findings: This study details the acquisition and compilation of electroencephalogram (EEG) data across three distinct dual-frequency steady-state visual evoked potential (SSVEP) paradigms, encompassing over one hundred participants. Each experimental condition featured 40 individual targets with 5 repetitions per target, culminating in a comprehensive dataset consisting of 21,000 trials of dual-frequency SSVEP recordings. We performed an exhaustive validation of the dataset through signal-to-noise ratio (SNR) analyses and Task-related Component Analysis (TRCA), thereby substantiating its reliability and effectiveness for classification tasks.<br>Conclusions: The extensive dataset presented is set to be a catalyst for the accelerated development of BCI technologies. Its significance extends beyond the BCI sphere and holds considerable promise for propelling research in psychology and neuroscience. The dataset is particularly invaluable for discerning the complex dynamics of binocular visual resource distribution.
**Corresponding Author:**	Yike Sun<br>Tsinghua University<br>Beijing, CHINA
**Corresponding Author Secondary Information:**	
**Corresponding Author's Institution:**	Tsinghua University
**Corresponding Author's Secondary Institution:**	
**First Author:**	Yike Sun
**First Author Secondary Information:**	
**Order of Authors:**	Yike Sun
	Liyan Liang
	Yuhan Li
	Xiaogang Chen
	Xiaorong Gao
**Order of Authors Secondary Information:**	
**Additional Information:**	
**Question**	**Response**

Powered by Editorial Manager® and ProduXion Manager® from Aries Systems Corporation

Are you submitting this manuscript to a special series or article collection?	No
**Experimental design and statistics**<br><br>Full details of the experimental design and statistical methods used should be given in the Methods section, as detailed in our Minimum Standards Reporting Checklist. Information essential to interpreting the data presented should be made available in the figure legends.<br><br>Have you included all the information requested in your manuscript?	Yes
**Resources**<br><br>A description of all resources used, including antibodies, cell lines, animals and software tools, with enough information to allow them to be uniquely identified, should be included in the Methods section. Authors are strongly encouraged to cite Research Resource Identifiers (RRIDs) for antibodies, model organisms and tools, where possible.<br><br>Have you included the information requested as detailed in our Minimum Standards Reporting Checklist?	Yes
**Availability of data and materials**<br><br>All datasets and code on which the conclusions of the paper rely must be either included in your submission or deposited in publicly available repositories (where available and ethically appropriate), referencing such data using a unique identifier in the references and in the "Availability of Data and Materials" section of your manuscript.<br><br>Have you have met the above requirement as detailed in our Minimum	Yes

Standards Reporting Checklist?	

Powered by Editorial Manager® and ProduXion Manager® from Aries Systems Corporation

Manuscript Click here to access/download;Manuscript;Dual Alpha.docx

Dual-Alpha: A Large EEG Study for Dual-Frequency SSVEP Brain-Computer Interface

Yike Sun[1, +], Liyan Liang[2, +], Yuhan Li[3, 4, +], Xiaogang Chen[3, *] and Xiaorong Gao[1, *]

Yike Sun sun.yk.bci@outlook.com; Liyan Liang: 18618488256@163.com; Yuhan Li: 13463949502@163.com; Xiaogang Chen: chenxg@bme.cams.cn; Xiaorong Gao: gxr-dea@mail.tsinghua.edu.cn

[1]the School of Biomedical Engineering, Tsinghua University, Beijing, 100084, China.

[2]the China Academy of Information and Communications Technology, Beijing, 100191, China.

[3]Institute of Biomedical Engineering, Chinese Academy of Medical Sciences and Peking Union Medical College, Tianjin, 300192, China.

[4]the School of Life Sciences, Tiangong University, Tianjin, 300387, China.

[*]Correspondence address. Institute of Biomedical Engineering, Chinese Academy of Medical Sciences and Peking Union Medical College, Tianjin, 300192, China. Email: chenxg@bme.cams.cn, and the School of Biomedical Engineering, Tsinghua University, Beijing, 100084, China. Email: gxr-dea@mail.tsinghua.edu.cn.

[+]These authors contributed equally to this work.

Abstract

Background: The domain of brain-computer interface (BCI) technology has experienced significant expansion in recent years. However, the field continues to face a pivotal challenge due to the dearth of high-quality datasets. This lack of robust datasets serves as a bottleneck, constraining the progression of algorithmic innovations and, by extension, the maturation of the BCI field.

Findings: This study details the acquisition and compilation of electroencephalogram (EEG) data across three distinct dual-frequency steady-state visual evoked potential (SSVEP) paradigms, encompassing over one hundred participants. Each experimental condition featured 40 individual targets with 5 repetitions per target, culminating in a comprehensive dataset consisting of 21,000 trials of dual-frequency SSVEP recordings. We performed an exhaustive validation of the dataset through signal-to-noise ratio (SNR) analyses and Task-related Component Analysis (TRCA), thereby substantiating its reliability and effectiveness for classification tasks.

Conclusions: The extensive dataset presented is set to be a catalyst for the accelerated development of BCI technologies. Its significance extends beyond the BCI sphere and holds considerable promise for propelling research in psychology and neuroscience. The dataset is particularly invaluable for discerning the complex dynamics of binocular visual resource distribution.

Keywords: Brain-computer interface; Dual-frequency; SSVEP; EEG; Dataset.

Data Description

Context

Brain-computer interface (BCI) research is currently one of the most vibrant fields of study [1]. Among various BCI technologies, EEG-based interfaces are deemed particularly suitable for consumer electronics applications in sectors like education due to their non-invasive nature and ease of use [2]. Within this domain, steady-state visual evoked potential (SSVEP) based BCIs have emerged as some of the most accurate and stable systems available [3].

SSVEP, or steady-state visually evoked potentials, are frequency-locked and phase-locked brain activities predominantly occurring in the occipital region when an individual observes a flickering light stimulus at a fixed frequency [4]. These signals are extensively utilized in BCI research for functions such as typing and device control [3, 5]. Given that SSVEP responses are typically confined to specific frequency bands [6, 7], dual-frequency SSVEP studies have become a focal point, aiming to enhance the capacity of SSVEP systems to handle more extensive target selections [8]. The exploration of dual-frequency SSVEP represents one of the most promising areas of current research.

Recent years have seen the proposal of various dual-frequency stimulation techniques by researchers, encompassing methods like the Checkerboard arrangement (CA) paradigm [8] and the left-right visual field paradigm [9], among others. A notable advancement is the enhanced CA introduced in 2020 [10]. However, a persistent challenge across these paradigms is the generation of unpredictable intermodulation harmonic components (UIHC) in the form $a * f1 + b * f2$, where a and b are arbitrary integers [8-12]. Attempts to harness these intermodulation frequencies for coding have largely been unsuccessful due to their instability and individual variability [11, 12]. In response, a study in 2022 introduced a dual-frequency SSVEP paradigm using 3D display technology, leveraging polarized light to effectively separate the dual frequencies and reduce UIHC generation [13]. Furthering this approach, the 2024 introduction of the Binocular-swap vision (BsV) paradigm utilizes a similar stimulation strategy but incorporates a specialized coding and decoding algorithm to efficiently utilize the differential visual capacities of the two eyes, making it one of the most effective dual-frequency SSVEP BCI systems to date [14].

The progression of algorithmic research in BCIs is increasingly leaning towards data-driven approaches, underscoring the critical need for high-quality datasets [15]. There is a plethora of SSVEP datasets covering diverse aspects, including real-world usage scenarios [16, 17], motion-based datasets [18], high-frequency datasets [19], and multi-frequency SSVEP datasets [20], along with mixed paradigm datasets [21].

However, high-quality datasets specifically designed for the widely used 40-target SSVEP input keyboards are less common. The Benchmark dataset of 2017 [22] and the 2020 Beta dataset [23] are considered gold standards in SSVEP research. The 2022 release of the eld-beta dataset targets older adults [24], yet the field still lacks comprehensive dual-frequency 40-target SSVEP datasets, which are critical for advancing BCI technologies. To address this gap, we have compiled the Dual-Alpha dataset, which is tailored for the three most effective dual-band paradigms—CA, BV, and BsV—and currently holds the distinction of being the largest and only dual-frequency SSVEP dataset specifically designed for 40-target applications.

Methods

Participant information and experimental setup

Our study encompassed over one hundred participants. The detailed demographic information is tabulated in **Table 1**. For the CA paradigm, a total of 35 individuals participated, with an average age of 23.9 years, consisting of 22 males and 13 females. For the BsV paradigm, 35 participants were involved, with a mean age of 23.3 years, including 21 males and 14 females. And in BV paradigm study, there are 35 participants as well, with a mean age of 23.2 years old, including 23 males and 12 females. Notably, the majority of participants across the three paradigms were distinct individuals, and most were not previously familiar with SSVEP-based BCI technologies.

Table 1. Participant information statistics

Paradigms	Number of subjects	Average age	Gender
Checkerboard Arrangement	35	23.9±3.0	22M 13F
Binocular-swap Vision	35	23.3±1.2	21M 14F
Binocular Vision	35	23.2±1.8	23M 12F

As illustrated in **Figure 1**, the experimental procedure was rigorously designed to ensure consistency. Each participant was seated in a dark, electromagnetically shielded room, maintaining a fixed distance of 80 cm from the stimulus screen. The trial commenced with a 1-second cue period, during which the target for the next stimulus was highlighted in red, allowing the participant to focus. This was followed by a 2-second stimulation period, wherein the participants concentrated solely on the previously cued target. A subsequent 1-second rest period was observed, during which participants were advised to remain still and avoid any movements or blinking. As depicted in **Figure 1(II)**, participants in the BV and BsV paradigms wore polarizing glasses throughout the experiment. Each participant underwent a total of 200 trials, with each of the 40 targets being presented in 5 distinct trials. The sequence of stimulus targets was randomized by the computer system to prevent anticipatory biases.

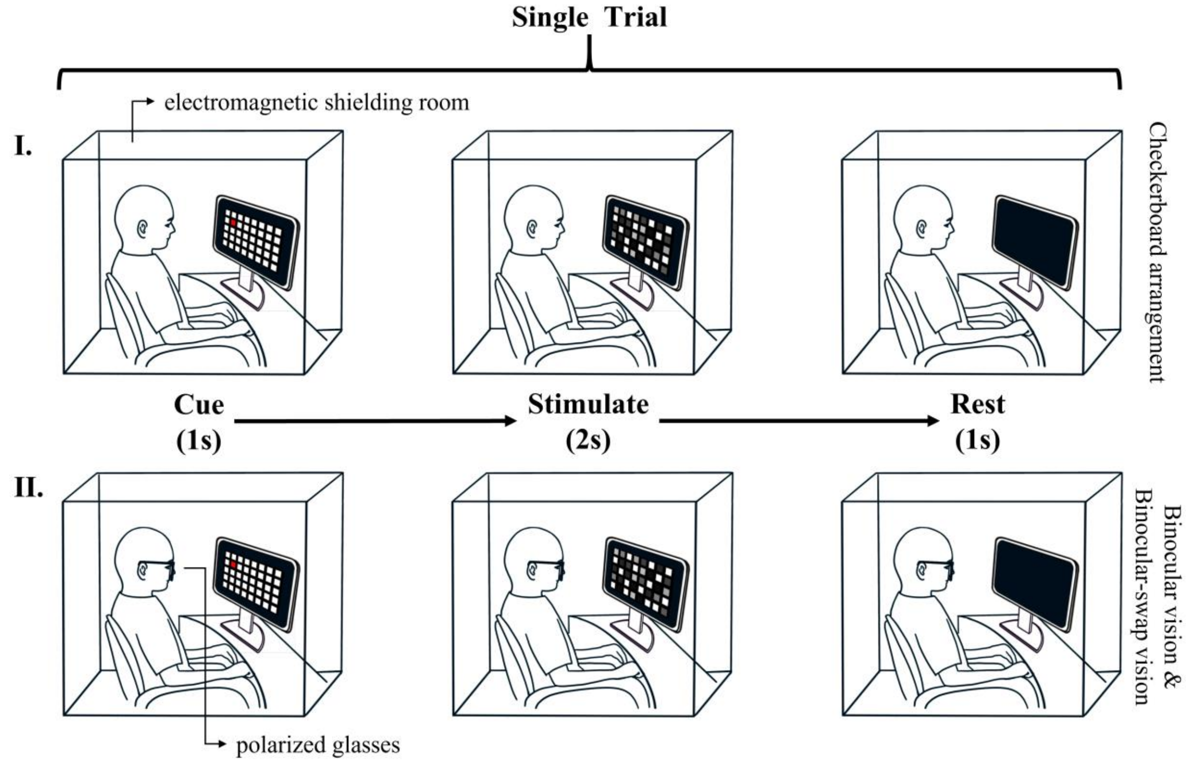

Figure 1. Schematic representation of the single-trial flow of the experiment, divided into three phases: cue, stimulate, and rest, lasting 1s, 2s, and 1s respectively. **Panel I** outlines the experimental flow for the CA paradigm, while **Panel II** details the flow for the BsV and BV paradigms.

Stimulation systems

The experimental setup incorporated a stimulation host running on a Windows operating system (NVIDIA GeForce RTX 3080, Intel(R) Core(TM) i7-10700 CPU 2.90GHZ) and utilized a 27-inch stimulation screen (Model D2769Vh, 1920 × 1080 resolution). This screen supports a polarized light 3D display. The stimulus presentation software was developed using MATLAB 2021a in conjunction with the Psychophysics Toolbox version 3 [25].

The configuration of the stimulus targets is depicted in **Figure 2**. The luminance sequences for all the targets were designed based on the Joint Frequency-Phase Modulation (JFPM) technique [3]. In the dual-frequency stimulus configuration, involving frequencies $f1$ and $f2$, the luminance sequences are mathematically expressed as:

$$\boldsymbol{S}(f,\sigma,\varphi) = int\left\{255 * \left[0.5 + 0.25 * \cos\left(\frac{2\pi * f}{\sigma * r} + \varphi\right)\right]\right\} \quad (1)$$

where $\boldsymbol{S}$ denotes the luminance sequence of each frame, with values representing the grey levels on the display ranging from 0 to 255. The variable σ represents the number of frames, with the display refresh rate being $60\ Hz$, hence σ varies from 1 to 60 multiplied by the stimulation duration. φ denotes the phase, and f represents the stimulation frequency.

Regarding the spatial configuration of the stimulus targets, three paradigms are addressed in this study. For the CA paradigm, the stimulus target is illustrated on the left side of **Figure 2(I)** and is structured similarly to a chessboard grid. For the two frequencies $f1$ and $f2$, they are alternated among the stimulus targets, with each small grid measuring 3 pixels, totaling a stimulus target size of 132 × 132 pixels. To human perception, the stimulus target appears as a combination of two distinct frequencies.

The BV paradigm and the BsV paradigm constructions are presented on the right side of **Figure 2(I)**. To the human eye, the stimulus appears as a summation of $f1$ and $f2$ frequencies. However, upon closer inspection, the stimuli are interlaced, with only one pixel per line, making the spatial differences imperceptible to the human eye. The demodulation processes for BV and BsV are illustrated in **Figure 2(II)**, where the vibrational phases of the polarized light emitted by $f1$ and $f2$ stimuli differ. These can be

re-modulated through the demodulation of polarizing glasses to $f1$ and $f2$, with $f1$ presented to the left eye and $f2$ to the right eye. Notably, this method effectively doubles the light intensity; however, the stimuli in the BV and BsV paradigms are theoretically only half as bright as those in the CA paradigm.

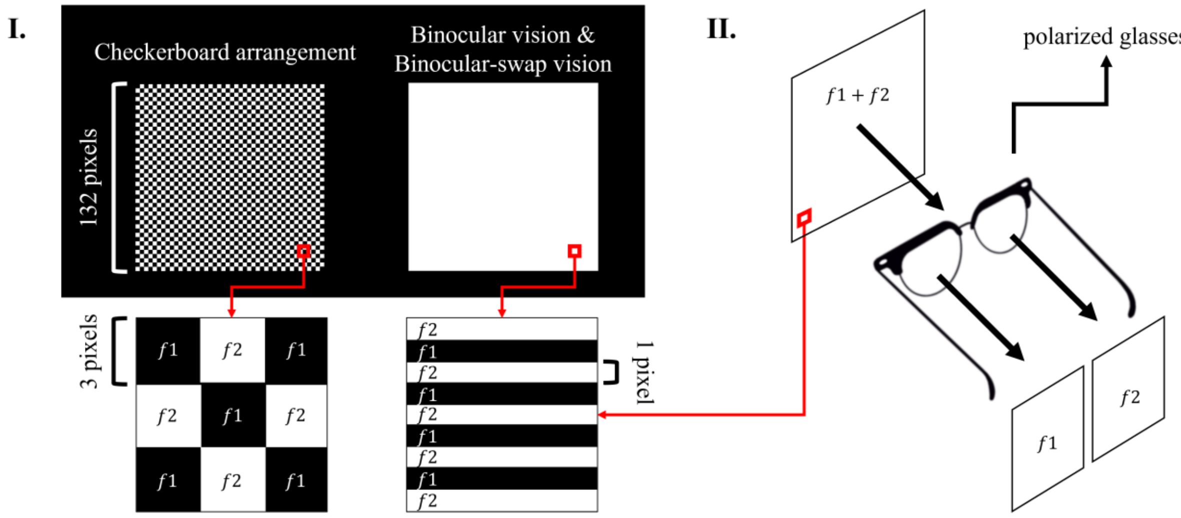

Figure 2. Schematic representation of the single-target composition of the dual-frequency SSVEP. **Panel I.** on the left illustrates the single-target composition of the CA paradigm, with a partially zoomed-in schematic showing the alternating frequencies resembling a chessboard grid. **Panel I.** on the right depicts the single-target composition of the BV and BsV paradigms, with a partially zoomed-in view where the difference between the two stimulus frequencies is not directly detectable. **Panel II**. details the demodulation process of the stimulus targets for the BV and BsV paradigms, where the fused frequency combinations $f1$ and $f2$ in the human eye are demodulated by polarized light and displayed to the subject's left and right eyes, respectively.

Stimulus interface and encoding

The stimulus configuration and encoding methodologies utilized in this research are depicted in **Figure 3**. This study continues to explore the development of an SSVEP-based typing keyboard interface, employing a 40-target arrangement for both the CA paradigm and the BV paradigm, as outlined in **Figure 3(I)**. This figure illustrates the frequency-phase encoding scheme utilized for these paradigms.

Figure 3(II) details the encoding scheme for the BsV paradigm. This paradigm leverages the differential visual processing capabilities of the binocular system to distinguish between targets that share the same frequency but are assigned differently to the left and right eyes. Consequently, only 20 sets of stimuli are required to achieve the encoding objectives. The targets within dashed boxes represent those that have undergone a frequency swap between the left and right eyes, highlighting the novel targets introduced by this manipulation.

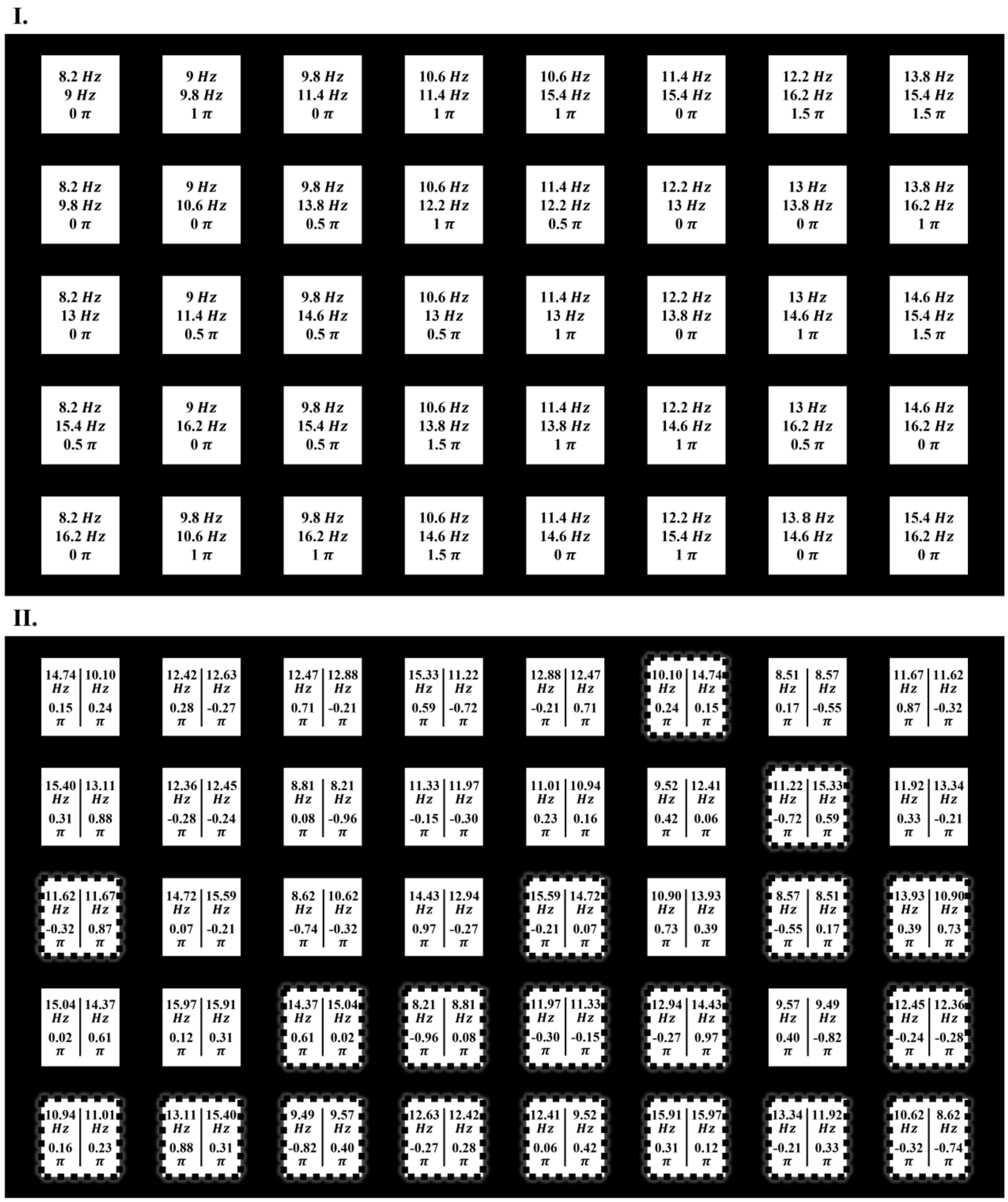

Figure 3. Schematic representation of the stimulus interface with encoding details. **Panel I.** illustrates the frequency-phase encoding scheme used for the CA and BV paradigms, featuring a total of 40 targets organized into 5 rows and 8 columns. **Panel II.** displays the encoding scheme for the BV and BsV paradigms. Here, the stimuli on the left side correspond to those assigned to the left eye, and those on the right to the right eye. The stimuli within dashed boxes indicate the target groups post-frequency swap between the eyes, whereas those within solid boxes represent the original target groups.

Data Acquisition and Processing

For the data acquisition in this study, a NEUROSCAN 64-lead EEG amplifier was employed, adhering to the international 10-20 system for electrode placement. In the case of the CA and BV paradigms, which primarily involve the occipital region, only the nine electrodes located in this area were utilized, specifically Pz, PO5, PO3, POz, PO4, PO6, O1, Oz, and O2. For the BsV paradigm, owing to the broader distribution of significant interclass differences across the brain regions [14], data from all 64 electrodes were collected.

The acquired experimental data underwent a downsampling process to decrease the sampling rate from 1000 Hz to 250 Hz. This was followed by the application of comb filters to eliminate direct current signals and reduce intermediate frequency interference, utilizing the MNE toolbox [26, 27]. The data processing was carried out using MATLAB and the EEGLAB toolkits, known for their computational efficiency [28].

The assessment of the signal-to-noise ratio (SNR) was performed to better evaluate the performance of the dual-band paradigm. The calculations for wideband SNR, narrowband SNR, and intermodulation SNR [13] were conducted as per the following formulas:

$$\begin{cases} \mathrm{SNR}_{\mathrm{Broadband}} = \frac{\sum_{\delta=1}^{h} N(\delta f_1)+N(\delta f_2)}{\sum_{f=5Hz}^{100Hz} N(f)-\sum_{\delta=1}^{5} N(\delta f_1)+N(\delta f_2)} \\ \mathrm{SNR}_{\mathrm{Narrowband}} = \frac{\sum_{\delta=1}^{h}[N(\delta f_1)+N(\delta f_2)]}{\sum_{k=-1/\Delta f}^{1/\Delta f}\sum_{\delta=1}^{h}[N(k\delta f_1\Delta f)+N(k\delta f_2\Delta f)]-\sum_{\delta=1}^{5}[N(\delta f_1)+N(\delta f_2)]} \\ \mathrm{SNR}_{\mathrm{Intermodulation}} = \frac{\sum_{\delta=1}^{h}[N(\delta f_1)+N(\delta f_2)]}{\sum_{a=-h}^{h}\sum_{b=-h}^{h} N(af_1+bf_2)-\sum_{\delta=1}^{5}[N(\delta f_1)+N(\delta f_2)]} \end{cases} \quad (2)$$

In the given study, the variables $\mathrm{SNR}_{\mathrm{Broadband}}$, $\mathrm{SNR}_{\mathrm{Narrowband}}$, and $\mathrm{SNR}_{\mathrm{Intermodulation}}$ denote the values of the wideband SNR, narrowband SNR, and intermodulation SNR, respectively. The terms $f1$ and $f2$ correspond to the combination of stimulus frequencies utilized in the dual-band configuration. The function N indicates the energy associated with these frequency points. The symbol h signifies the number of harmonics considered, which, for this research, is set at five. The parameter Δf, defined as 2 in this study, represents the bandwidth utilized for the narrowband evaluations.

The wideband SNR quantifies the ratio of the energies of $f1$ and $f2$, along with their harmonics, relative to the entire frequency spectrum, thus reflecting the strength of the SSVEP signal. The narrowband SNR, pivotal for SSVEP classification accuracy, is calculated as the ratio of the energy of $f1$ and $f2$, including their harmonics, to the energy within a $4\ Hz$ bandwidth centered around these frequencies.

Furthermore, the intermodulation SNR, which is crucial for assessing the strength of the UIHC specific to dual-band stimuli, is measured as the ratio of the energies of $f1$ and $f2$, and their harmonics, to the energy at the frequency band where UIHC ($af1 + bf2$, where a, b range from -5 to 5) is observed. It is noteworthy that higher values of intermodulation SNR correspond to weaker representations of the UIHC, which implicates its diminished influence in the presence of strong intermodulation components.

SSVEP classification algorithm

To evaluate the quality of the dataset further, classification analysis was performed using established algorithms within the domain. The SSVEP classification algorithms fall into two primary categories: non-training and training-based methods. However, due to the limited adaptation of many algorithms to the dual-frequency paradigm [29], we selected one representative algorithm from each category for our analysis.

For the non-training category, we utilized the Filter Bank Dual-Frequency Canonical Correlation Analysis (FBDCCA) [13]. This method is an adaptation of the classical Filter Bank Canonical Correlation Analysis (FBCCA) [30], specifically modified to handle dual-frequency SSVEP systems. The FBDCCA algorithm enhances the detection of dual-frequency targets by modifying the templates of FBCCA to accommodate dual frequencies.

In the training-based category, we employed the Task-Related Component Analysis (TRCA) algorithm [31]. TRCA enhances classification performance by using training data to compute a null-domain filter, thus optimizing the detection of task-related components.

Additionally, due to the constraints in time length for plotting traditional spectra, we opted to use Canonical Correlation Analysis (CCA) spectra instead. This approach utilizes the correlation values calculated by the CCA algorithm [32], denoted as ρ, plotted against frequency, providing a spectrum-like representation but with higher resolution [33]. The computation of this spectrum is described by the following equation:

$$\rho(f) = CCA[x(t), \cos(2\pi f t)] \quad (3)$$

Here, ρ represents the value on the vertical axis of the CCA spectrum, and f denotes the frequency, ranging from 5 to 35 Hz with increments of 0.1 Hz in our analysis. The variable t represents the time series data.

In addition, we use the ITR metric in measuring the classification accuracy of the SSVEP system, which is calculated as in **Equation 4**. Where T is the length of the selected time window (in seconds), and an additional 0.5 seconds will be used as the target search time to simulate the real situation [3]. n is the number of stimulus targets. p is the classification accuracy, with a value between 0 and 1.

$$ITR = \frac{60}{T+0.5}\left\{\log_2 n + P\log_2 P + (1-P)\log_2\left[\frac{1-P}{n-1}\right]\right\} \quad (4)$$

Data Validation and quality control

Frequency domain analysis validation

To ascertain the integrity of the dataset, we initially engaged in the analysis of time-domain signals and distributions, presenting representative results in **Figure 4**. As depicted in **Figure 4(I)**, the UIHC in the CA paradigm exhibits significant strength, and there is considerable variability both within and between subjects regarding the evoked frequencies. For instance, the subject illustrated in **Figure 4(I)** demonstrated a UIHC at a frequency of 11.6 Hz, calculated as $6 * f1 - 4 * f2$. Conversely, the primary frequencies of the CA paradigm, specifically components $f1$ and $f2$, displayed instability; for example, the 10.6 Hz stimulus in **Figure 4(I)** was nearly imperceptible, yet its second harmonic at 21.2 Hz was pronounced.

The UIHC in the BV paradigm was comparatively less prevalent, and its main frequency component appeared more stable, as evidenced in **Figure 4(II)**. This stability can be attributed to the application of polarized light technology, which effectively prevents the overlap of the two stimulus frequencies before reaching the retina. Nonetheless, the BV paradigm did not eliminate the occurrence of UIHC, as demonstrated by the presence of a 19.6 Hz frequency ($f1 + f2$) in **Figure 4(II)**. These findings align with previous research [13], underscoring the high quality of the dataset.

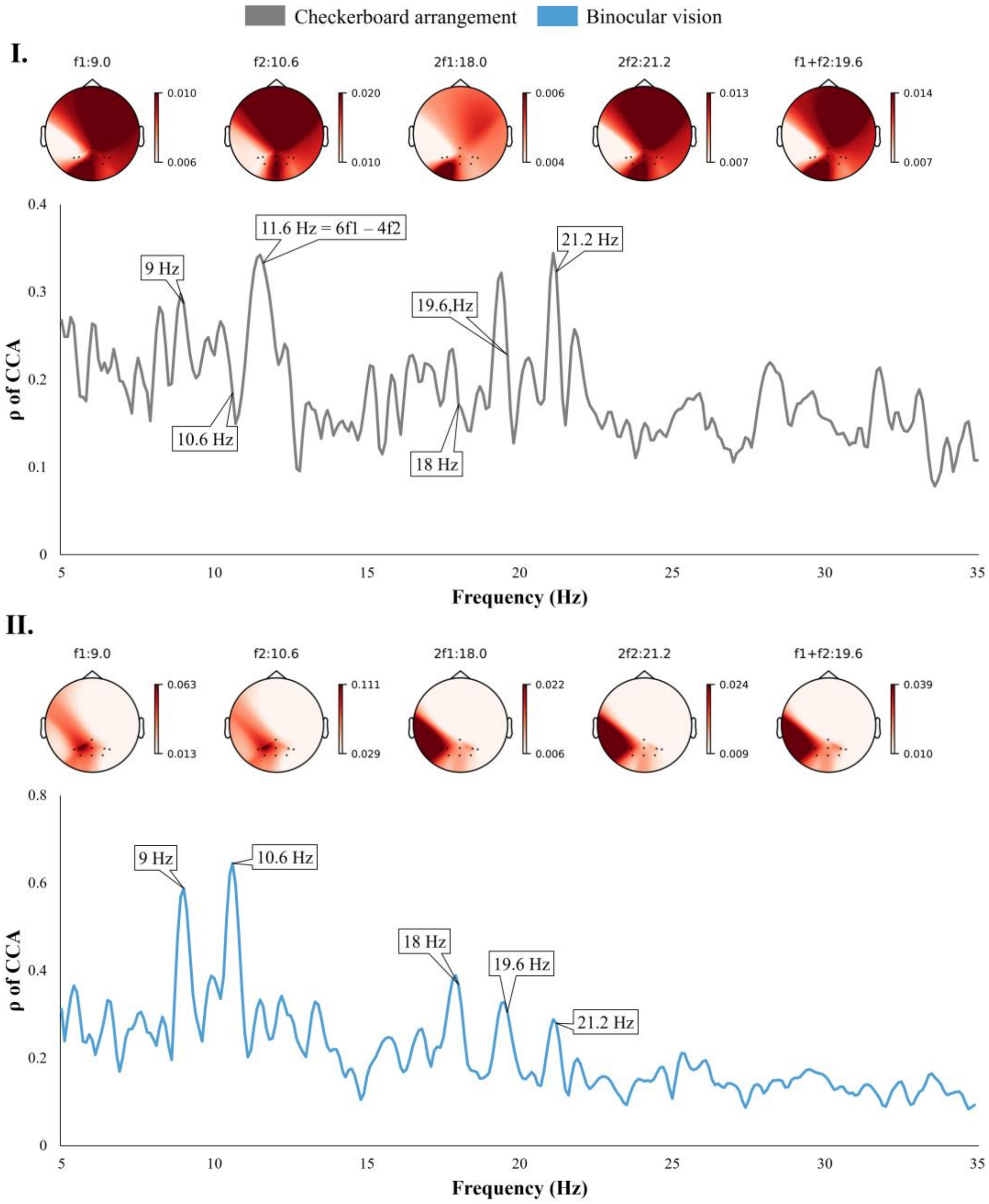

Figure 4. CCA spectra and normalized PSD topography for the CA and BV paradigms at frequencies $f1$ of $9.0\ Hz$ and $f2$ of $10.6\ Hz$. **Panel I.** Gray lines denote the results from the CA analysis, sourced from CV paradigm group subject 01. **Panel II.** Blue lines denote the results from the BV paradigm analysis, sourced from BV paradigm group subject 01.

For the BsV paradigm, evaluations were conducted independently due to its distinct encoding approach and the acquisition of a more extensive array of leads. **Figure 5** illustrates typical frequency domain and topographic map schematics; **Figure 5(I)** displays the left eye stimulus analysis results at frequency $f1$ of $10.9\ Hz$ and the right eye at frequency $f2$ of $13.93\ Hz$, while **Figure 5(II)** presents the inverse. These results highlight that the frequency characteristics evoked by these stimulus targets are remarkably similar and nearly identical. However, there is a notable difference in their PSD topography, attributed to the disparate allocation of visual resources between the two eyes. This differential resource distribution underscores the efficacy of the BsV paradigm in performing classifications.

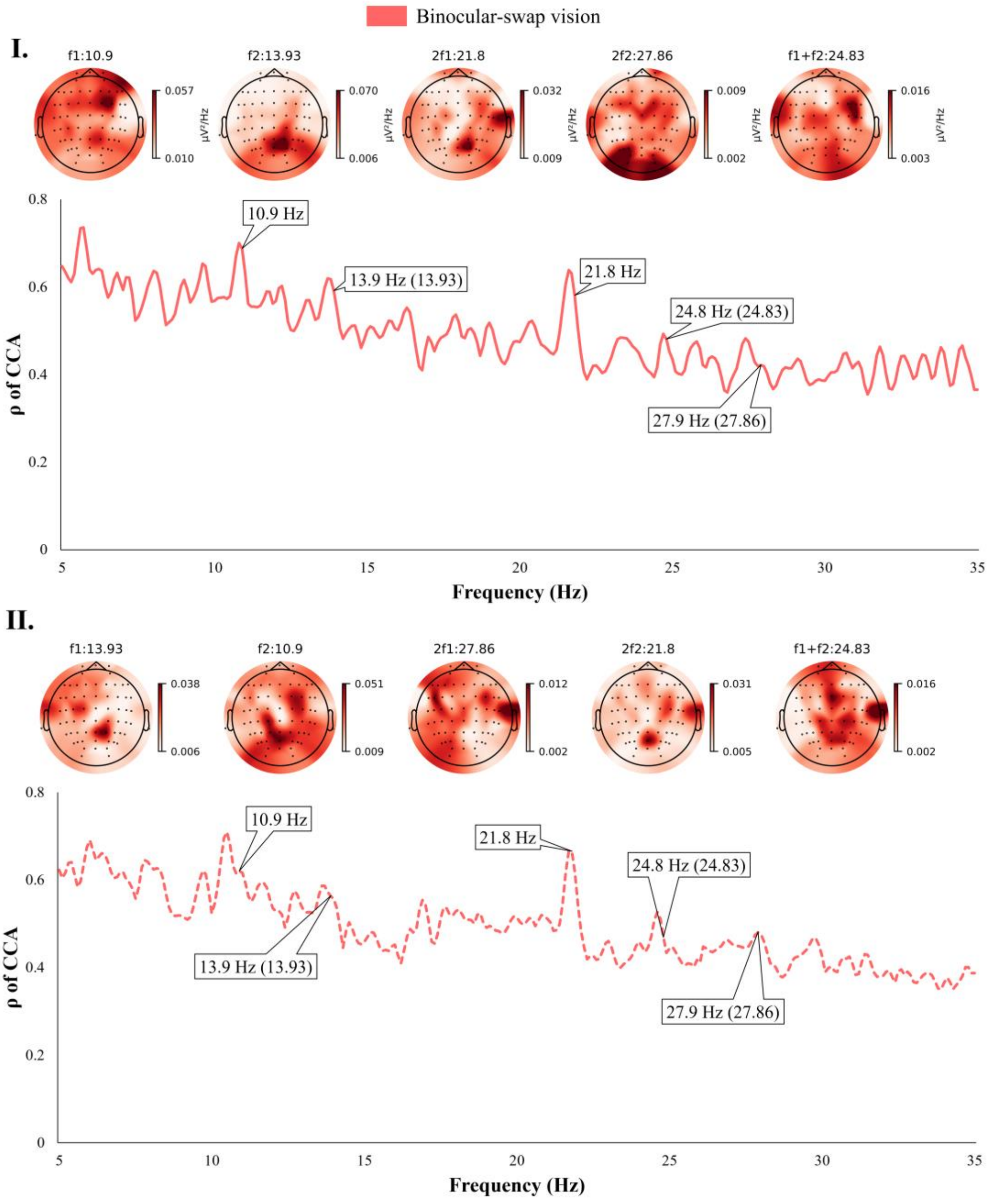

Figure 5. CCA spectra of the BsV paradigm with normalized PSD topography. **Panel I.** Solid lines represent results for a stimulus target with the left eye frequency $f1$ of $13.93\ Hz$ and the right eye frequency $f2$ of $10.9\ Hz$. **Panel II.** Dashed lines represent results for stimulus targets with the left eye frequency $f1$ of $10.9\ Hz$ and the right eye frequency $f2$ of $13.93\ Hz$. All data sourced from CV paradigm group subject 01.

SNR ratio distribution analysis

To assess the overall quality of the dataset, we computed the wideband SNR, narrowband SNR, and intermodulation SNR for a single trial across each of the three paradigms, with the results depicted in **Figure 6**. The distributions of the SNR ratios for all three paradigms conform to a normal distribution, attesting to the robustness and reliability of the dataset. Notably, the distribution of the BsV paradigm in the intermodulation signal-to-noise ratio exhibited a significant shift. This shift is thought to be associated with the distribution of the dominant eye among the subject population, predominantly right-eyed as detailed in **Supplementary Table 1**. This factor likely influenced the generation of the UIHC, underscoring the dataset's considerable potential for psychological and neurobiological research. Noting that although the BsV paradigm was acquired for 64 leads at the time of acquisition, only data from the nine leads of the occipital region were used in the calculation of SNR as in the other two paradigms.

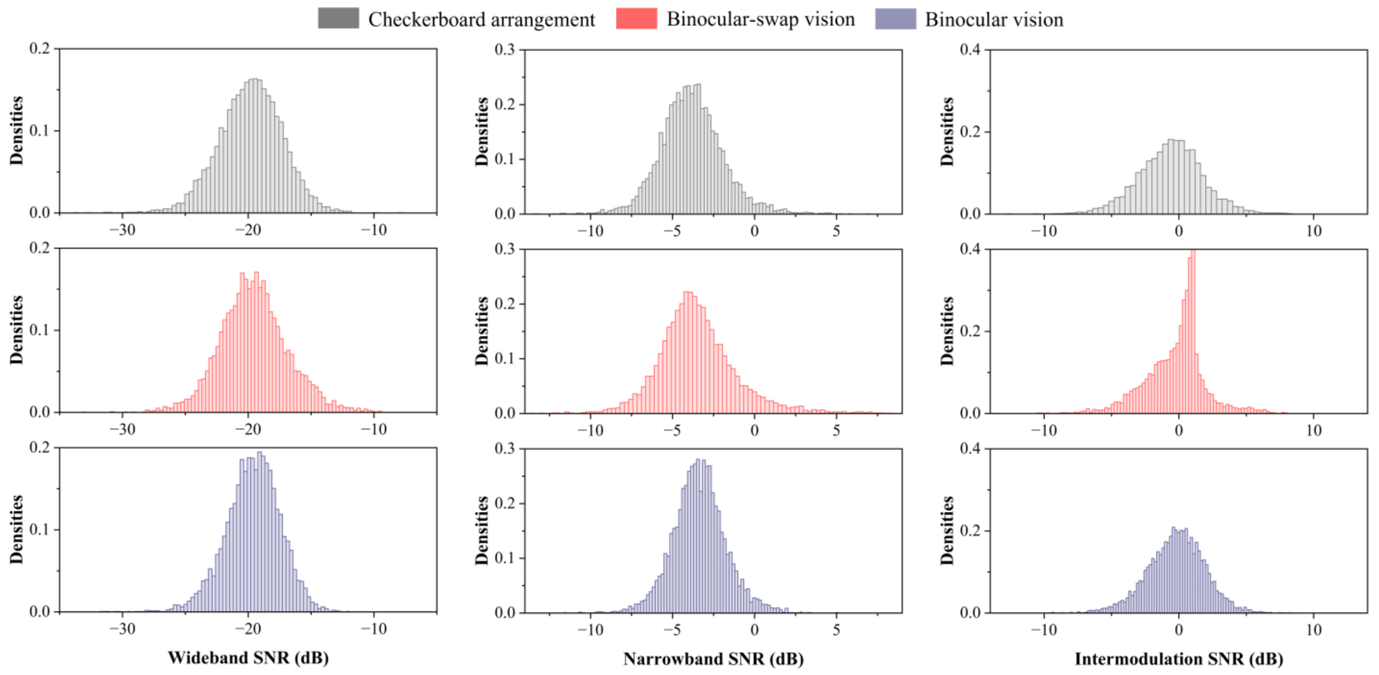

Figure 6. Signal-to-noise ratio distribution for a single trial: Grey represents the CA paradigm, red denotes the BsV paradigm, and blue indicates the BV paradigm. The first column shows the wideband SNR distribution, the second column the narrowband SNR distribution, and the third column the intermodulation SNR distribution.

Average SNR

Further analysis involved calculating the average SNR, with findings presented in **Figure 7**. The BsV paradigm exhibited relatively high values for both wideband and narrowband SNR, followed by the BV paradigm, while the CA paradigm recorded the lowest values, likely due to the instability of the dominant frequency in this paradigm. In terms of intermodulation SNR, both the BV and BsV paradigms outperformed the CA paradigm, suggesting a lower generation of UIHC in these paradigms. These results align with previous research, affirming the dataset's quality [13]. However, it is important to note that both the wideband and narrowband SNR of the current dataset are lower than those reported in single-frequency SSVEP datasets [22, 23], potentially due to the diversion of UIHC for total stimulus response energy.

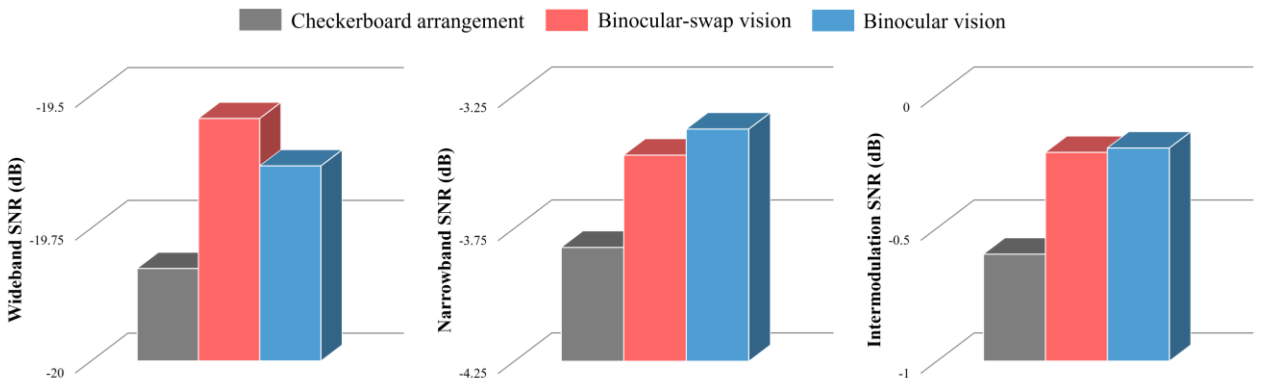

Figure 7. Bar chart of the mean values of wideband SNR, narrowband SNR, and intermodulation SNR: Grey corresponds to the CA paradigm, red to the BsV paradigm, and blue to the BV paradigm.

Classification results without training

Given that the SSVEP paradigm predominantly serves classification tasks, we analyzed the dataset accordingly. For the no-training scenario, we implemented the FBDCCA method. Due to the inherent characteristics of the BsV paradigm, which encodes the same for two sets of targets, it precludes the feasibility of no-training classification. Therefore, our analysis was confined to the

CA and BV paradigms, with the findings depicted in Figure 8. It is evident from the figure that the BV paradigm, benefiting from a stable principal frequency, retains some utility even without training. In contrast, the CV paradigm proves virtually inapplicable without training due to significant individual variability in the UIHC. Specific categorization results can be found in **Supplementary Table 2**.

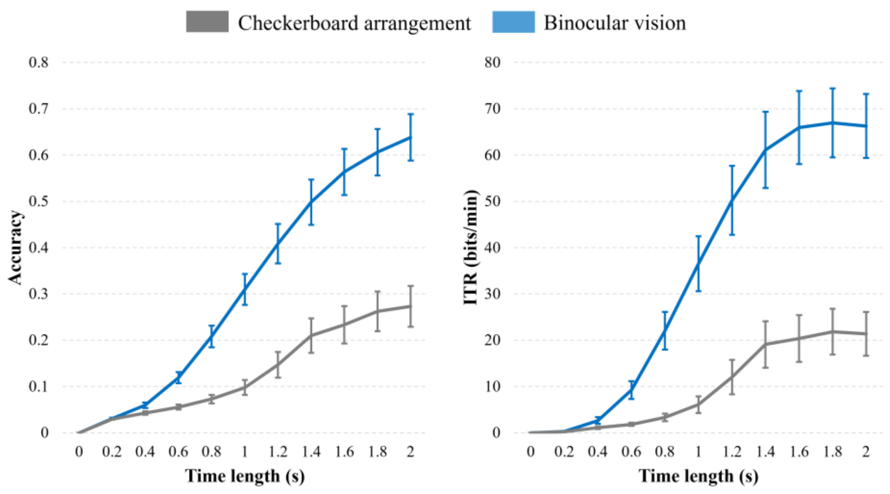

Figure 8. The plot of untrained classification results over time, where blue represents the BV paradigm and grey represents the CA paradigm. The left graph illustrates the correctness curve and the right graph displays the ITR curve. Error bars indicate standard errors.

Classification results with training

Subsequently, we conducted an algorithmic analysis incorporating training, employing the TRCA algorithm within the SSVEP framework. This computation was executed using the leave-one-out approach, utilizing four trials for training and one for testing at each instance. The average outcomes are illustrated in **Figure 9**. The results demonstrate that the performance metrics of correctness and ITR for both the CA and BV paradigms are closely matched, with the CA paradigm slightly outperforming, possibly due to the polarized light technique used in both the BV and BsV paradigms, which reduces light intensity by half. Despite the BsV's close frequency resemblance and its focus primarily on the null domain, it does not match the efficacy of TRCA algorithms. The BsV paradigm is trained and tested with 64-lead data. While there are specialized algorithms enhancing performance in the null domain [14], they do not apply to the other paradigms and thus are not discussed in this paper. Specific categorization results can be found in **Supplementary Table 3**.

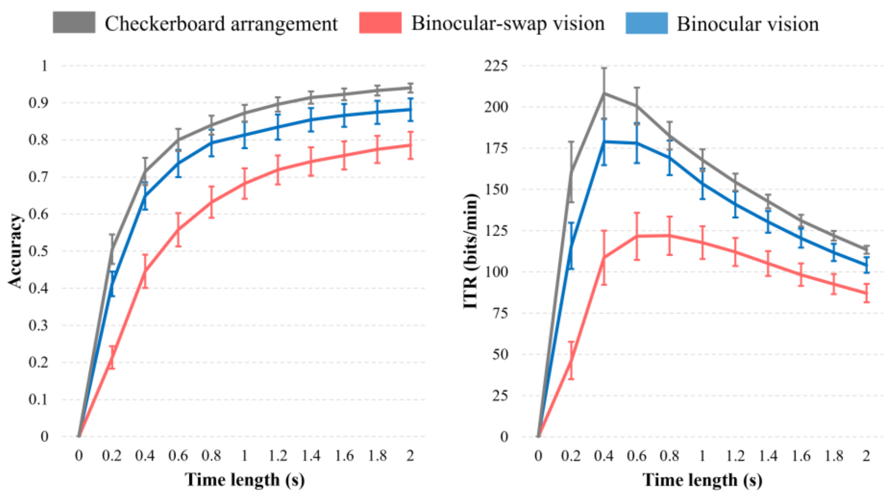

Figure 9. The plot of trained classification results over time, where blue indicates the BV paradigm, red indicates the BsV paradigm, and grey indicates the CA paradigm. The left plot shows the correctness curve and the right plot shows the ITR curve. Error bars are standard errors.

Data Availability

The datasets supporting the results of this paper are available for review at https://cloud.tsinghua.edu.cn/d/8f48deeebb714dd6bd18/ during the review period. This dataset will be submitted to GigaDB once the article is accepted.

Declarations

List of abbreviations

BCI: brain-computer interface

BV: binocular vision

BsV: binocular-swap vision

CA: checkerboard arrangement

CCA: canonical correlation analysis

EEG: electroencephalogram

FBCCA: filter bank canonical correlation analysis

FBDCCA: filter bank dual-frequency canonical correlation analysis

ITR: information transfer rate

SSVEP: steady-state visually evoked potential

TRCA: task-related component analysis

UIHC: unpredictable intermodulation harmonic components

Ethics approval

The data-gathering process of this study was subject to review and subsequently approved by the Medical Ethics Committee of Tsinghua University under the reference number 20180041.

Consent for publication

Not applicable.

Competing interests

The authors declare that they have no competing interests.

Funding

This work is supported by the National Natural Science Foundation of China (U2241208, 62171473), the National Key Research and Development Program of China (2023YFF1205300, 2022YFC3602803), Key Research and Development Program of Ningxia (2023BEG02063).

Authors' contributions

Y.S.: data curation, methodlogy and writing – review & editing. L.L. and Y.L.: data curation, formal analysis. X.C. and X.G.: conceptualization and funding acquisition.

Acknowledgements

The authors would like to thank Yuqing Zhao from the Central Academy of Fine Arts for their help in drawing the pictures in this article.

References

[1] X. Gao, Y. Wang, X. Chen, and S. Gao, "Interface, interaction, and intelligence in generalized brain-computer interfaces," *Trends Cogn Sci,* vol. 25, no. 8, pp. 671-684, Aug 2021, doi: 10.1016/j.tics.2021.04.003.

[2] Y. Sun *et al.*, "A Surgery-Detection Two-Dimensional Panorama of Signal Acquisition Technologies in Brain-Computer Interface," *arXiv preprint arXiv:2308.16102,* 2023, doi: 10.48550/arXiv.2308.16102.

[3] X. Chen, Y. Wang, M. Nakanishi, X. Gao, T. P. Jung, and S. Gao, "High-speed spelling with a noninvasive brain-computer interface," *Proc Natl Acad Sci U S A,* vol. 112, no. 44, pp. E6058-67, Nov 3 2015, doi: 10.1073/pnas.1508080112.

[4] C. S. Herrmann, "Human EEG responses to 1-100 Hz flicker: resonance phenomena in visual cortex and their potential correlation to cognitive phenomena," *Exp Brain Res,* vol. 137, no. 3-4, pp. 346-53, Apr 2001, doi: 10.1007/s002210100682.

[5] H. Wang, T. Li, and Z. Huang, "Remote control of an electrical car with SSVEP-Based BCI," in *2010 ieee international conference on information theory and information security*, 2010: IEEE, pp. 837-840, doi: 10.1109/ICITIS.2010.5689710.

[6] N. Galloway, "Human brain electrophysiology: Evoked potentials and evoked magnetic fields in science and medicine," *The British journal of ophthalmology,* vol. 74, no. 4, p. 255, 1990.

[7] F. B. Vialatte, M. Maurice, J. Dauwels, and A. Cichocki, "Steady-state visually evoked potentials: focus on essential paradigms and future perspectives," *Prog Neurobiol,* vol. 90, no. 4, pp. 418-38, Apr 2010, doi: 10.1016/j.pneurobio.2009.11.005.

[8] H. J. Hwang, D. Hwan Kim, C. H. Han, and C. H. Im, "A new dual-frequency stimulation method to increase the number of visual stimuli for multi-class SSVEP-based brain-computer interface (BCI)," *Brain Res,* vol. 1515, pp. 66-77, Jun 17 2013, doi: 10.1016/j.brainres.2013.03.050.

[9] Z. Yan, X. R. Gao, and S. K. Gao, "Right-and-left visual field stimulation: A frequency and space mixed coding method for SSVEP based brain-computer interface," (in English), *Sci China Inform Sci,* vol. 54, no. 12, pp. 2492-2498, Dec 2011, doi: 10.1007/s11432-011-4503-5.

[10] L. Liang *et al.*, "Optimizing a dual-frequency and phase modulation method for SSVEP-based BCIs," *J Neural Eng,* vol. 17, no. 4, p. 046026, Aug 12 2020, doi: 10.1088/1741-2552/abaa9b.

[11] A. Materka and M. Byczuk, "Alternate half-field stimulation technique for SSVEP-based brain-computer interfaces," *Electronics Letters,* vol. 42, no. 6, pp. 321-322, Mar 16 2006, doi: 10.1049/el:20060171.

[12] K.-K. Shyu, P.-L. Lee, Y.-J. Liu, and J.-J. Sie, "Dual-frequency steady-state visual evoked potential for brain computer interface," *Neuroscience Letters,* vol. 483, no. 1, pp. 28-31, 2010, doi: 10.1016/j.neulet.2010.07.043.

[13] Y. Sun *et al.*, "A Binocular Vision SSVEP Brain-Computer Interface Paradigm for Dual-Frequency Modulation," *IEEE Trans Biomed Eng,* vol. 70, pp. 1172-1181, Oct 5 2022, doi: 10.1109/TBME.2022.3212192.

[14] Y. Sun *et al.*, "Efficient Dual-Frequency Ssvep Brain-Computer Interface System Exploiting Interocular Visual Resource Disparities," *Available at SSRN 4690080,* 2024, doi: 10.2139/ssrn.4690080.

[15] M.-H. Lee *et al.*, "EEG dataset and OpenBMI toolbox for three BCI paradigms: An investigation into BCI illiteracy," *GigaScience,* vol. 8, no. 5, p. giz002, 2019, doi: 10.1093/gigascience/giz002.

[16] G.-Y. Choi, C.-H. Han, Y.-J. Jung, and H.-J. Hwang, "A multi-day and multi-band dataset for a steady-state visual-evoked potential–based brain-computer interface," *GigaScience,* vol. 8, no. 11, p. giz133, 2019, doi: 10.1093/gigascience/giz133.

[17] F. Zhu, L. Jiang, G. Dong, X. Gao, and Y. Wang, "An open dataset for wearable SSVEP-based brain-computer interfaces," *Sensors,* vol. 21, no. 4, p. 1256, 2021, doi: 10.3390/s21041256.

[18] Y.-E. Lee, G.-H. Shin, M. Lee, and S.-W. Lee, "Mobile BCI dataset of scalp-and ear-EEGs with ERP and SSVEP paradigms while standing, walking, and running," *Scientific data,* vol. 8, no. 1, p. 315, 2021, doi: 10.1038/s41597-021-01094-4.

[19] M. Gu, W. Pei, X. Gao, and Y. Wang, "An open dataset for human SSVEPs in the frequency range of 1-60 Hz," *Scientific Data,* vol. 11, no. 1, p. 196, 2024, doi: 10.1038/s41597-024-03023-7.

[20] J. Mu, S. Liu, A. N. Burkitt, and D. B. Grayden, "Multi-frequency steady-state visual evoked potential dataset," *Scientific Data,* vol. 11, no. 1, p. 26, 2024, doi: 10.1038/s41597-023-02841-5.

[21] S. Sadeghi and A. Maleki, "A comprehensive benchmark dataset for SSVEP-based hybrid BCI," *Expert Systems with Applications,* vol. 200, p. 117180, 2022, doi: 10.1016/j.eswa.2022.117180.

[22] Y. Wang, X. Chen, X. Gao, and S. Gao, "A Benchmark Dataset for SSVEP-Based Brain-Computer Interfaces," *IEEE Trans Neural Syst Rehabil Eng*, vol. 25, no. 10, pp. 1746-1752, Oct 2017, doi: 10.1109/TNSRE.2016.2627556.

[23] B. Liu, X. Huang, Y. Wang, X. Chen, and X. Gao, "BETA: A large benchmark database toward SSVEP-BCI application," *Frontiers in neuroscience*, vol. 14, p. 544547, 2020, doi: 10.3389/fnins.2020.00627.

[24] B. Liu, Y. Wang, X. Gao, and X. Chen, "eldBETA: a large eldercare-oriented benchmark database of SSVEP-BCI for the aging population," *Scientific Data*, vol. 9, no. 1, p. 252, 2022, doi: 10.1038/s41597-022-01372-9.

[25] D. H. Brainard, "The Psychophysics Toolbox," (in English), *Spatial Vision*, vol. 10, no. 4, pp. 433-6, 1997, doi: 10.1163/156856897x00357.

[26] A. Gramfort *et al.*, "MEG and EEG data analysis with MNE-Python," *Frontiers in neuroscience*, p. 267, 2013, doi: 10.3389/fnins.2013.00267.

[27] A. Gramfort *et al.*, "MNE software for processing MEG and EEG data," *neuroimage*, vol. 86, pp. 446-460, 2014, doi: 10.1016/j.neuroimage.2013.10.027.

[28] A. Delorme and S. Makeig, "EEGLAB: an open source toolbox for analysis of single-trial EEG dynamics including independent component analysis," *J Neurosci Methods*, vol. 134, no. 1, pp. 9-21, Mar 15 2004, doi: 10.1016/j.jneumeth.2003.10.009.

[29] R. Zerafa, T. Camilleri, O. Falzon, and K. P. Camilleri, "To train or not to train? A survey on training of feature extraction methods for SSVEP-based BCIs," *J Neural Eng*, vol. 15, no. 5, p. 051001, Oct 2018, doi: 10.1088/1741-2552/aaca6e.

[30] X. Chen, Y. Wang, S. Gao, T. P. Jung, and X. Gao, "Filter bank canonical correlation analysis for implementing a high-speed SSVEP-based brain-computer interface," *J Neural Eng*, vol. 12, no. 4, p. 046008, Aug 2015, doi: 10.1088/1741-2560/12/4/046008.

[31] M. Nakanishi, Y. Wang, X. Chen, Y. T. Wang, X. Gao, and T. P. Jung, "Enhancing Detection of SSVEPs for a High-Speed Brain Speller Using Task-Related Component Analysis," *IEEE Trans Biomed Eng*, vol. 65, no. 1, pp. 104-112, Jan 2018, doi: 10.1109/TBME.2017.2694818.

[32] Z. Lin, C. Zhang, W. Wu, and X. Gao, "Frequency recognition based on canonical correlation analysis for SSVEP-based BCIs," *IEEE transactions on biomedical engineering*, vol. 53, no. 12, pp. 2610-2614, 2006, doi: 10.1109/TBME.2006.886577.

[33] X. Zheng *et al.*, "Objective and quantitative assessment of interocular suppression in strabismic amblyopia based on steady-state motion visual evoked potentials," *Vision research*, vol. 164, pp. 44-52, 2019, doi: 10.1016/j.visres.2019.07.003.

Click here to access/download
Supplementary Material
Supplementary materials.docx

IRB approval

Click here to access/download
Supplementary Material
Institutional_Review_Board_approval.pdf

IRB approval English

Click here to access/download
Supplementary Material
Institutional_Review_Board_approval_Translate_to_English.pdf

Click here to access/download
Supplementary Material
Medical_Ethics_Informed_Consent_Form.pdf

Informed consent English

Click here to access/download
Supplementary Material
Medical_Ethics_Informed_Consent_Form_Translate_to_English.pdf

Informed consent doc

Click here to access/download
Supplementary Material
Informed_Consent_Document.pdf

Informed consent doc English

Click here to access/download
Supplementary Material
Informed_Consent_Document_Translate_to_English.pdf

Graphical Abstract

Click here to access/download;Graphical Abstract;Graphical Abstract.tif

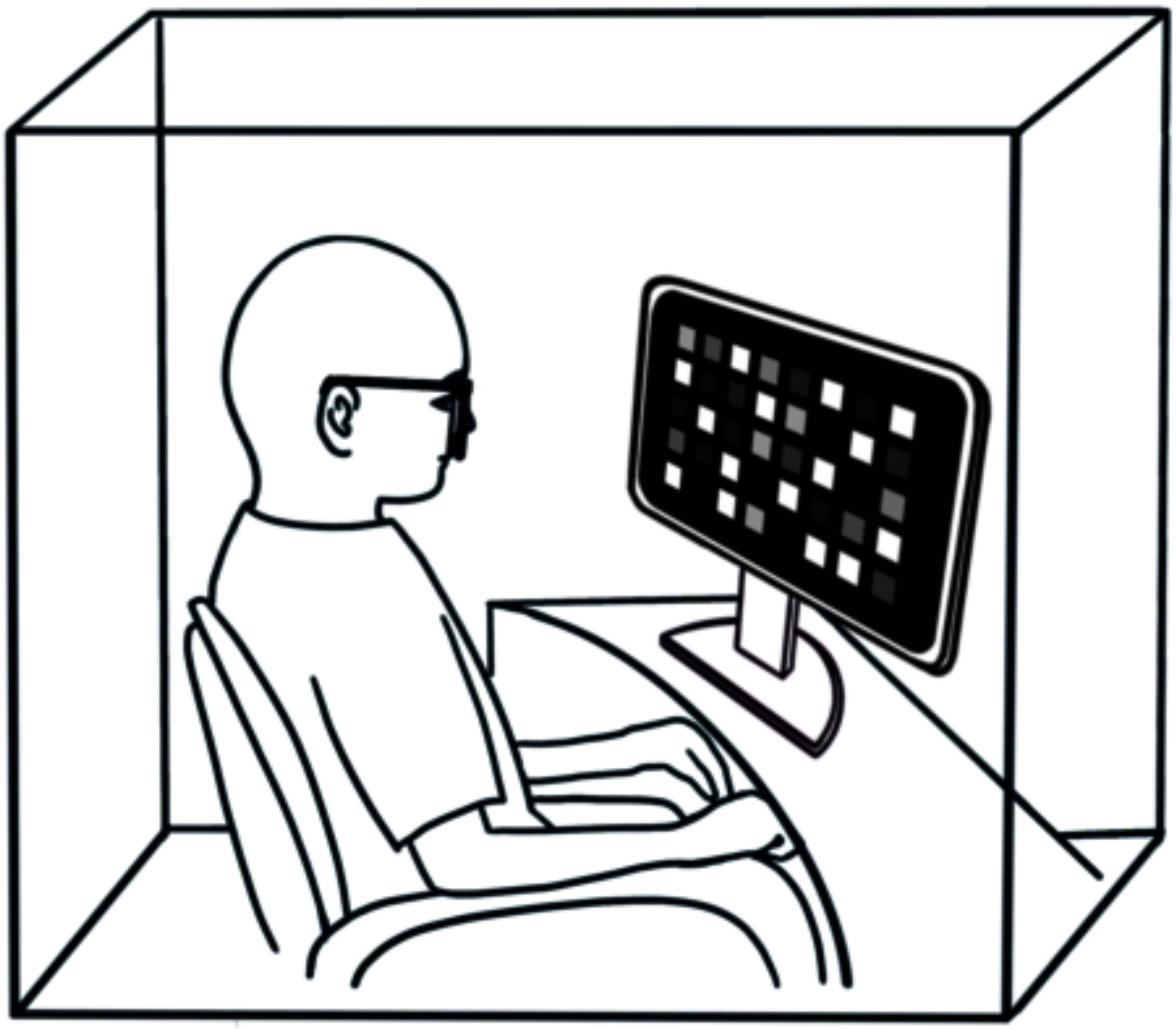

Supplement: giae041_GIGA-D-24-00125_Original_Submission [file giae041_giga-d-24-00125_original_submission.pdf]
