# Supplementary material for: Dual-Alpha: a large EEG study for dual-frequency SSVEP brain–computer interface
Source: Gigascience. 2024 Aug 7;13:giae041. doi: 10.1093/gigascience/giae041 (PMC11304967; doi:10.1093/gigascience/giae041)

# GigaScience


## Dual-Alpha: A Large EEG Study for Dual-Frequency SSVEP Brain-Computer Interface

--Manuscript Draft--

| | |
|---|---|
| **Manuscript Number:** | GIGA-D-24-00125R1 |
| **Full Title:** | Dual-Alpha: A Large EEG Study for Dual-Frequency SSVEP Brain-Computer Interface |
| **Article Type:** | Data Note |
| **Funding Information:** | National Natural Science Foundation of China (U2241208, 62171473) — Dr Yike Sun<br>National Key Research and Development Program of China (2023YFF1205300, 2022YFC3602803) — Dr Yike Sun<br>Key Research and Development Program of Ningxia (2023BEG02063) — Dr Yike Sun |
| **Abstract:** | Background: The domain of brain-computer interface (BCI) technology has experienced significant expansion in recent years. However, the field continues to face a pivotal challenge due to the dearth of high-quality datasets. This lack of robust datasets serves as a bottleneck, constraining the progression of algorithmic innovations and, by extension, the maturation of the BCI field.<br>Findings: This study details the acquisition and compilation of electroencephalogram (EEG) data across three distinct dual-frequency steady-state visual evoked potential (SSVEP) paradigms, encompassing over one hundred participants. Each experimental condition featured 40 individual targets with 5 repetitions per target, culminating in a comprehensive dataset consisting of 21,000 trials of dual-frequency SSVEP recordings. We performed an exhaustive validation of the dataset through signal-to-noise ratio (SNR) analyses and Task-related Component Analysis (TRCA), thereby substantiating its reliability and effectiveness for classification tasks.<br>Conclusions: The extensive dataset presented is set to be a catalyst for the accelerated development of BCI technologies. Its significance extends beyond the BCI sphere and holds considerable promise for propelling research in psychology and neuroscience. The dataset is particularly invaluable for discerning the complex dynamics of binocular visual resource distribution. |
| **Corresponding Author:** | Yike Sun<br>Tsinghua University<br>Beijing, CHINA |
| **Corresponding Author Secondary Information:** | |
| **Corresponding Author's Institution:** | Tsinghua University |
| **Corresponding Author's Secondary Institution:** | |
| **First Author:** | Yike Sun |
| **First Author Secondary Information:** | |
| **Order of Authors:** | Yike Sun |
| | Liyan Liang |
| | Yuhan Li |
| | Xiaogang Chen |
| | Xiaorong Gao |
| **Order of Authors Secondary Information:** | |
| **Response to Reviewers:** | Response to Revision Request<br>Dear Dr. Nogoy,<br>Thank you for your email and the constructive feedback provided by the reviewers |

regarding our manuscript titled "Dual-Alpha: A Large EEG Study for Dual-Frequency SSVEP Brain-Computer Interface" (GIGA-D-24-00125). We are grateful for the opportunity to revise our manuscript and address the concerns raised to enhance its quality and suitability for publication in GigaScience.
We have carefully considered each comment and have undertaken the necessary revisions as suggested. As advised, we have critically reviewed our reference list and reduced the number of self-citations. We now only include five of our previous works that are directly relevant to the current study. This adjustment ensures our references are comprehensive and unbiased, adhering closely to the journal's guidelines. And the RRID identifiers have been included in the revised manuscript.
In accordance with the guidelines outlined in your letter, we have uploaded the revised manuscript file. Additionally, we have included a copy of the original manuscript with all the modifications highlighted in purple for your convenience. Alongside this letter, we have attached our detailed point-by-point response to the reviewers' comments. The comments have been reproduced, and our corresponding responses are provided in a distinct color (purple).
We appreciate the detailed guidance from the reviewers and the editorial team throughout this process. Please do not hesitate to contact us if further information or additional revisions are required.
Thank you for considering our work for publication. We eagerly await your response.
Warm regards,
Professor, Xiaorong Gao, Tsinghua University
Reviewer's Comments:
Reviewer: 1

This study presented a large-scale SSVEP dataset, encompassing over one hundred participants. The acquisition and validation of SSVEP data across CA, BV, and BsV paradigms were detailed introduced. This dataset has the potential to drive the development of SSVEP technology. The following comments are provided to help improve the manuscript before publication.
R: Thanks very much for your constructive comments and for recognizing the potential impact of our large-scale SSVEP dataset. We appreciate the time you have taken to review our manuscript and are grateful for your suggestions, which we believe will significantly enhance the quality and clarity of our paper.
1. Page 1: "In response, a study in 2022 introduced a dual-frequency SSVEP paradigm using 3D display technology, leveraging polarized light to effectively separate the dual frequencies and reduce UIHC generation [13]"
We did not find any relevant introduction or literature annotation regarding the abbreviation "BV" in the "Context" section.
A: Apologies for the oversight in our manuscript. The abbreviation "BV" stands for Binocular Vision. We have now added a clarification to the sentence you referenced. We deeply regret any confusion this may have caused and appreciate your attention to detail.
2. The authors should thoroughly compare and explain the differences between "BV" and "BsV" in the "Stimulus interface and encoding" section.
A: Thank you very much for your suggestion. We have added detailed explanations clarifying the differences between BV and BsV in multiple sections of our manuscript, including "Context," "Participant Information and Experimental Setup," and the "Stimulus Interface and Encoding" section you mentioned. Your feedback has been invaluable and has significantly enhanced the quality of our paper.
3. Page 5: "Panel I. illustrates the frequency-phase encoding scheme used for the CA and BV paradigms …. Panel II. displays the encoding scheme for the BV and BsV paradigms.
Q: Which panel illustrates the BV paradigm, Panel I or Panel II?
A: We apologize for the confusion caused by not clearly differentiating between the BV and BsV paradigms earlier in the manuscript. In fact, the main differences between these two paradigms lie in their encoding and decoding algorithms and the features they focus on, though they appear similar externally during data collection. To clarify, Panel II serves as a shared illustration for both the BV and BsV paradigms. We have amended the text to better explain this and hope it resolves any confusion. Thank you for bringing this to our attention.
4. The parameters of the classification model need to be described in detail in the "SSVEP classification algorithm" section.
A: Thank you for your suggestion. We have now provided a more detailed description

of the algorithm model and its parameters in the relevant section. We hope this addresses your concerns and clarifies any uncertainties.
5. We recommend the authors to include an analysis of the impact of the number of channels on classification accuracy in the "Data Validation and quality control" section.
A: Thank you very much for your suggestion. We have added a subsection at the end of the "Data Validation and Quality Control" section to analyze the relationship between the number of channels and classification accuracy. Your feedback is crucial to enhancing the quality of our paper.

Reviewer: 2

The authors presented a large dual-SSVEP dataset consisting of over 100 participants performing three different stimulation paradigms on a 40-target SSVEP speller interface. This dataset is valuable in terms of its size and novel dual-frequency SSVEP paradigms, which can be used as a binocular dual-SSVEP benchmark.
R: We greatly appreciate your positive feedback on our dataset and your recognition of its contributions to the field of SSVEP research. We are grateful for your supportive and insightful feedback, which encourages us to continue our work with even greater rigor and enthusiasm. We look forward to possibly incorporating any additional suggestions you might have to further enhance the manuscript.
Nonetheless, addressing the following questions can further help readers better understand the dataset.
1. Is there any bad trials detection and rejection step performed? Do all subjects have the same amount of data?
A: Thank you for your inquiry. We maintain strict quality control during data collection in our experiments. Each trial is assessed in real-time, and any problematic trials are immediately recollected. Therefore, the data used in our article should be free from bad trials. Regarding your second question, yes, each subject has an equal amount of data. We have added clarifications in the corresponding sections of our paper to emphasize these points. We hope this addresses your concerns.
2. Figure 4 shows the PSD, which does not seem to have any alpha band noise. Since the alpha rhythm should be common during SSVEP experiments, is baseline correction performed to eliminate the alpha power? How would alpha wave influence the CCA analysis if the stimulating frequency falls in the alpha band range?
A: Thank you very much for your comments. Unfortunately, there may have been some confusion in our description, and I apologize for any uncertainty this may have caused. The graph shown in Figure 4 is not a result of the Power Spectral Density (PSD) analysis, but rather a spectral plot from the Canonical Correlation Analysis (CCA), which represents the distribution of correlation values across frequencies computed by iterating the CCA for each frequency. We opted for this method because our encoding precision is up to 0.1 Hz, and theoretically, a clear PSD would require ten seconds of data, which we do not have. The CCA spectral method enhances frequency resolution, thus providing clearer results.
Regarding your second question, since CCA seeks to identify a linear vector that weights the multi-channel signals to maximize correlation with sine and cosine templates, the influence of large-scale EEG activities, such as alpha waves, is minimal. In each channel, the signals are very similar, so the weighting in this context has a negligible effect, akin to an overall proportional scaling up or down. Consequently, prominent bands like the alpha wave are unlikely to appear distinctly in the CCA spectrum.
Research using the CCA spectrum for analysis is quite sparse, which can indeed lead to confusion. We are currently preparing another paper to clearly explain how this method improves frequency resolution and we hope to extend its application to other signal analysis tasks in various fields.
We have also added an explanation in the relevant section of our article to address this concern. We deeply appreciate your feedback, as it is invaluable in enhancing the quality of our work.
3. Authors performed detailed classification between 40 targets. How about with an NA target (no stimulation at all), since SSVEP detection is also vital for an asynchronous SSVEP system, it would be interesting to study the detection accuracy as well.
A: Thank you very much for your suggestion. We also believe that asynchronous systems are an important direction in this research field. Currently, there is indeed a lack of relevant datasets. We have added a discussion section in the paper to highlight and remind readers of this issue, acknowledging it as one of the current limitations of

Powered by Editorial Manager® and ProduXion Manager® from Aries Systems Corporation

our dataset. Collecting data for an asynchronous system is one of our future research objectives.
Reviewer 3:

This article describes a dual-frequency SSVEP dataset stimulated using checkerboard arrangement, binocular vision, and binocular-swap vision paradigms. 35 participants were recruited for each paradigm. Although the participant groups are different in each paradigm, I agree that this dataset is a valuable asset to the community as, to my knowledge, there is yet a comprehensive binocular vision dual-frequency SSVEP dataset.
R: Thank you for recognizing the value of our dataset and its contribution to the field of SSVEP research. We are glad that the novelty of the dual-frequency SSVEP dataset stimulated through checkerboard arrangement, binocular vision, and binocular-swap vision paradigms is acknowledged as a significant asset to the community. We believe that these clarifications and future research directions will strengthen the manuscript and provide a more comprehensive understanding of the dataset’s implications.
The article is well written in general, however, some further details and edits are needed.
General:
1. The article is missing a Usage Notes section which helps readers to understand how to use this dataset.
A: We agree that a "Usage Notes" section is essential for guiding users on how to effectively utilize the dataset. To this end, we will add a detailed "Usage Notes" section to our manuscript. This section will include information on how to access the dataset, an explanation of the data structure, potential use cases, and recommendations for data handling to assist users in navigating and exploiting the dataset effectively for their research purposes.
2. More details should be presented regarding the data structure. This could be included in the Usage Notes section or README file in your repository, or both.
A: In response to your suggestion, we will provide a comprehensive description of the data structure. This will be included in both the "Usage Notes" section of the manuscript and as a detailed README file in the dataset repository. This documentation will ensure that users can easily understand and manipulate the data according to their research needs.
3. It would be very helpful if you can list the participants who have completed more than 1 paradigm in this study. And list their subject numbers in each paradigm.
A: Thank you for your suggestion. We have added a table in the supplementary materials and dataset to clearly indicate which participants are the same across different paradigms.
Context:
1. The last paragraph is not very well justified. It is not clear why 40-target set up matters. Please consider reorganising this paragraph to highlight why this dataset is valuable and how it is different to the others.
A: Thank you for your feedback. We have completely rewritten the last paragraph to better emphasize the significance of the 40-target dataset and to clarify its unique value compared to other datasets.
Methods:
1. Participant information: please comment on if they have any conditions or impairments with their eyes or any neurological conditions?
A: Thank you very much for your query. None of the participants recruited for our study have any ophthalmic or neurological conditions. We have added this information to the manuscript to clarify.
2. Explain how the participants were recruited and how they were assigned to different groups (paradigms).
A: Thank you for your suggestion. Our dataset was compiled through collaborations across multiple teams, with each team responsible for data collection under a specific paradigm. Hence, participant recruitment for the three paradigms was conducted separately, and there was no process of assigning participants to different groups by us. Any overlap in participants occurred because individuals, who had a keen interest in BCI, voluntarily signed up for multiple experiments. We have not actively assigned participants to different groups. We have now added this information to the manuscript to clarify this process.
3. Table 1: explain everything in the table. It is not clear what you are presenting following the mean age. Is it standard deviation, standard error, or something else?

Powered by Editorial Manager® and ProduXion Manager® from Aries Systems Corporation

A: Thank you very much for your helpful comment. The values presented following the mean age are indeed standard deviations. We have now added this clarification to the appropriate place in Table 1 to ensure this is clear.
4. First line below Table 1: avoid using subjective words and expressions like "the experimental procedure was rigorously designed".
A: We appreciate your suggestion and have revised the statement to remove subjective language. The revised sentence now objectively describes the experimental procedure.
5. Did any of your participants wore glasses? If yes, explain how the polarised glasses work with their own prescription glasses.
A: Thank you very much for your feedback. We had two types of polarized glasses: clip-on and frame-style. For participants who wore glasses, we provided clip-on polarized glasses. For those who did not wear glasses, we provided frame-style polarized glasses. Relevant content has also been added to the text.
6. Stimulation systems: need further details on how the targets were laid out on the screen.
A: We have added additional details regarding the layout of the targets on the screen in the Stimulation Systems section of the manuscript, ensuring that the setup is clearly described for reproducibility.
7. From my own experience, the Psychophysics Toolbox may have lagging issues sometimes and may influence the accuracy of frequency delivery. Did you experience this issue? If yes, any corrections performed?
A: Without seeing your specific code and setup, it's challenging to pinpoint the exact problem. In our experiments, we use photodiodes and other sensors to verify the accuracy of the stimuli before proceeding. So far, I haven't encountered similar issues. From my experience, you might consider preloading the data into the GPU memory and evaluating whether the transmission protocol between the computer and the monitor (we typically use DisplayPort) can support the image refresh rate. Lastly, it's crucial to test and validate the stimulus screen. In my experience, even monitors of the same model can have significant differences in speed of refresh.
8. Include screen refresh rate information in the same place where you introduced the model of the screen and its resolution.
A: We have included the screen refresh rate information alongside the model and resolution of the screen as per your suggestion. This should provide a more comprehensive specification of the display setup.
9. Equation 1 needs more explanation and corrections:
1) What type of rounding was used when converting float to integers?
A: We used floor rounding, and this detail has been added to the text.
2) Should the scaling factor for the cosine function be 0.5 instead of 0.25 so that it can use the full brightness range (0-255)?
A: Apologies, this was a mistake in our writing process. The factor should indeed be 0.5. Thank you very much for pointing that out.
3) 'r' was not explained.
A: Apologies for the oversight. 'r' stands for the refresh rate, and we have now included this explanation in the text.
4) The definition of sigma has problems. From your definition "sigma varies from 1 to 60 multiplied by the stimulation duration", it ranges from 1 to 120 which changes the scale.
A: Thank you for pointing this out. Sigma should indeed be in the numerator. This was a writing error on our part, and we apologize for the confusion.
10. Please provide more details explanation on the differences between BV and BsV.
A: Thank you for your suggestion. We have incorporated detailed explanations clarifying the differences between BV and BsV in several sections of our manuscript, including "Context," "Participant Information and Experimental Setup," and "Stimulus Interface and Encoding". Your feedback has been invaluable and has significantly improved the quality of our paper.
11. The last sentence on light intensity above Figure 2 is unclear. Please further explain.
A: Thank you very much for your suggestion. We have rewritten this sentence to ensure clearer expression.
12. Why not have CA cells be 1*1 pixel as well?
A: From the perspective of writing the paper, the reason for using three pixels is that previous studies have adopted this approach. However, on a personal note, I can tell you that we have tried single-pixel stimulation, but the results were not as effective as

the three-pixel system. Since we haven't conducted a systematic scientific comparison, I cannot provide a definitive answer. However, I suspect that a single pixel may not be spatially distinguishable to the human eye, thus the stimulus sequence might be perceived as an envelope signal rather than a combination of two frequencies.
13. How were the frequencies and phases selected? How were the stimuli frequency and phase layouts determined?
A: Thank you for your question. We directly used the frequency-phase encoding and spatial arrangements from previous research. We have emphasized this point and added the relevant references in the "Stimulus interface and encoding" section.
14. In Figure 3 caption, remove BV on the third line.
A: Thank you very much for your reminder. We have corrected this error in the text.
15. In SNR definitions, delta f definition is not clear.
A: Thank you very much for your reminder. The unclear definition was actually due to a mistake in the formula 2. We have corrected this error and hope this resolves your concern.
16. At the bottom of page 6/15, you mentioned that the data length is too short for plotting the spectra, a potential solution is to zero-pad your data to make it longer for better frequency resolution.
A: Thank you very much for your suggestion. Actually, zero-padding acts as a form of smooth interpolation for the spectrum and does not provide new information. The resolution achieved through zero-padding is only of computational significance, not of actual physical significance. The true improvement in spectral resolution can only be achieved by increasing the effective sample length. For a detailed explanation on this, you can refer to Steven W. Smith's "Digital Signal Processing: A Practical Guide for Engineers and Scientists."
17. The CCA spectra should be further explained with more details.
A: Thank you very much for your suggestion. We have added more details about the CCA spectra. However, it should be noted that this method is currently not widely used, and its underlying principles have not been fully analyzed. Since this is not the main focus of our paper, we have only provided a brief introduction to the method. We are in the process of writing another paper that will systematically summarize and analyze this method.
18. 'x(t)' in equation 3 was not explained.
A: Thank you very much for pointing that out. We have added the relevant information at the corresponding position.
Data validation and quality control:
1. On Figures 4 and 5, suggest labelling the linear combinations and harmonics on the graphs. E.g., 19.6Hz = f1+f2, as how you did with 11.6 Hz.
A: Thank you very much for your suggestion. We have modified the figures accordingly to label the linear combinations and harmonics as you recommended.
2. Figure 4: it would be great if you have participants who did both paradigms and if you can show their data for a more direct comparison.
A: Thank you for your suggestion. Unfortunately, after careful review, we found that there were no participants who completed both the CA and BV paradigms.
3. Figure 4: could you please explain why in the BV paradigm topography plots, why all of them showed strong activity on only one side?
A: This is indeed an interesting observation. I personally believe this is due to a stronger response in the right visual field compared to the left visual field. The underlying cause could be related to habitual eye use or individual differences in neural development. However, this is quite normal. In my personal experience, many people exhibit a lateralized SSVEP response area, and those with responses centered precisely at Oz are actually in the minority.
4. Figure 4 caption has a typo: 'CV' should be changed to 'CA'.
A: Thank you for pointing this out. We have corrected this error.
5. Figure 5: please consider having the topography of the same frequency vertically aligned and have the two waveforms plotted on the same figure to help visualise a direct comparison.
A: Thank you very much for your suggestion. However, I personally believe that this method might not be very intuitive. The overlap between the two waveforms is quite high, and if plotted together, readers might find it difficult to distinguish between them. I have attached an example figure for your reference to illustrate the effect in the word file.
Additionally, if we follow your previous suggestion to label the chart as "f1+f2" and overlay the two graphs, the different f1 and f2 for the two targets might confuse

Powered by Editorial Manager® and ProduXion Manager® from Aries Systems Corporation

| | |
|---|---|
| | readers. For example, regarding 10.9Hz, one graph would show f1 and the other f2. Should I label it as f1 or f2? Or should I annotate it as f1 for Target A and f2 for Target B in the figure? Either way, I feel it would make the readers more confused. I believe the current arrangement is clearer. However, I still greatly appreciate your feedback as it is very important for improving the quality of our paper.<br>6. Third line in SNR ratio distribution analysis: the normal distribution does not support your claim "attesting to the robustness and reliability of the dataset". To show this, you will need to compare to other studies. For example reference 20 if you like.<br>A: Thank you very much for your comments. We realize that our initial statement might be confusing. We have now added a comparison with previous seminal datasets to substantiate our point.<br>7. Average SNR: (second sentence) in narrowband SNR, BV>BsV according to your plot. Suggest running statistical tests on these comparisons. It looks like there might not be any significant differences between the different paradigms.<br>A: Thank you very much for your suggestion. We have incorporated the results of the statistical tests into Figure 7. Although the mean values appear to show only slight differences, the differences are actually statistically significant.<br>8. Figure 7: please update them to have the same scale. The differences are actually quite small, but with the zoomed in view, it may mislead reader to have a false perception.<br>A: Thank you very much for your suggestion. We apologize for any confusion caused by not presenting the statistical test results earlier. In fact, these differences are significant, and the zoomed-in view is necessary to clearly demonstrate these distinctions.<br>9. My rough thoughts on training-free classification on BsV: since differences were observed from the topography, perhaps applying different spatial filters may help in decoding BsV with a training-free method.<br>A: Your idea is quite interesting. However, the challenge lies in the fact that each individual's topographical map is different, with significant individual variability. It is challenging to identify a universal prior spatial filter that works for everyone. Nevertheless, I also believe that exploring methods to achieve training-free BsV classification is a valuable research direction.<br>10. Classification results with training: suggest also perform statistical tests to support your claims such as "The results demonstrate that the performance metrics of correctness and ITR for both the CA and BV paradigms are closely matched".<br>A: Thank you very much for your suggestion. We have now included statistical test results to support our claims.<br>Supplementary Tables:<br>1. Supplementary Table 1 can be combined with Table 1 in the paper.<br>A: We have combined Supplementary Table 1 with Table 1 in the main paper, as suggested. This consolidation should improve the clarity and accessibility of the data presented.<br>2. Supplementary Tables 2 and 3: make the units clear in your tables. Also show standard errors.<br>A: We have updated Supplementary Tables 2 and 3 to ensure that all units are clearly indicated. Additionally, we have included the standard errors for the measurements presented in these tables. These changes should enhance the comprehensibility and accuracy of the data. |
| **Additional Information:** | |
| **Question** | **Response** |
| Are you submitting this manuscript to a special series or article collection? | No |
| **Experimental design and statistics**<br><br>Full details of the experimental design and statistical methods used should be given in the Methods section, as detailed in our Minimum Standards Reporting Checklist. Information essential to interpreting the | Yes |

Powered by Editorial Manager® and ProduXion Manager® from Aries Systems Corporation

| | |
|---|---|
| data presented should be made available in the figure legends.<br><br>Have you included all the information requested in your manuscript? | |
| **Resources**<br><br>A description of all resources used, including antibodies, cell lines, animals and software tools, with enough information to allow them to be uniquely identified, should be included in the Methods section. Authors are strongly encouraged to cite Research Resource Identifiers (RRIDs) for antibodies, model organisms and tools, where possible.<br><br>Have you included the information requested as detailed in our Minimum Standards Reporting Checklist? | Yes |
| **Availability of data and materials**<br><br>All datasets and code on which the conclusions of the paper rely must be either included in your submission or deposited in publicly available repositories (where available and ethically appropriate), referencing such data using a unique identifier in the references and in the “Availability of Data and Materials” section of your manuscript.<br><br>Have you have met the above requirement as detailed in our Minimum Standards Reporting Checklist? | Yes |

Manuscript with DOI
Click here to access/download;Manuscript;Dual Alpha R1.docx

# Dual-Alpha: A Large EEG Study for Dual-Frequency SSVEP Brain-Computer Interface

**Yike Sun[1, +], Liyan Liang[2, +], Yuhan Li[3, 4, +], Xiaogang Chen[3, *] and Xiaorong Gao[1, *]**

Yike Sun sun.yk.bci@outlook.com; Liyan Liang: 18618488256@163.com; Yuhan Li: 13463949502@163.com; Xiaogang Chen: chenxg@bme.cams.cn; Xiaorong Gao: gxr-dea@mail.tsinghua.edu.cn

[1]the School of Biomedical Engineering, Tsinghua University, Beijing, 100084, China.

[2]the China Academy of Information and Communications Technology, Beijing, 100191, China.

[3]Institute of Biomedical Engineering, Chinese Academy of Medical Sciences and Peking Union Medical College, Tianjin, 300192, China.

[4]the School of Life Sciences, Tiangong University, Tianjin, 300387, China.

[*]Correspondence address. Institute of Biomedical Engineering, Chinese Academy of Medical Sciences and Peking Union Medical College, Tianjin, 300192, China. Email: chenxg@bme.cams.cn, and the School of Biomedical Engineering, Tsinghua University, Beijing, 100084, China. Email: gxr-dea@mail.tsinghua.edu.cn.

[+]These authors contributed equally to this work.

**Abstract**

**Background:** The domain of brain-computer interface (BCI) technology has experienced significant expansion in recent years. However, the field continues to face a pivotal challenge due to the dearth of high-quality datasets. This lack of robust datasets serves as a bottleneck, constraining the progression of algorithmic innovations and, by extension, the maturation of the BCI field.

**Findings:** This study details the acquisition and compilation of electroencephalogram (EEG) data across three distinct dual-frequency steady-state visual evoked potential (SSVEP) paradigms, encompassing over one hundred participants. Each experimental condition featured 40 individual targets with 5 repetitions per target, culminating in a comprehensive dataset consisting of 21,000 trials of dual-frequency SSVEP recordings. We performed an exhaustive validation of the dataset through signal-to-noise ratio (SNR) analyses and Task-related Component Analysis (TRCA), thereby substantiating its reliability and effectiveness for classification tasks.

**Conclusions:** The extensive dataset presented is set to be a catalyst for the accelerated development of BCI technologies. Its significance extends beyond the BCI sphere and holds considerable promise for propelling research in psychology and neuroscience. The dataset is particularly invaluable for discerning the complex dynamics of binocular visual resource distribution.

**Keywords:** Brain-computer interface; Dual-frequency; SSVEP; EEG; Dataset.

## Data Description

## Context

Brain-computer interface (BCI) research is currently one of the most vibrant fields of study [1, 2]. Among various BCI technologies, EEG-based interfaces are deemed particularly suitable for consumer electronics applications in sectors like education due to their non-invasive nature and ease of use [3, 4]. Within this domain, steady-state visual evoked potential (SSVEP) based BCIs have emerged as some of the most accurate and stable systems available [5, 6].

SSVEP, or steady-state visually evoked potentials, are frequency-locked and phase-locked brain activities predominantly occurring in the occipital region when an individual observes a flickering light stimulus at a fixed frequency [7]. These signals are extensively utilized in BCI research for functions such as typing and device control. Given that SSVEP responses are typically confined to specific frequency bands [8, 9], dual-frequency SSVEP studies have become a focal point, aiming to enhance the capacity of SSVEP systems to handle more extensive target selections [10]. The exploration of dual-frequency SSVEP represents one of the most promising areas of current research.

Recent years have seen the proposal of various dual-frequency stimulation techniques by researchers, encompassing methods like the Checkerboard arrangement (CA) paradigm [10] and the left-right visual field paradigm , among others. A notable advancement is the enhanced CA introduced in 2020 [11]. However, a persistent challenge across these paradigms is the generation of unpredictable intermodulation harmonic components (UIHC) in the form $a * f1 + b * f2$, where $a$ and $b$ are arbitrary integers [10-13]. Attempts to harness these intermodulation frequencies for coding have largely been unsuccessful due to their instability and individual variability [12, 13].

In response, a study in 2022 introduced a dual-frequency SSVEP paradigm named Binocular vision (BV) using 3D display technology, leveraging polarized light to effectively separate the dual frequencies and reduce UIHC generation [14]. Furthering this approach, the 2024 introduction of the Binocular-swap vision (BsV) paradigm utilizes a similar stimulation strategy but incorporates a specialized coding and decoding algorithm to efficiently utilize the differential visual capacities of the two eyes, making it one of the most effective dual-frequency SSVEP BCI systems to date [15]. Both the BV and BsV paradigms employ identical stimulus and data acquisition methods; however, they differ significantly in their coding schemes. The BV paradigm continues to rely on traditional frequency identification for decoding, whereas the BsV paradigm, facing the presence of targets with identical frequencies, places greater emphasis on the differences in the spatial distribution of dominant eye effects for decoding purposes. Consequently, the BsV paradigm exhibits enhanced potential for coding and decoding within dual-frequency paradigms.

The progression of algorithmic research in BCIs is increasingly leaning towards data-driven approaches, underscoring the critical need for high-quality datasets [16]. There is a plethora of SSVEP datasets covering diverse aspects, including real-world usage scenarios [17], motion-based datasets [18], and multi-frequency SSVEP datasets [19], along with mixed paradigm datasets [20].

However, high-quality datasets specifically crafted for the prevalent 40-target SSVEP input keyboards are notably scarce. This is particularly critical given that one of the primary application of SSVEP technology is currently the development of these 40-target keyboards [21, 22]. Despite this, the field still faces a significant shortage of comprehensive dual-frequency 40-target SSVEP datasets, which are essential for the advancement of BCI technologies. To bridge this gap, we have developed the Dual-Alpha dataset. This dataset is uniquely designed for the three most effective dual-band paradigms—CA, BV, and BsV—and is distinguished as the largest and only dual-frequency SSVEP dataset tailored specifically for 40-target applications.

## Methods

### Participant information and experimental setup

Our study included over one hundred participants. Detailed demographic information is presented in **Table 1**. The experiments for the three paradigms were conducted independently, with voluntary enrollment, and each subject was numbered in the order of enrollment. For the CA paradigm, 35 individuals participated, with an average age of 23.9 years, comprising 22 males and 13 females. In the BsV paradigm, 35 participants were involved, with a mean age of 23.3 years, including 21 males and 14 females. Similarly, in the BV paradigm, 35 participants were involved, with a mean age of 23.2 years, including 23 males and 12 females. Notably, the majority of participants were unfamiliar with SSVEP-based BCI technologies. None of the participants had any ophthalmic or neurological conditions. Some subjects participated in multiple paradigm experiments; this information is provided in **Supplementary Table 1**.

**Table 1.** Participant information statistics

| **Paradigms** | **Number of subjects** | **Average age** ±Standard Deviation | **Gender** | **Dominant eye** |
|---|---|---|---|---|
| Checkerboard Arrangement | 35 | 23.9±3.0 | 22M 13F | |
| Binocular-swap Vision | 35 | 23.3±1.2 | 21M 14F | Left: 9 Right: 26 |
| Binocular Vision | 35 | 23.2±1.8 | 23M 12F | Left: 8 Right: 27 |

As illustrated in **Figure 1**, each participant was seated in a dark, electromagnetically shielded room, maintaining a fixed distance of 80 cm from the stimulus screen. The trial commenced with a 1-second cue period, during which the target for the next stimulus was highlighted in red, allowing the participant to focus. This was followed by a 2-second stimulation period, wherein the participants concentrated solely on the previously cued target. A subsequent 1-second rest period was observed, during which participants were advised to remain still and avoid any movements or blinking. The stimulus and signal acquisition methods for both the BV and BsV paradigms were identical, thus the diagrams of these paradigms are presented in **Figure 1(II)**. Participants wore polarizing glasses throughout the experiments. For subjects who wore glasses, clip-on polarized glasses were used, and for those who did not wear glasses, frame polarized glasses were provided. Each participant underwent a total of 200 trials, with each of the 40 targets being presented in 5 distinct trials. The sequence of stimulus targets was randomized by the computer system to prevent anticipatory biases.

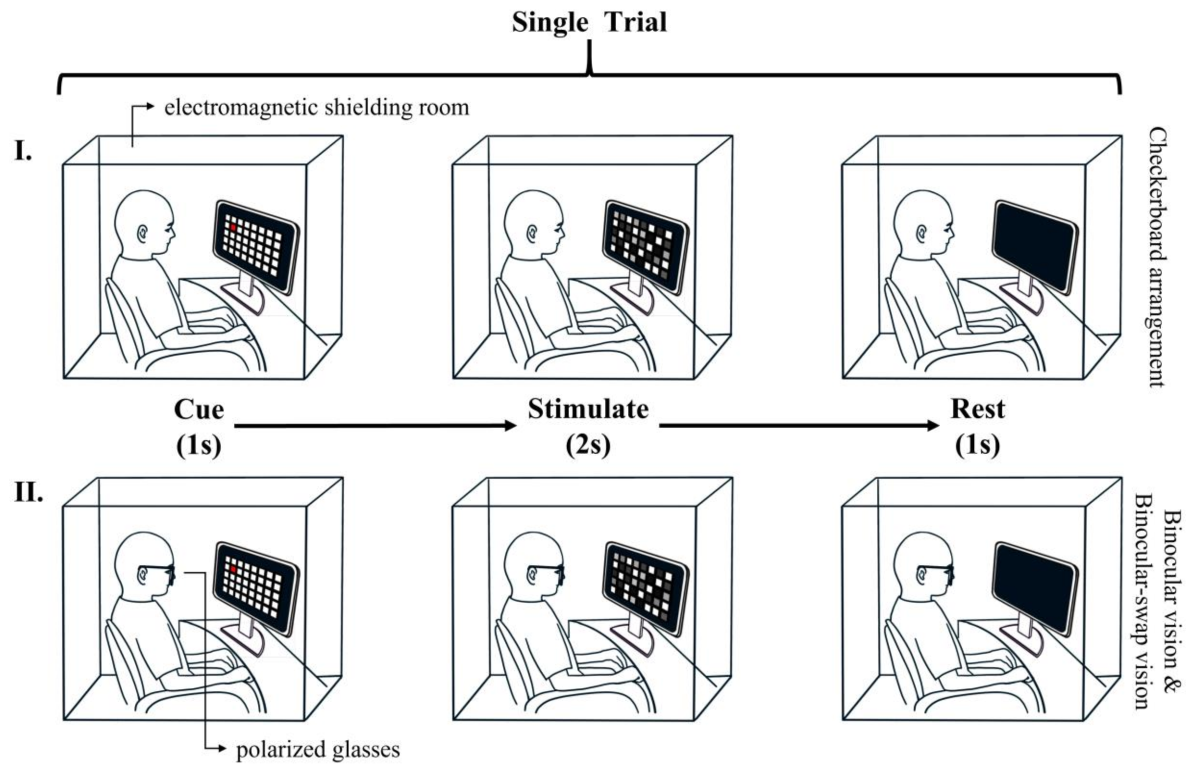


**Figure 1.** Schematic representation of the single-trial flow of the experiment, divided into three phases: cue, stimulate, and rest, lasting 1s, 2s, and 1s respectively. **Panel I** outlines the experimental flow for the CA paradigm. Conversely, **Panel II** provides a common schematic for both the BsV and BV paradigms.

## Stimulation systems

The experimental setup incorporated a stimulation host running on a Windows operating system (NVIDIA GeForce RTX 3080, Intel(R) Core(TM) i7-10700 CPU 2.90GHZ) and utilized a 27-inch stimulation screen (Model D2769Vh, 1920 × 1080 resolution, $60\ Hz$). This screen supports a polarized light 3D display. The stimulus presentation software was developed using MATLAB 2021a (RRID:SCR_001622) in conjunction with the Psychophysics Toolbox version 3 (RRID:SCR_002881) [23].

The configuration of the stimulus targets is depicted in **Figure 2**. The luminance sequences for all the targets were designed based on the Joint Frequency-Phase Modulation (JFPM) technique [24]. In the dual-frequency stimulus configuration, involving frequencies $f1$ and $f2$, the luminance sequences are mathematically expressed as:

$$\boldsymbol{S}(f,\sigma,\varphi) = int\left\{255 * \left[0.5 + 0.5 * \cos\left(\frac{2\pi * f * \sigma}{r} + \varphi\right)\right]\right\} \tag{1}$$

where $\boldsymbol{S}$ denotes the luminance sequence of each frame, with values representing the grey levels on the display ranging from 0 to 255. The variable $\sigma$ represents the number of frames, with the display refresh rate being $60\ Hz$, hence $\sigma$ varies from 1 to 60 multiplied by the stimulation duration. $\varphi$ denotes the phase, and $f$ represents the stimulation frequency. $r$ indicates the display refresh rate.

Regarding the spatial configuration of the stimulus targets, three paradigms are addressed in this study. For the CA paradigm, the stimulus target is illustrated on the left side of **Figure 2(I)** and is structured similarly to a chessboard grid. For the two frequencies $f1$ and $f2$, they are alternated among the stimulus targets, with each small grid measuring 3 pixels, totaling a stimulus target size of 132 × 132 pixels. To human perception, the stimulus target appears as a combination of two distinct frequencies. The stimulus targets were arranged at equal intervals across the screen in the form of 5 rows and 8 columns, and the luminance of the interval portion was always 0. The specific arrangement can be seen in Figure 3.

The BV paradigm and the BsV paradigm constructions are presented on the right side of **Figure 2(I)**. To the human eye, the stimulus appears as a summation of $f1$ and $f2$ frequencies. However, upon closer inspection, the stimuli are interlaced, with only one pixel per line, making the spatial differences imperceptible to the human eye. The demodulation processes for BV and BsV are illustrated in **Figure 2(II)**, where the vibrational phases of the polarized light emitted by $f1$ and $f2$ stimuli differ. These can be re-modulated through the demodulation of polarizing glasses to $f1$ and $f2$, with $f1$ presented to the left eye and $f2$ to the right eye. It is worth noting that the polarized light technique causes each eye to see only half of the pixels on the stimulus screen emitting light, the other half being filtered out by the polarizer due to a phase mismatch. Thus, the intensity of light for stimuli in the BV and BsV paradigms is actually only half that of the CV paradigm.

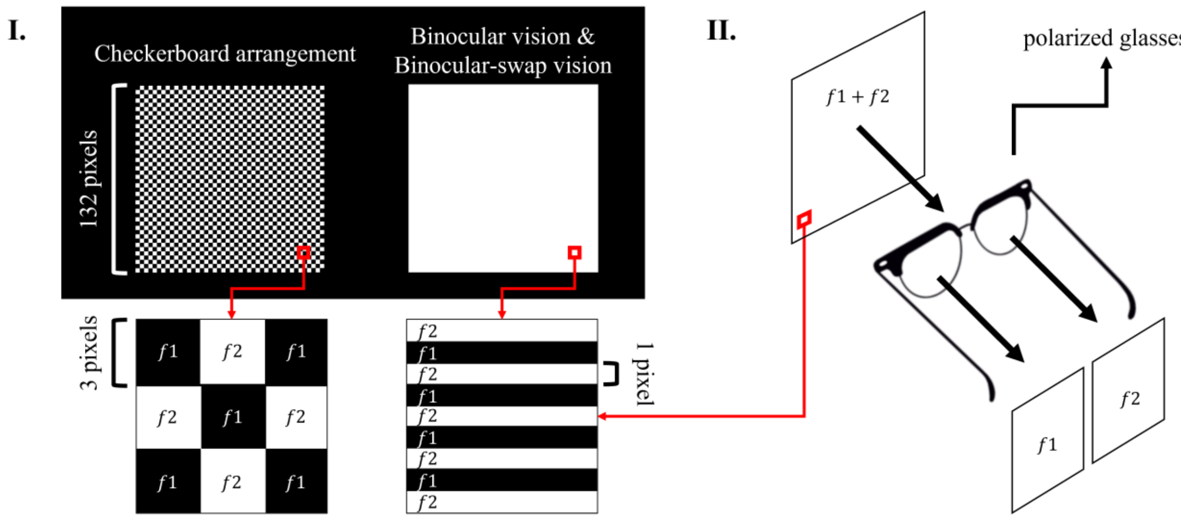


**Figure 2.** Schematic representation of the single-target composition of the dual-frequency SSVEP. **Panel I.** on the left illustrates the single-target composition of the CA paradigm, with a partially zoomed-in schematic showing the alternating frequencies resembling a chessboard grid. **Panel I.** on the right depicts the single-target composition of the BV and BsV paradigms, with a partially zoomed-in view where the difference between the two stimulus frequencies is not directly detectable. **Panel II**. details the demodulation process of the stimulus targets for the BV and BsV paradigms, where the fused frequency combinations $f1$ and $f2$ in the human eye are demodulated by polarized light and displayed to the subject's left and right eyes, respectively.

## Stimulus interface and encoding

**Figure 3** illustrates the stimulus configuration and encoding methodologies employed in this research. The coding of CV and BV paradigms adopts the optimal coding scheme from the study by Liang et al. in 2020 [11]. The BsV paradigm utilize the coding scheme derived using the global optimization method from the study by Sun et al. in 2022 [14]. Among the three paradigms examined, the CV and BV paradigms adhere to traditional frequency-identification coding schemes. These schemes necessitate unique frequency combinations for each target, ensuring that the responses evoked by each target differ significantly in the frequency spectrum. The coding scheme utilized in this study represents the optimal combination of frequencies, as proposed in prior research. The specific arrangement of frequencies and phases, along with their values, is detailed in **Figure 3(I)**.

Conversely, the BsV paradigm leverages the spatial differences arising from the prevalent dominant eye effect in the population to facilitate classification. Within this paradigm, some stimulus targets share identical frequency combinations, resulting in nearly indistinguishable evoked response spectra. However, the assignment of frequencies to the left and right eyes differs (e.g., for Stimulus target A: left eye $f1$, right eye $f2$; for Stimulus target Swap-A: left eye $f2$, right eye $f1$), which creates distinct spatial patterns in the responses that are used for classification tasks. The encoding scheme for the BsV is depicted in **Figure 3(II)**, where targets enclosed in solid borders represent the top 20-group encoding scheme derived from global optimization in previous studies. Targets within dashed borders indicate new target groups resulting from a swap of stimulus frequencies between the left and right eyes.

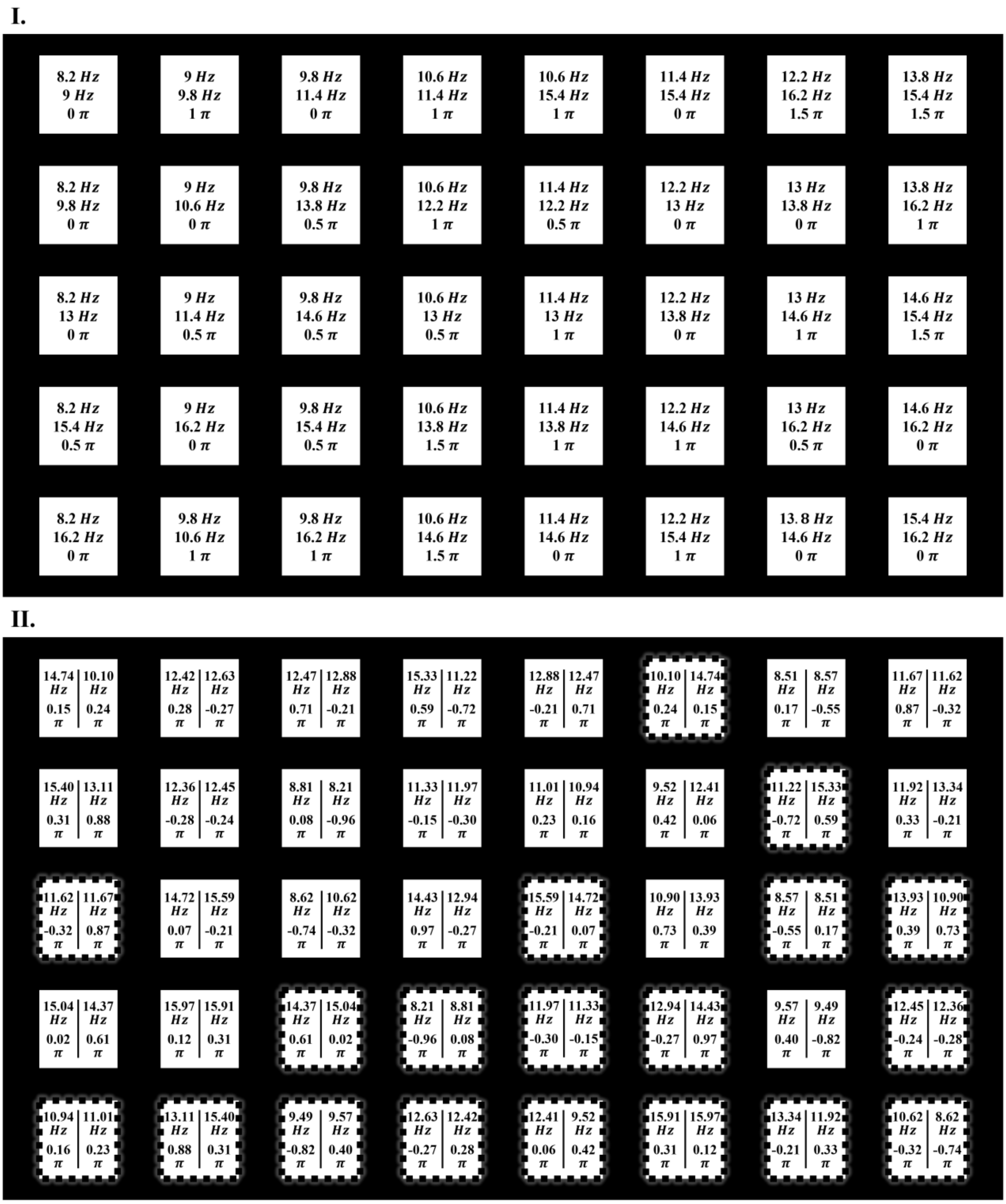


**Figure 3.** Schematic representation of the stimulus interface with encoding details. **Panel I.** illustrates the frequency-phase encoding scheme used for the CA and BV paradigms, featuring a total of 40 targets organized into 5 rows and 8 columns. **Panel II.** displays the encoding scheme for BsV paradigm. Here, the stimuli on the left side correspond to those assigned to the left eye, and those on the right to the right eye. The stimuli within dashed boxes indicate the target groups post-frequency swap between the eyes, whereas those within solid boxes represent the original target groups.

## Data Acquisition and Processing

For the data acquisition in this study, a NEUROSCAN EEG amplifier (RRID:SCR_015818) and a 64-lead Neuroscan Quik-Cap EEG Cap (RRID:SCR_015817) were employed, adhering to the international 10-20 system for electrode placement. In the case of the CA and BV paradigms, which primarily involve the occipital region, only the nine electrodes located in this area were utilized, specifically Pz, PO5, PO3, POz, PO4, PO6, O1, Oz, and O2. For the BsV paradigm, owing to the broader distribution of significant interclass differences across the brain regions [15], data from all 64 electrodes were collected.

The acquired experimental data underwent a downsampling process to decrease the sampling rate from 1000 Hz to 250 Hz. This was followed by the application of comb filters to eliminate direct current signals and reduce intermediate frequency interference, utilizing the MNE toolbox (RRID:SCR_005972) [25, 26]. The data preprocessing was carried out from raw data using the EEGLAB (RRID:SCR_007292) toolkits, known for their computational efficiency [27].

The assessment of the signal-to-noise ratio (SNR) was performed to better evaluate the performance of the dual-band paradigm. The calculations for wideband SNR, narrowband SNR, and intermodulation SNR [14] were conducted as per the following formulas:

$$\begin{cases} \mathrm{SNR}_{\mathrm{Broadband}} = \dfrac{\sum_{\delta=1}^{h} N(\delta f_1)+N(\delta f_2)}{\sum_{f=5Hz}^{100Hz} N(f)-\sum_{\delta=1}^{5} N(\delta f_1)+N(\delta f_2)} \\ \mathrm{SNR}_{\mathrm{Narrowband}} = \dfrac{\sum_{\delta=1}^{h}[N(\delta f_1)+N(\delta f_2)]}{\sum_{k=-f_b/\Delta f}^{f_b/\Delta f} \sum_{\delta=1}^{h}[N(\delta f_1+k\Delta f)+N(\delta+k\Delta f)]-\sum_{\delta=1}^{5}[N(\delta f_1)+N(\delta f_2)]} \\ \mathrm{SNR}_{\mathrm{Intermodulation}} = \dfrac{\sum_{\delta=1}^{h}[N(\delta f_1)+N(\delta f_2)]}{\sum_{a=-h}^{h}\sum_{b=-h}^{h} N(af_1+bf_2)-\sum_{\delta=1}^{5}[N(\delta f_1)+N(\delta f_2)]} \end{cases} \quad (2)$$

In the given study, the variables $\mathrm{SNR}_{\mathrm{Broadband}}$, $\mathrm{SNR}_{\mathrm{Narrowband}}$, and $\mathrm{SNR}_{\mathrm{Intermodulation}}$ denote the values of the wideband SNR, narrowband SNR, and intermodulation SNR, respectively. The terms $f1$ and $f2$ correspond to the combination of stimulus frequencies utilized in the dual-band configuration. The function $N$ indicates the energy associated with these frequency points. The symbol $h$ signifies the number of harmonics considered, which, for this research, is set at five. The parameter $f_b$, defined as 2 in this study, represents the bandwidth utilized for the narrowband evaluations. $\Delta f$ is the reciprocal of the sampling time length of the signal, which is $1/2$ in this study.

The wideband SNR quantifies the ratio of the energies of $f1$ and $f2$, along with their harmonics, relative to the entire frequency spectrum, thus reflecting the strength of the SSVEP signal. The narrowband SNR, pivotal for SSVEP classification accuracy, is calculated as the ratio of the energy of $f1$ and $f2$, including their harmonics, to the energy within a $4\ Hz$ bandwidth centered around these frequencies.

Furthermore, the intermodulation SNR, which is crucial for assessing the strength of the UIHC specific to dual-band stimuli, is measured as the ratio of the energies of $f1$ and $f2$, and their harmonics, to the energy at the frequency band where UIHC ($af1 + bf2$, where $a$, $b$ range from -5 to 5) is observed. It is noteworthy that higher values of intermodulation SNR correspond to weaker representations of the UIHC, which implicates its diminished influence in the presence of strong intermodulation components.

## SSVEP classification algorithm

To evaluate the quality of the dataset further, classification analysis was performed using established algorithms within the domain. The SSVEP classification algorithms fall into two primary categories: non-training and training-based methods [28]. However, due to the limited adaptation of many algorithms to the dual-frequency paradigm, we selected one representative algorithm from each category for our analysis.

For the non-training category, we utilized the Filter Bank Dual-Frequency Canonical Correlation Analysis (FBDCCA) [14]. This method is an adaptation of the classical Filter Bank Canonical Correlation Analysis (FBCCA) [29], specifically modified to handle dual-frequency SSVEP systems. The FBDCCA algorithm enhances the detection of dual-frequency targets by modifying the templates of FBCCA to accommodate dual frequencies. In our study, we constructed templates using sine-cosine matrices derived from the 1st to 5th harmonics of the two frequencies associated with the stimulus targets. These templates were then processed through a filter bank composed of five band-pass filters, with ranges set at [5, 95 Hz], [12, 95 Hz], [19, 95 Hz], [27, 95 Hz], and [35, 95 Hz]. Subsequently, a Canonical Correlation Analysis (CCA) was performed, and the outputs were linearly weighted to generate the final correlation sequence. The template displaying the highest correlation was identified as the predicted result.

In the training-based category, we employed the Task-Related Component Analysis (TRCA) algorithm [30]. TRCA enhances classification performance by using training data to compute a null-domain filter, thus optimizing the detection of task-related components. In this research, the validation was conducted using the leave-one-out method, and the aggregate results were expressed as mean values. The filter banks were configured with the following frequency ranges: [5, 95 Hz], [12, 95 Hz], [19, 95 Hz], and [27, 95 Hz]. Additionally, an ensemble strategy was implemented for the computations.

Additionally, due to the constraints in time length for plotting traditional spectra, we opted to use CCA spectra instead. This approach utilizes the correlation values calculated by the CCA algorithm [31], denoted as ρ, plotted against frequency, providing a spectrum-like representation but with higher resolution [32]. The method first constructs the desired sine-cosine template and subsequently performs a CCA operation with the corresponding EEG data time series to obtain correlation values. Although this method does not provide phase information, its frequency resolution is higher. We consider that this method sacrifices phase information to enhance frequency resolution. The template $Temple(f,t)$ can be represented by the following equation:

$$Temple(f,t) = \begin{bmatrix} \cos(2\pi f t) \\ \sin(2\pi f t) \end{bmatrix} \tag{3}$$

The computation of this spectrum is described by the following equation:

$$\rho(f) = CCA[x(t), Temple(f,t)] \tag{4}$$

Here, $\rho$ represents the value on the vertical axis of the CCA spectrum, and $f$ denotes the frequency, ranging from $5$ to $35$ $Hz$ with increments of $0.1$ $Hz$ in our analysis. The variable $t$ represents the time series data, and $x(t)$ represents EEG data time series.

In addition, we use the ITR metric in measuring the classification accuracy of the SSVEP system, which is calculated as in **Equation 4**. Where $T$ is the length of the selected time window (in seconds), and an additional 0.5 seconds will be used as the target search time to simulate the real situation. $n$ is the number of stimulus targets. $p$ is the classification accuracy, with a value between 0 and 1.

$$ITR = \frac{60}{T+0.5}\left\{\log_2 n + P\log_2 P + (1-P)\log_2\left[\frac{1-P}{n-1}\right]\right\} \tag{5}$$

## Data Validation and quality control

### Frequency domain analysis validation

To ascertain the integrity of the dataset, we initially engaged in the analysis of time-domain signals and distributions, presenting representative results in **Figure 4**. As depicted in **Figure 4(I)**, the UIHC in the CA paradigm exhibits significant strength, and there is considerable variability both within and between subjects regarding the evoked frequencies. For instance, the subject illustrated in **Figure 4(I)** demonstrated a UIHC at a frequency of $11.6$ $Hz$, calculated as $6*f1-4*f2$. Conversely, the primary frequencies of the CA paradigm, specifically components $f1$ and $f2$, displayed instability; for example, the $10.6$ $Hz$ stimulus in **Figure 4(I)** was nearly imperceptible, yet its second harmonic at $21.2$ $Hz$ was pronounced.

The UIHC in the BV paradigm was comparatively less prevalent, and its main frequency component appeared more stable, as evidenced in **Figure 4(II)**. This stability can be attributed to the application of polarized light technology, which effectively prevents the overlap of the two stimulus frequencies before reaching the retina. Nonetheless, the BV paradigm did not eliminate the occurrence of UIHC, as demonstrated by the presence of a $19.6$ $Hz$ frequency ($f1+f2$) in **Figure 4(II)**. These findings align with previous research [14], underscoring the high quality of the dataset.

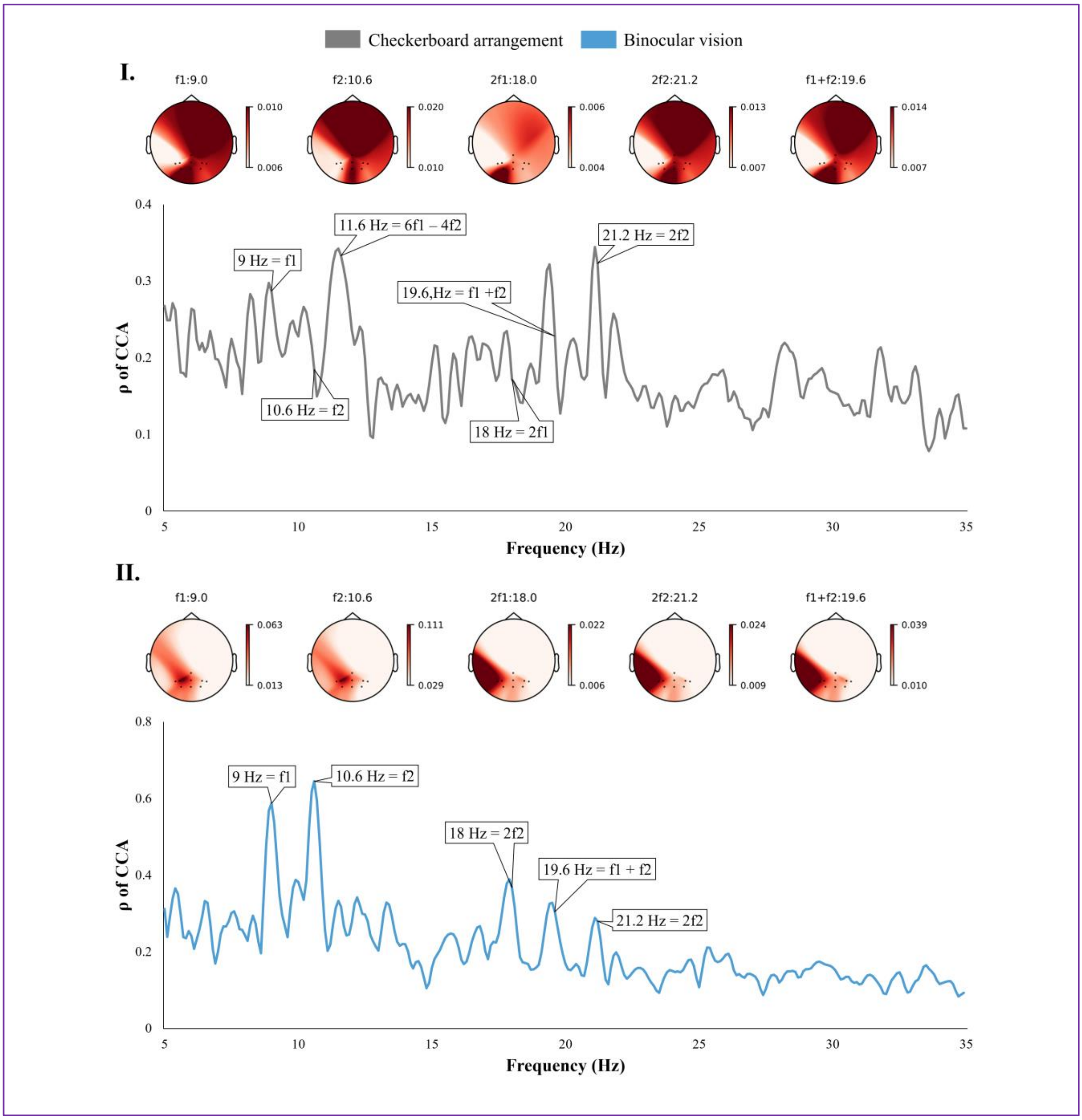


**Figure 4.** CCA spectra and normalized PSD topography for the CA and BV paradigms at frequencies $f1$ of $9.0\ Hz$ and $f2$ of $10.6\ Hz$. **Panel I.** Gray lines denote the results from the CA analysis, sourced from CA paradigm group subject 01. **Panel II.** Blue lines denote the results from the BV paradigm analysis, sourced from BV paradigm group subject 01.

For the BsV paradigm, evaluations were conducted independently due to its distinct encoding approach and the acquisition of a more extensive array of leads. **Figure 5** illustrates typical frequency domain and topographic map schematics; **Figure 5(I)** displays the left eye stimulus analysis results at frequency $f1$ of $10.9\ Hz$ and the right eye at frequency $f2$ of $13.93\ Hz$, while **Figure 5(II)** presents the inverse. These results highlight that the frequency characteristics evoked by these stimulus targets are remarkably similar and nearly identical. However, there is a notable difference in their PSD topography, attributed to the disparate allocation of visual resources between the two eyes. This differential resource distribution underscores the efficacy of the BsV paradigm in performing classifications.

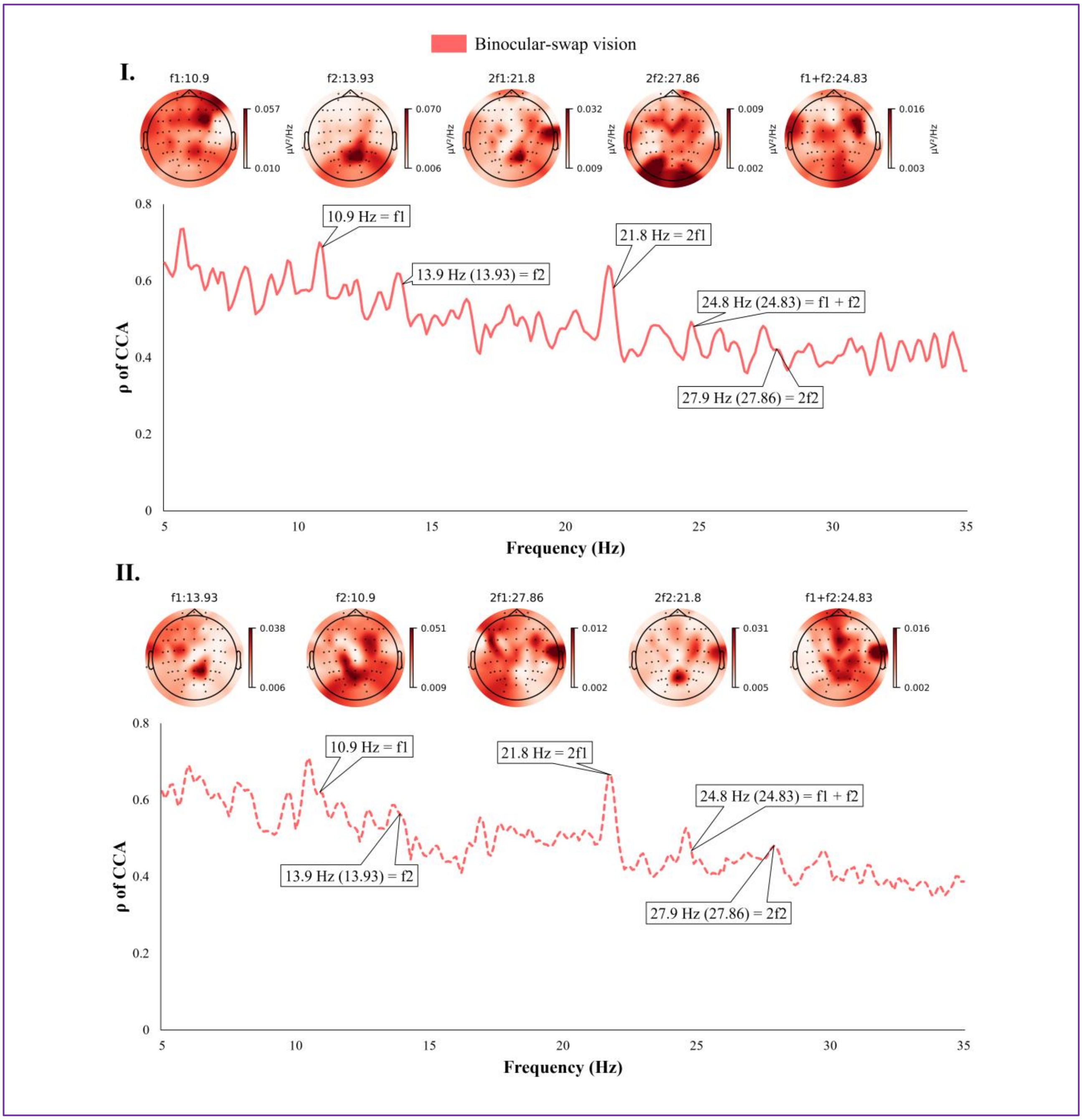


**Figure 5.** CCA spectra of the BsV paradigm with normalized PSD topography. **Panel I.** Solid lines represent results for a stimulus target with the left eye frequency $f1$ of 13.93 $Hz$ and the right eye frequency $f2$ of 10.9 $Hz$. **Panel II.** Dashed lines represent results for stimulus targets with the left eye frequency $f1$ of 10.9 $Hz$ and the right eye frequency $f2$ of 13.93 $Hz$. All data sourced from BsV paradigm group subject 01.

## SNR ratio distribution analysis

To assess the overall quality of the dataset, we computed the wideband SNR, narrowband SNR, and intermodulation SNR for a single trial across each of the three paradigms, with the results depicted in **Figure 6**. When compared to datasets such as Beta [33], our SNR distributions are all normal, but overall more skewed. This skewness correlates with the presence of UIHC in the dual-frequency paradigm, among other factors. Compared to the same multi-frequency dataset study [19], our SNR distributions are very similar. These findings confirm the stability and quality of our dataset. Notably, the distribution of the BsV paradigm in the intermodulation signal-to-noise ratio exhibited a significant shift. This shift is thought to be associated with the distribution of the dominant eye among the subject population, predominantly right-eyed as detailed in **Table 1**. This factor likely influenced the generation of the UIHC, underscoring the dataset's considerable potential for psychological and neurobiological research. Noting

that although the BsV paradigm was acquired for 64 leads at the time of acquisition, only data from the nine leads of the occipital region were used in the calculation of SNR as in the other two paradigms.

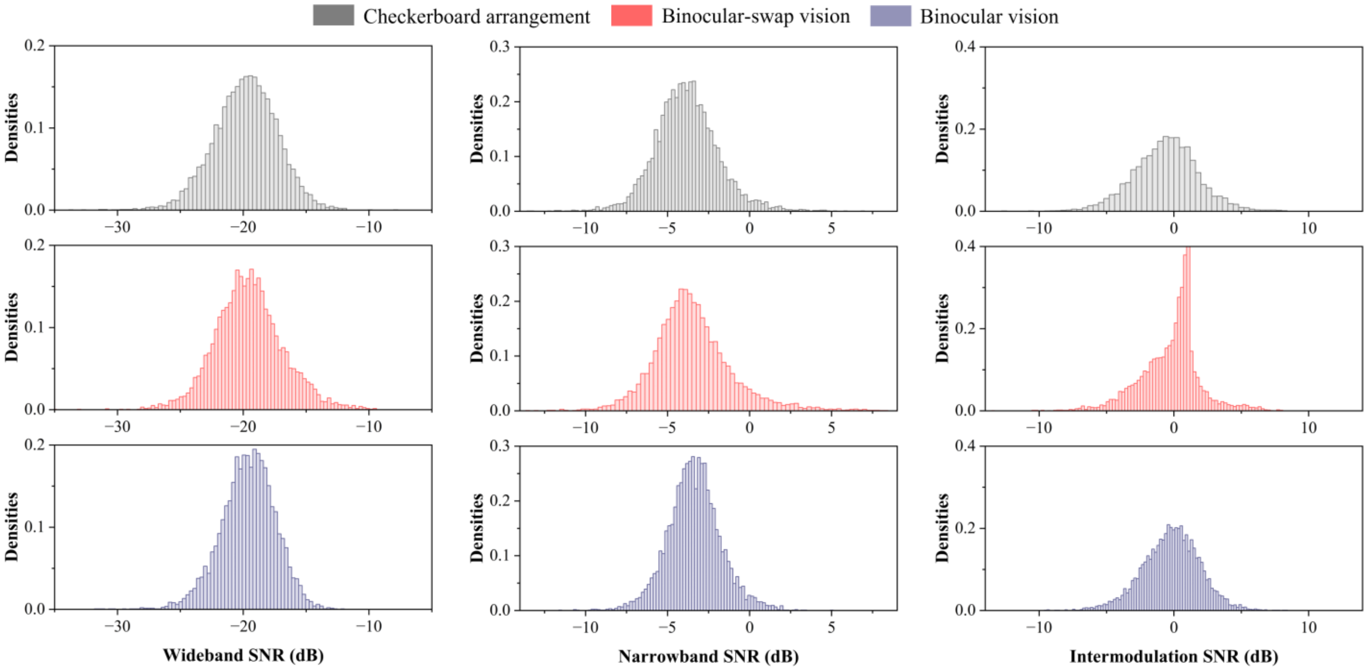


**Figure 6.** Signal-to-noise ratio distribution for a single trial: Grey represents the CA paradigm, red denotes the BsV paradigm, and blue indicates the BV paradigm. The first column shows the wideband SNR distribution, the second column the narrowband SNR distribution, and the third column the intermodulation SNR distribution.

## Average SNR

Further analysis involved calculating the average SNR, with findings presented in **Figure 7**. The BsV paradigm exhibited relatively high values for both wideband and narrowband SNR, followed by the BV paradigm, while the CA paradigm recorded the lowest values, likely due to the instability of the dominant frequency in this paradigm. In terms of intermodulation SNR, both the BV and BsV paradigms outperformed the CA paradigm, suggesting a lower generation of UIHC in these paradigms. These results align with previous research, affirming the dataset's quality [14]. However, it is important to note that both the wideband and narrowband SNR of the current dataset are lower than those reported in single-frequency SSVEP datasets, potentially due to the diversion of UIHC for total stimulus response energy.

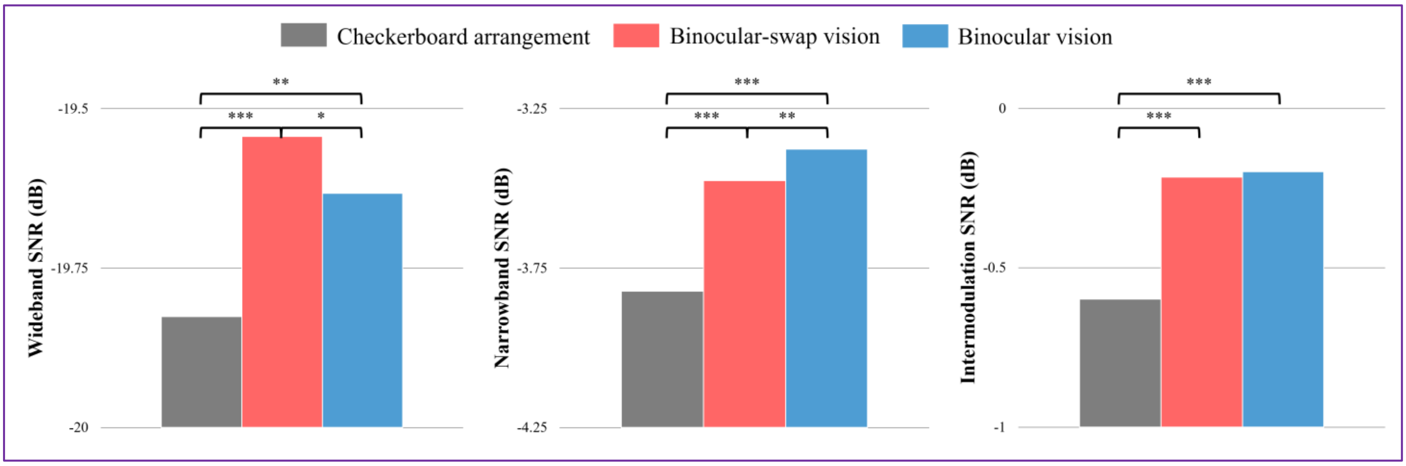


**Figure 7.** Bar chart of the mean values of wideband SNR, narrowband SNR, and intermodulation SNR: Grey corresponds to the CA paradigm, red to the BsV paradigm, and blue to the BV paradigm. The asterisks denote the results of Welch's independent t-tests for significant differences.

Classification results without training

Given that the SSVEP paradigm predominantly serves classification tasks, we analyzed the dataset accordingly. For the no-training scenario, we implemented the FBDCCA method. Due to the inherent characteristics of the BsV paradigm, which encodes the same for two sets of targets, it precludes the feasibility of no-training classification. Therefore, our analysis was confined to the CA and BV paradigms, with the findings depicted in Figure 8. It is evident from the figure that the BV paradigm, benefiting from a stable principal frequency, retains some utility even without training. In contrast, the CV paradigm proves virtually inapplicable without training due to significant individual variability in the UIHC. Specific categorization results can be found in **Supplementary Table 2**.

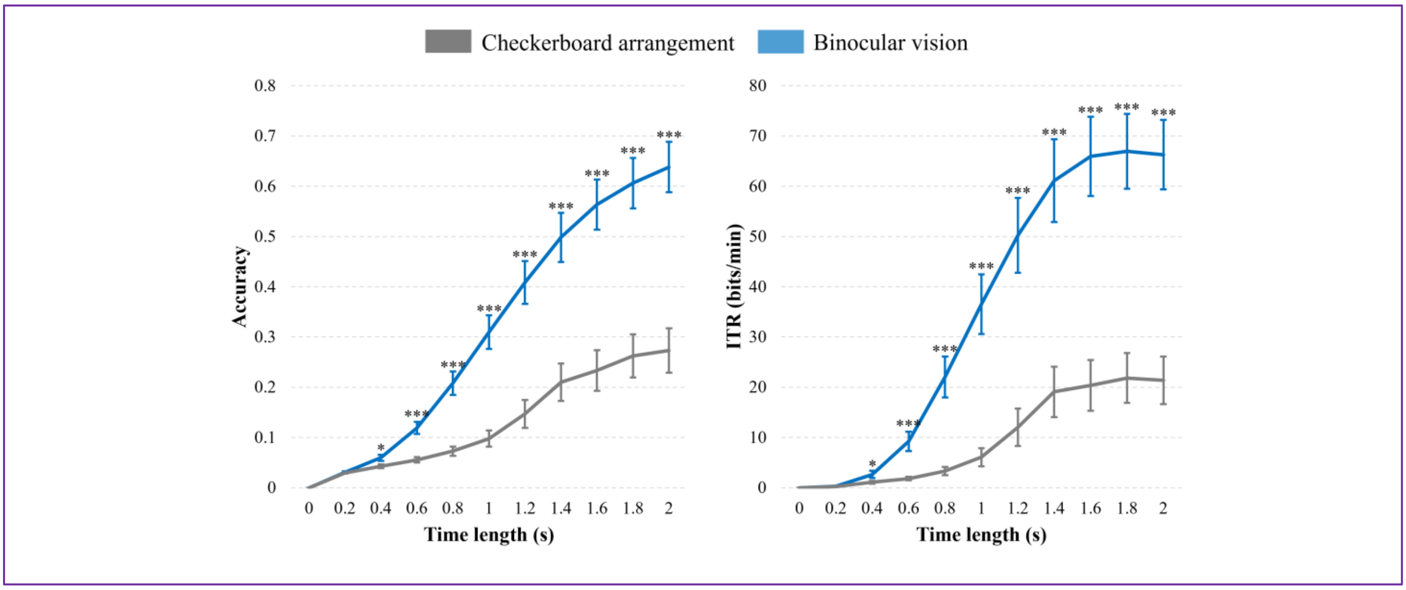


**Figure 8.** The plot of untrained classification results over time, where blue represents the BV paradigm and grey represents the CA paradigm. The left graph illustrates the correctness curve and the right graph displays the ITR curve. Error bars indicate standard errors. The asterisks denote the results of Welch's independent t-tests for significant differences.

Classification results with training

Subsequently, we conducted an algorithmic analysis incorporating training, employing the TRCA algorithm within the SSVEP framework. This computation was executed using the leave-one-out approach, utilizing four trials for training and one for testing at each instance. The average outcomes and the results of statistical tests are illustrated in **Figure 9**. The results indicate that the performance metrics of correctness and ITR for both the CA and BV paradigms are closely matched, with no significant difference observed. The CA paradigm slightly outperformed, possibly due to the polarized light technique used in both the BV and BsV paradigms, which reduces light intensity by half. Despite the BsV's close frequency resemblance and its focus primarily on the null domain, it does not match the efficacy of TRCA algorithms. The BsV paradigm is trained and tested with 64-lead data. While there are specialized algorithms enhancing performance in the null domain [15], they do not apply to the other paradigms and thus are not discussed in this paper. Specific categorization results can be found in **Supplementary Table 3**.

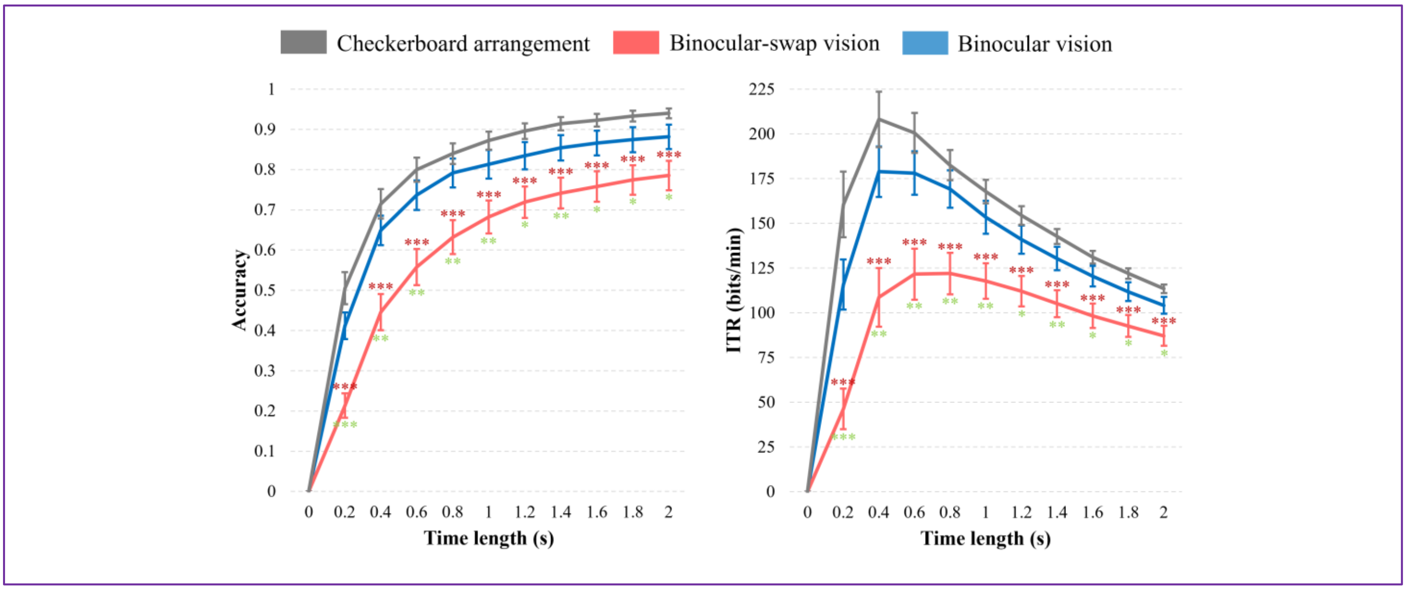


**Figure 9.** The plot of trained classification results over time, where blue indicates the BV paradigm, red indicates the BsV paradigm, and grey indicates the CA paradigm. The left plot shows the correctness curve and the right plot shows the ITR curve. Error bars are standard errors. The green asterisks are the results of Welch's independent t-tests for significant differences between the BV paradigm and the BsV paradigm, and the dark red asterisks are the results of Welch's independent t-tests for significant differences between the CA paradigm and the BsV paradigm. There was no significant difference between the results of the CA and BV paradigms.

## Effect of the number of channels on classification

Lead selection is a critical factor in BCI studies focusing on SSVEP [34]. This study employed a 9-channel acquisition system targeting the occipital region, based on findings from a previous single-frequency study. To determine whether the 9-channel configuration provides non-redundant information across the three paradigms investigated, classification tasks were conducted sequentially using 3-channel (O1, Oz, and O2), 6-channel (PO3, POz, PO4, O1, Oz, and O2), and 9-channel (Pz, PO5, PO3, POz, PO4, PO6, O1, Oz, and O2) setups, utilizing the TRCA algorithm. The results, depicted in **Figure 10**, indicate that the classification accuracy for all three paradigms improves with an increasing number of channels.

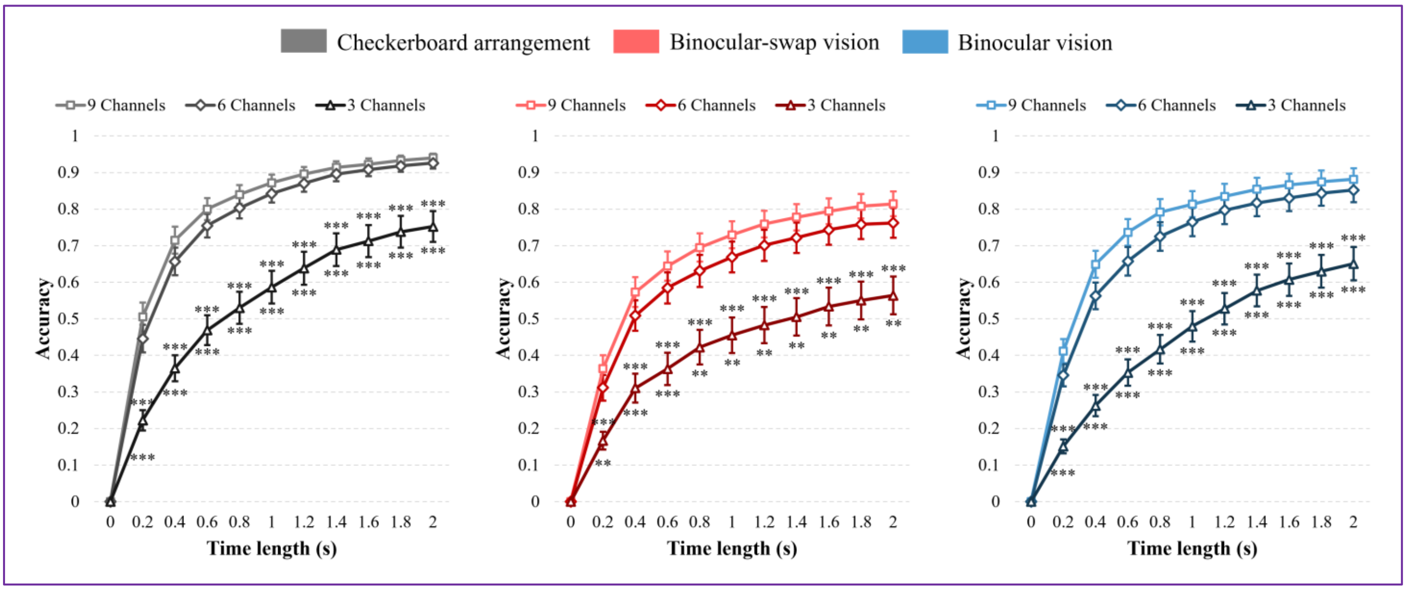


**Figure 10.** Classification accuracy of the three paradigms under the TRCA algorithm for different channel configurations. The left graph represents the CA paradigm, the middle graph represents the BsV paradigm, and the right graph represents the BV paradigm. The channel configurations are: 3-channel, 6-channel, and 9-channel. Results marked with an asterisk indicate significance as

determined by Welch's independent t-test. The asterisks on the top side indicate the results of the statistical test between the 3-channel results and the 9-channel results, and the asterisks on the bottom side indicate the results of the statistical test between the 3-channel results and the 6-channel results. There is no significant difference between the 6-channel results and the 9-channel results.

## Conclusion and discussion

In this study, we introduced the Dual-Alpha dataset, the largest and only dual-frequency SSVEP dataset specifically tailored for 40-target applications. Our comprehensive dataset, encompassing over one hundred participants, underwent rigorous validation through SNR analyses and classification. The validation results highlighted the dataset's high quality and stability.

Despite these strengths, our dataset has certain limitations. One significant issue is the synchronous nature of the data, which restricts its applicability to specific types of BCI systems. The current dataset lacks asynchronous system data, which is crucial for developing more flexible and practical BCI applications[35-37]. Asynchronous systems allow for more natural and spontaneous interactions, better mimicking real-life scenarios where users can switch tasks and modes of operation without predefined time constraints.

Future research should focus on expanding the dataset to include asynchronous system data. This would enable the development of more sophisticated algorithms capable of handling the dynamic and unpredictable nature of real-world BCI applications. Additionally, investigating methods to further reduce the presence of UIHC and enhance the robustness of frequency-locked responses across different paradigms will be essential for advancing research of dual-frequency SSVEP BCI.

# Data Availability

## Data Access

The datasets supporting the results of this paper are available in the GigaScience repository, GigaDB [38].

## Usage Notes

The dataset is organized into three folders, each corresponding to a different paradigm:

*Binocular Vision/*

*Binocular-Swap Vision/*

*Checkerboard Arrangement/*

Each folder contains data files for individual subjects, named in the format "SUBJECT X.csv". The structure of these CSV files is as follows:

*First Column: Number*

*Second Column: Timestamp*

*Third Column: Condition (corresponds to the stimulus code in "Stimulate Code.txt")*

*Fourth Column: Epoch number*

*Subsequent Columns: EEG data, with the first row indicating the name of each lead*

Each CSV file contains EEG data from 5 blocks, covering 40 targets, resulting in a total of 200 trials per subject. The data is sampled at 250 Hz, with timestamps ranging from 0.14 to 2.14 seconds post-stimulus onset. These CSV files can be easily read using the Pandas package in Python (RRID:SCR_018214).

Additionally, each subject has a corresponding information file named "SUBJECT X.txt".

## Declarations

### List of abbreviations

BCI: brain-computer interface

BV: binocular vision

BsV: binocular-swap vision

CA: checkerboard arrangement

CCA: canonical correlation analysis

EEG: electroencephalogram

FBCCA: filter bank canonical correlation analysis

FBDCCA: filter bank dual-frequency canonical correlation analysis

ITR: information transfer rate

SSVEP: steady-state visually evoked potential

TRCA: task-related component analysis

UIHC: unpredictable intermodulation harmonic components

### Ethics approval

The data-gathering process of this study was subject to review and subsequently approved by the Medical Ethics Committee of Tsinghua University under the reference number 20180041.

### Consent for publication

Not applicable.

### Competing interests

The authors declare that they have no competing interests.

### Funding

This work is supported by the National Natural Science Foundation of China (U2241208, 62171473), the National Key Research and Development Program of China (2023YFF1205300, 2022YFC3602803), Key Research and Development Program of Ningxia (2023BEG02063).

### Authors' contributions

Y.S.: data curation, methodlogy and writing – review & editing. L.L. and Y.L.: data curation, formal analysis. X.C. and X.G.: conceptualization and funding acquisition.

### Acknowledgements

The authors would like to thank Yuqing Zhao from the Central Academy of Fine Arts for their help in drawing the pictures in this article.

## References

[1] J. R. Wolpaw *et al.*, "Brain-computer interface technology: a review of the first international meeting," *IEEE Trans Rehabil Eng,* vol. 8, no. 2, pp. 164-73, Jun 2000, doi: 10.1109/tre.2000.847807.

[2] D. J. McFarland, D. J. Krusienski, J. Wolpaw, and E. Wolpaw, "BCI signal processing: feature translation," *Brain-computer interfaces: principles and practice,* vol. 8, pp. 147-165, 2012.

[3] Y. Sun *et al.*, "Signal acquisition of brain-computer interfaces: A medical-engineering crossover perspective review," *Fundamental Research,* 2024/04/16 2024, doi: 10.1016/j.fmre.2024.04.011.

[4] U. Chaudhary, N. Birbaumer, and A. Ramos-Murguialday, "Brain–computer interfaces for communication and rehabilitation," *Nature Reviews Neurology,* vol. 12, no. 9, pp. 513-525, 2016, doi: 10.1038/nrneurol.2016.113.

[5] B. Z. Allison, E. W. Wolpaw, and J. R. Wolpaw, "Brain-computer interface systems: progress and prospects," *Expert Rev Med Devices,* vol. 4, no. 4, pp. 463-74, Jul 2007, doi: 10.1586/17434440.4.4.463.

[6] G. R. McMillan, G. Calhoun, M. Middendorf, J. Schnurer, D. Ingle, and V. Nasman, "Direct brain interface utilizing self-regulation of steady-state visual evoked response (SSVER)," in *Proc. RESNA '95 Annual Conf.(Vancouver, BC)*, 1995, pp. 693-5.

[7] C. S. Herrmann, "Human EEG responses to 1-100 Hz flicker: resonance phenomena in visual cortex and their potential correlation to cognitive phenomena," *Exp Brain Res,* vol. 137, no. 3-4, pp. 346-53, Apr 2001, doi: 10.1007/s002210100682.

[8] N. Galloway, "Human brain electrophysiology: Evoked potentials and evoked magnetic fields in science and medicine," *The British journal of ophthalmology,* vol. 74, no. 4, p. 255, 1990.

[9] F. B. Vialatte, M. Maurice, J. Dauwels, and A. Cichocki, "Steady-state visually evoked potentials: focus on essential paradigms and future perspectives," *Prog Neurobiol,* vol. 90, no. 4, pp. 418-38, Apr 2010, doi: 10.1016/j.pneurobio.2009.11.005.

[10] H. J. Hwang, D. Hwan Kim, C. H. Han, and C. H. Im, "A new dual-frequency stimulation method to increase the number of visual stimuli for multi-class SSVEP-based brain-computer interface (BCI)," *Brain Res,* vol. 1515, pp. 66-77, Jun 17 2013, doi: 10.1016/j.brainres.2013.03.050.

[11] L. Liang *et al.*, "Optimizing a dual-frequency and phase modulation method for SSVEP-based BCIs," *J Neural Eng,* vol. 17, no. 4, p. 046026, Aug 12 2020, doi: 10.1088/1741-2552/abaa9b.

[12] A. Materka and M. Byczuk, "Alternate half-field stimulation technique for SSVEP-based brain-computer interfaces," *Electronics Letters,* vol. 42, no. 6, pp. 321-322, Mar 16 2006, doi: 10.1049/el:20060171.

[13] K.-K. Shyu, P.-L. Lee, Y.-J. Liu, and J.-J. Sie, "Dual-frequency steady-state visual evoked potential for brain computer interface," *Neuroscience Letters,* vol. 483, no. 1, pp. 28-31, 2010, doi: 10.1016/j.neulet.2010.07.043.

[14] Y. Sun *et al.*, "A Binocular Vision SSVEP Brain-Computer Interface Paradigm for Dual-Frequency Modulation," *IEEE Trans Biomed Eng,* vol. 70, pp. 1172-1181, Oct 5 2022, doi: 10.1109/TBME.2022.3212192.

[15] Y. Sun *et al.*, "Efficient dual-frequency SSVEP brain-computer interface system exploiting interocular visual resource disparities," *Expert Systems with Applications,* p. 124144, 2024/05/01 2024, doi: 10.1016/j.eswa.2024.124144.

[16] M.-H. Lee *et al.*, "EEG dataset and OpenBMI toolbox for three BCI paradigms: An investigation into BCI illiteracy," *GigaScience,* vol. 8, no. 5, p. giz002, 2019, doi: 10.1093/gigascience/giz002.

[17] G.-Y. Choi, C.-H. Han, Y.-J. Jung, and H.-J. Hwang, "A multi-day and multi-band dataset for a steady-state visual-evoked potential–based brain-computer interface," *GigaScience,* vol. 8, no. 11, p. giz133, 2019, doi: 10.1093/gigascience/giz133.

[18] Y.-E. Lee, G.-H. Shin, M. Lee, and S.-W. Lee, "Mobile BCI dataset of scalp-and ear-EEGs with ERP and SSVEP paradigms while standing, walking, and running," *Scientific data,* vol. 8, no. 1, p. 315, 2021, doi: 10.1038/s41597-021-01094-4.

[19] J. Mu, S. Liu, A. N. Burkitt, and D. B. Grayden, "Multi-frequency steady-state visual evoked potential dataset," *Scientific Data,* vol. 11, no. 1, p. 26, 2024, doi: 10.1038/s41597-023-02841-5.

[20] S. Sadeghi and A. Maleki, "A comprehensive benchmark dataset for SSVEP-based hybrid BCI," *Expert Systems with Applications,* vol. 200, p. 117180, 2022, doi: 10.1016/j.eswa.2022.117180.

[21] S. Kundu and S. Ari, "Brain-computer interface speller system for alternative communication: a review," *IRBM*, vol. 43, no. 4, pp. 317-324, 2022, doi: 10.1016/j.irbm.2021.07.001.

[22] M. Li, D. He, C. Li, and S. Qi, "Brain–computer interface speller based on steady-state visual evoked potential: A review focusing on the stimulus paradigm and performance," *Brain sciences,* vol. 11, no. 4, p. 450, 2021, doi: 10.3390/brainsci11040450.

[23] D. H. Brainard, "The Psychophysics Toolbox," (in English), *Spatial Vision,* vol. 10, no. 4, pp. 433-6, 1997, doi: 10.1163/156856897x00357.

[24] B. Wittevrongel and M. M. Van Hulle, "Spatiotemporal beamforming: A transparent and unified decoding approach to synchronous visual brain-computer interfacing," *Frontiers in neuroscience,* vol. 11, p. 303179, 2017, doi: 10.3389/fnins.2017.00630.

[25] A. Gramfort *et al.*, "MEG and EEG data analysis with MNE-Python," *Frontiers in neuroscience,* p. 267, 2013, doi: 10.3389/fnins.2013.00267.

[26] A. Gramfort *et al.*, "MNE software for processing MEG and EEG data," *neuroimage,* vol. 86, pp. 446-460, 2014, doi: 10.1016/j.neuroimage.2013.10.027.

[27] A. Delorme and S. Makeig, "EEGLAB: an open source toolbox for analysis of single-trial EEG dynamics including independent component analysis," *J Neurosci Methods,* vol. 134, no. 1, pp. 9-21, Mar 15 2004, doi: 10.1016/j.jneumeth.2003.10.009.

[28] R. Zerafa, T. Camilleri, O. Falzon, and K. P. Camilleri, "To train or not to train? A survey on training of feature extraction methods for SSVEP-based BCIs," *J Neural Eng,* vol. 15, no. 5, p. 051001, Oct 2018, doi: 10.1088/1741-2552/aaca6e.

[29] J. Hong and X. Qin, "Signal processing algorithms for SSVEP-based brain computer interface: State-of-the-art and recent developments," *Journal of Intelligent & Fuzzy Systems,* vol. 40, no. 6, pp. 10559-10573, 2021, doi: 10.3233/JIFS-201280.

[30] M. Yang, T.-P. Jung, J. Han, M. Xu, and D. Ming, "A review of researches on decoding algorithms of steady-state visual evoked potentials," *Sheng wu yi xue Gong Cheng xue za zhi= Journal of Biomedical Engineering= Shengwu Yixue Gongchengxue Zazhi,* vol. 39, no. 2, pp. 416-425, 2022, doi: 10.7507/1001-5515.202111066.

[31] Y. Zhang, S. Q. Xie, H. Wang, and Z. Zhang, "Data analytics in steady-state visual evoked potential-based brain–computer interface: A review," *Ieee Sens J,* vol. 21, no. 2, pp. 1124-1138, 2020, doi: 10.1109/JSEN.2020.3017491.

[32] X. Zheng *et al.*, "Objective and quantitative assessment of interocular suppression in strabismic amblyopia based on steady-state motion visual evoked potentials," *Vision research,* vol. 164, pp. 44-52, 2019, doi: 10.1016/j.visres.2019.07.003.

[33] B. Liu, X. Huang, Y. Wang, X. Chen, and X. Gao, "BETA: A large benchmark database toward SSVEP-BCI application," *Frontiers in neuroscience,* vol. 14, p. 544547, 2020, doi: 10.3389/fnins.2020.00627.

[34] L. Meng, J. Jin, and X. Wang, "A comparison of three electrode channels selection methods applied to SSVEP BCI," in *2011 4th international conference on biomedical engineering and informatics (BMEI)*, 2011, vol. 1: IEEE, pp. 584-587, doi: 10.1109/BMEI.2011.6098285.

[35] J. Pan, Y. Li, R. Zhang, Z. Gu, and F. Li, "Discrimination between control and idle states in asynchronous SSVEP-based brain switches: A pseudo-key-based approach," *IEEE Transactions on Neural Systems and Rehabilitation Engineering,* vol. 21, no. 3, pp. 435-443, 2013, doi: 10.1109/TNSRE.2013.2253801.

[36] P. F. Diez, V. A. Mut, E. M. Avila Perona, and E. Laciar Leber, "Asynchronous BCI control using high-frequency SSVEP," *Journal of neuroengineering and rehabilitation,* vol. 8, pp. 1-9, 2011, doi: 10.1186/1743-0003-8-39.

[37] N. Chumerin, N. V. Manyakov, M. Van Vliet, A. Robben, A. Combaz, and M. M. Van Hulle, "Processing and Decoding Steady-State Visual Evoked Potentials for Brain-Computer Interfaces," in *Digital Image and Signal Processing for Measurement Systems*: River Publishers, 2022, pp. 1-33.

[38] Sun Y; Liang L; Li Y; Chen X; Gao X: Supporting data for "Dual-Alpha: A Large EEG Study for Dual-Frequency SSVEP Brain-Computer Interface" GigaScience Database. 2024. https://doi.org/10.5524/102557.

Manuscript

Click here to access/download;Manuscript;Responses for Reviewers.docx

# Response to Revision Request

Dear Dr. Nogoy,

Thank you for your email and the constructive feedback provided by the reviewers regarding our manuscript titled "Dual-Alpha: A Large EEG Study for Dual-Frequency SSVEP Brain-Computer Interface" (GIGA-D-24-00125). We are grateful for the opportunity to revise our manuscript and address the concerns raised to enhance its quality and suitability for publication in GigaScience.

We have carefully considered each comment and have undertaken the necessary revisions as suggested. As advised, we have critically reviewed our reference list and reduced the number of self-citations. We now only include five of our previous works that are directly relevant to the current study. This adjustment ensures our references are comprehensive and unbiased, adhering closely to the journal's guidelines. And the RRID identifiers have been included in the revised manuscript.

In accordance with the guidelines outlined in your letter, we have uploaded the revised manuscript file. Additionally, we have included a copy of the original manuscript with all the modifications highlighted in purple for your convenience. Alongside this letter, we have attached our detailed point-by-point response to the reviewers' comments. The comments have been reproduced, and our corresponding responses are provided in a distinct color (purple).

We appreciate the detailed guidance from the reviewers and the editorial team throughout this process. Please do not hesitate to contact us if further information or additional revisions are required.

Thank you for considering our work for publication. We eagerly await your response.

Warm regards,

Professor, Xiaorong Gao, Tsinghua University

# Reviewer's Comments:

**Reviewer: 1**

This study presented a large-scale SSVEP dataset, encompassing over one hundred participants. The acquisition and validation of SSVEP data across CA, BV, and BsV paradigms were detailed introduced. This dataset has the potential to drive the development of SSVEP technology. The following comments are provided to help improve the manuscript before publication.

**R:** Thanks very much for your constructive comments and for recognizing the potential impact of our large-scale SSVEP dataset. We appreciate the time you have taken to review our manuscript and are grateful for your suggestions, which we believe will significantly enhance the quality and clarity of our paper.

1. Page 1: "In response, a study in 2022 introduced a dual-frequency SSVEP paradigm using 3D display technology, leveraging polarized light to effectively separate the dual frequencies and reduce UIHC generation [13]" We did not find any relevant introduction or literature annotation regarding the abbreviation "BV" in the "Context" section.

**A:** Apologies for the oversight in our manuscript. The abbreviation "BV" stands for Binocular Vision. We have now added a clarification to the sentence you referenced. We deeply regret any confusion this may have caused and appreciate your attention to detail.

2. The authors should thoroughly compare and explain the differences between "BV" and "BsV" in the "Stimulus interface and encoding" section.

**A:** Thank you very much for your suggestion. We have added detailed explanations clarifying the differences between BV and BsV in multiple sections of our manuscript, including "Context," "Participant Information and Experimental Setup," and the "Stimulus Interface and Encoding" section you mentioned. Your feedback has been

invaluable and has significantly enhanced the quality of our paper.

3. Page 5: "Panel I. illustrates the frequency-phase encoding scheme used for the CA and BV paradigms …. Panel II. displays the encoding scheme for the BV and BsV paradigms.

Q: Which panel illustrates the BV paradigm, Panel I or Panel II?

**A:** We apologize for the confusion caused by not clearly differentiating between the BV and BsV paradigms earlier in the manuscript. In fact, the main differences between these two paradigms lie in their encoding and decoding algorithms and the features they focus on, though they appear similar externally during data collection. To clarify, Panel II serves as a shared illustration for both the BV and BsV paradigms. We have amended the text to better explain this and hope it resolves any confusion. Thank you for bringing this to our attention.

4. The parameters of the classification model need to be described in detail in the "SSVEP classification algorithm" section.

**A:** Thank you for your suggestion. We have now provided a more detailed description of the algorithm model and its parameters in the relevant section. We hope this addresses your concerns and clarifies any uncertainties.

5. We recommend the authors to include an analysis of the impact of the number of channels on classification accuracy in the "Data Validation and quality control" section.

**A:** Thank you very much for your suggestion. We have added a subsection at the end of the "Data Validation and Quality Control" section to analyze the relationship between the number of channels and classification accuracy. Your feedback is crucial to enhancing the quality of our paper.

**Reviewer: 2**

The authors presented a large dual-SSVEP dataset consisting of over 100 participants performing three different stimulation paradigms on a 40-target SSVEP speller interface. This dataset is valuable in terms of its size and novel dual-frequency SSVEP paradigms, which can be used as a binocular dual-SSVEP benchmark.

**R:** We greatly appreciate your positive feedback on our dataset and your recognition of its contributions to the field of SSVEP research. We are grateful for your supportive and insightful feedback, which encourages us to continue our work with even greater rigor and enthusiasm. We look forward to possibly incorporating any additional suggestions you might have to further enhance the manuscript.

Nonetheless, addressing the following questions can further help readers better understand the dataset.

1. Is there any bad trials detection and rejection step performed? Do all subjects have the same amount of data?

**A:** Thank you for your inquiry. We maintain strict quality control during data collection in our experiments. Each trial is assessed in real-time, and any problematic trials are immediately recollected. Therefore, the data used in our article should be free from bad trials. Regarding your second question, yes, each subject has an equal amount of data. We have added clarifications in the corresponding sections of our paper to emphasize these points. We hope this addresses your concerns.

2. Figure 4 shows the PSD, which does not seem to have any alpha band noise. Since the alpha rhythm should be common during SSVEP experiments, is baseline correction performed to eliminate the alpha power? How would alpha wave influence the CCA analysis if the stimulating frequency falls in the alpha band range?

**A:** Thank you very much for your comments. Unfortunately, there may have been some confusion in our description, and I apologize for any uncertainty this may have caused. The graph shown in Figure 4 is not a result of the Power Spectral Density (PSD) analysis, but rather a spectral plot from the Canonical Correlation Analysis (CCA), which represents the distribution of correlation values across frequencies computed by iterating the CCA for each frequency. We opted for this method because our encoding precision is up to 0.1 Hz, and theoretically, a clear PSD would require ten seconds of data, which we do not have. The CCA spectral method enhances frequency resolution, thus providing clearer results.

Regarding your second question, since CCA seeks to identify a linear vector that weights the multi-channel signals to maximize correlation with sine and cosine templates, the influence of large-scale EEG activities, such as alpha waves, is minimal. In each channel, the signals are very similar, so the weighting in this context has a negligible effect, akin to an overall proportional scaling up or down. Consequently, prominent bands like the alpha wave are

unlikely to appear distinctly in the CCA spectrum.

Research using the CCA spectrum for analysis is quite sparse, which can indeed lead to confusion. We are currently preparing another paper to clearly explain how this method improves frequency resolution and we hope to extend its application to other signal analysis tasks in various fields.

We have also added an explanation in the relevant section of our article to address this concern. We deeply appreciate your feedback, as it is invaluable in enhancing the quality of our work.

3. Authors performed detailed classification between 40 targets. How about with an NA target (no stimulation at all), since SSVEP detection is also vital for an asynchronous SSVEP system, it would be interesting to study the detection accuracy as well.

**A:** Thank you very much for your suggestion. We also believe that asynchronous systems are an important direction in this research field. Currently, there is indeed a lack of relevant datasets. We have added a discussion section in the paper to highlight and remind readers of this issue, acknowledging it as one of the current limitations of our dataset. Collecting data for an asynchronous system is one of our future research objectives.

**Reviewer 3:**

This article describes a dual-frequency SSVEP dataset stimulated using checkerboard arrangement, binocular vision, and binocular-swap vision paradigms. 35 participants were recruited for each paradigm. Although the participant groups are different in each paradigm, I agree that this dataset is a valuable asset to the community as, to my knowledge, there is yet a comprehensive binocular vision dual-frequency SSVEP dataset.

**R:** Thank you for recognizing the value of our dataset and its contribution to the field of SSVEP research. We are glad that the novelty of the dual-frequency SSVEP dataset stimulated through checkerboard arrangement, binocular vision, and binocular-swap vision paradigms is acknowledged as a significant asset to the community. We believe that these clarifications and future research directions will strengthen the manuscript and provide a more comprehensive understanding of the dataset's implications.

The article is well written in general, however, some further details and edits are needed.

General:

1. The article is missing a Usage Notes section which helps readers to understand how to use this dataset.

**A:** We agree that a "Usage Notes" section is essential for guiding users on how to effectively utilize the dataset. To this end, we will add a detailed "Usage Notes" section to our manuscript. This section will include information on how to access the dataset, an explanation of the data structure, potential use cases, and recommendations for data handling to assist users in navigating and exploiting the dataset effectively for their research purposes.

2. More details should be presented regarding the data structure. This could be included in the Usage Notes section or README file in your repository, or both.

**A:** In response to your suggestion, we will provide a comprehensive description of the data structure. This will be included in both the "Usage Notes" section of the manuscript and as a detailed README file in the dataset repository. This documentation will ensure that users can easily understand and manipulate the data according to their research needs.

3. It would be very helpful if you can list the participants who have completed more than 1 paradigm in this study. And list their subject numbers in each paradigm.

**A:** Thank you for your suggestion. We have added a table in the supplementary materials and dataset to clearly indicate which participants are the same across different paradigms.

Context:

1. The last paragraph is not very well justified. It is not clear why 40-target set up matters. Please consider reorganising this paragraph to highlight why this dataset is valuable and how it is different to the others.

**A:** Thank you for your feedback. We have completely rewritten the last paragraph to better emphasize the significance of the 40-target dataset and to clarify its unique value compared to other datasets.

Methods:

1. Participant information: please comment on if they have any conditions or impairments with their eyes or any neurological conditions?

**A:** Thank you very much for your query. None of the participants recruited for our study have any ophthalmic or

neurological conditions. We have added this information to the manuscript to clarify.

2. Explain how the participants were recruited and how they were assigned to different groups (paradigms).

**A:** Thank you for your suggestion. Our dataset was compiled through collaborations across multiple teams, with each team responsible for data collection under a specific paradigm. Hence, participant recruitment for the three paradigms was conducted separately, and there was no process of assigning participants to different groups by us. Any overlap in participants occurred because individuals, who had a keen interest in BCI, voluntarily signed up for multiple experiments. We have not actively assigned participants to different groups. We have now added this information to the manuscript to clarify this process.

3. Table 1: explain everything in the table. It is not clear what you are presenting following the mean age. Is it standard deviation, standard error, or something else?

**A:** Thank you very much for your helpful comment. The values presented following the mean age are indeed standard deviations. We have now added this clarification to the appropriate place in Table 1 to ensure this is clear.

4. First line below Table 1: avoid using subjective words and expressions like "the experimental procedure was rigorously designed".

**A:** We appreciate your suggestion and have revised the statement to remove subjective language. The revised sentence now objectively describes the experimental procedure.

5. Did any of your participants wore glasses? If yes, explain how the polarised glasses work with their own prescription glasses.

**A:** Thank you very much for your feedback. We had two types of polarized glasses: clip-on and frame-style. For participants who wore glasses, we provided clip-on polarized glasses. For those who did not wear glasses, we provided frame-style polarized glasses. Relevant content has also been added to the text.

6. Stimulation systems: need further details on how the targets were laid out on the screen.

**A:** We have added additional details regarding the layout of the targets on the screen in the Stimulation Systems section of the manuscript, ensuring that the setup is clearly described for reproducibility.

7. From my own experience, the Psychophysics Toolbox may have lagging issues sometimes and may influence the accuracy of frequency delivery. Did you experience this issue? If yes, any corrections performed?

**A:** Without seeing your specific code and setup, it's challenging to pinpoint the exact problem. In our experiments, we use photodiodes and other sensors to verify the accuracy of the stimuli before proceeding. So far, I haven't encountered similar issues. From my experience, you might consider preloading the data into the GPU memory and evaluating whether the transmission protocol between the computer and the monitor (we typically use DisplayPort) can support the image refresh rate. Lastly, it's crucial to test and validate the stimulus screen. In my experience, even monitors of the same model can have significant differences in speed of refresh.

8. Include screen refresh rate information in the same place where you introduced the model of the screen and its resolution.

**A:** We have included the screen refresh rate information alongside the model and resolution of the screen as per your suggestion. This should provide a more comprehensive specification of the display setup.

9. Equation 1 needs more explanation and corrections:

1) What type of rounding was used when converting float to integers?

**A:** We used floor rounding, and this detail has been added to the text.

2) Should the scaling factor for the cosine function be 0.5 instead of 0.25 so that it can use the full brightness range (0-255)?

**A:** Apologies, this was a mistake in our writing process. The factor should indeed be 0.5. Thank you very much for pointing that out.

3) 'r' was not explained.

**A:** Apologies for the oversight. 'r' stands for the refresh rate, and we have now included this explanation in the text.

4) The definition of sigma has problems. From your definition "sigma varies from 1 to 60 multiplied by the stimulation duration", it ranges from 1 to 120 which changes the scale.

**A:** Thank you for pointing this out. Sigma should indeed be in the numerator. This was a writing error on our part, and

we apologize for the confusion.

10. Please provide more details explanation on the differences between BV and BsV.

**A:** Thank you for your suggestion. We have incorporated detailed explanations clarifying the differences between BV and BsV in several sections of our manuscript, including "Context," "Participant Information and Experimental Setup," and "Stimulus Interface and Encoding". Your feedback has been invaluable and has significantly improved the quality of our paper.

11. The last sentence on light intensity above Figure 2 is unclear. Please further explain.

**A:** Thank you very much for your suggestion. We have rewritten this sentence to ensure clearer expression.

12. Why not have CA cells be 1*1 pixel as well?

**A:** From the perspective of writing the paper, the reason for using three pixels is that previous studies have adopted this approach. However, on a personal note, I can tell you that we have tried single-pixel stimulation, but the results were not as effective as the three-pixel system. Since we haven't conducted a systematic scientific comparison, I cannot provide a definitive answer. However, I suspect that a single pixel may not be spatially distinguishable to the human eye, thus the stimulus sequence might be perceived as an envelope signal rather than a combination of two frequencies.

13. How were the frequencies and phases selected? How were the stimuli frequency and phase layouts determined?

**A:** Thank you for your question. We directly used the frequency-phase encoding and spatial arrangements from previous research. We have emphasized this point and added the relevant references in the "Stimulus interface and encoding" section.

14. In Figure 3 caption, remove BV on the third line.

A: Thank you very much for your reminder. We have corrected this error in the text.

15. In SNR definitions, delta f definition is not clear.

A: Thank you very much for your reminder. The unclear definition was actually due to a mistake in the formula 2. We have corrected this error and hope this resolves your concern.

16. At the bottom of page 6/15, you mentioned that the data length is too short for plotting the spectra, a potential solution is to zero-pad your data to make it longer for better frequency resolution.

A: Thank you very much for your suggestion. Actually, zero-padding acts as a form of smooth interpolation for the spectrum and does not provide new information. The resolution achieved through zero-padding is only of computational significance, not of actual physical significance. The true improvement in spectral resolution can only be achieved by increasing the effective sample length. For a detailed explanation on this, you can refer to Steven W. Smith's "Digital Signal Processing: A Practical Guide for Engineers and Scientists."

17. The CCA spectra should be further explained with more details.

A: Thank you very much for your suggestion. We have added more details about the CCA spectra. However, it should be noted that this method is currently not widely used, and its underlying principles have not been fully analyzed. Since this is not the main focus of our paper, we have only provided a brief introduction to the method. We are in the process of writing another paper that will systematically summarize and analyze this method.

18. 'x(t)' in equation 3 was not explained.

A: Thank you very much for pointing that out. We have added the relevant information at the corresponding position.

Data validation and quality control:

1. On Figures 4 and 5, suggest labelling the linear combinations and harmonics on the graphs. E.g., 19.6Hz = f1+f2, as how you did with 11.6 Hz.

A: Thank you very much for your suggestion. We have modified the figures accordingly to label the linear combinations and harmonics as you recommended.

2. Figure 4: it would be great if you have participants who did both paradigms and if you can show their data for a more direct comparison.

**A:** Thank you for your suggestion. Unfortunately, after careful review, we found that there were no participants who completed both the CA and BV paradigms.

3. Figure 4: could you please explain why in the BV paradigm topography plots, why all of them showed strong activity on only one side?

**A:** This is indeed an interesting observation. I personally believe this is due to a stronger response in the right visual

field compared to the left visual field. The underlying cause could be related to habitual eye use or individual differences in neural development. However, this is quite normal. In my personal experience, many people exhibit a lateralized SSVEP response area, and those with responses centered precisely at Oz are actually in the minority.

4. Figure 4 caption has a typo: 'CV' should be changed to 'CA'.

**A:** Thank you for pointing this out. We have corrected this error.

5. Figure 5: please consider having the topography of the same frequency vertically aligned and have the two waveforms plotted on the same figure to help visualise a direct comparison.

**A:** Thank you very much for your suggestion. However, I personally believe that this method might not be very intuitive. The overlap between the two waveforms is quite high, and if plotted together, readers might find it difficult to distinguish between them. I have attached an example figure for your reference to illustrate the effect.

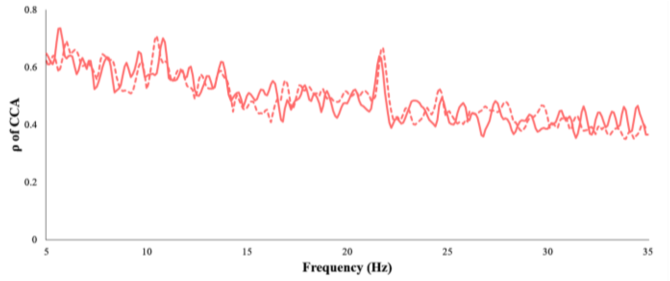


Additionally, if we follow your previous suggestion to label the chart as "f1+f2" and overlay the two graphs, the different f1 and f2 for the two targets might confuse readers. For example, regarding 10.9Hz, one graph would show f1 and the other f2. Should I label it as f1 or f2? Or should I annotate it as f1 for Target A and f2 for Target B in the figure? Either way, I feel it would make the readers more confused. I believe the current arrangement is clearer. However, I still greatly appreciate your feedback as it is very important for improving the quality of our paper.

6. Third line in SNR ratio distribution analysis: the normal distribution does not support your claim "attesting to the robustness and reliability of the dataset". To show this, you will need to compare to other studies. For example reference 20 if you like.

**A:** Thank you very much for your comments. We realize that our initial statement might be confusing. We have now added a comparison with previous seminal datasets to substantiate our point.

7. Average SNR: (second sentence) in narrowband SNR, BV>BsV according to your plot. Suggest running statistical tests on these comparisons. It looks like there might not be any significant differences between the different paradigms.

**A:** Thank you very much for your suggestion. We have incorporated the results of the statistical tests into Figure 7. Although the mean values appear to show only slight differences, the differences are actually statistically significant.

8. Figure 7: please update them to have the same scale. The differences are actually quite small, but with the zoomed in view, it may mislead reader to have a false perception.

**A:** Thank you very much for your suggestion. We apologize for any confusion caused by not presenting the statistical test results earlier. In fact, these differences are significant, and the zoomed-in view is necessary to clearly demonstrate these distinctions.

9. My rough thoughts on training-free classification on BsV: since differences were observed from the topography, perhaps applying different spatial filters may help in decoding BsV with a training-free method.

**A:** Your idea is quite interesting. However, the challenge lies in the fact that each individual's topographical map is different, with significant individual variability. It is challenging to identify a universal prior spatial filter that works for everyone. Nevertheless, I also believe that exploring methods to achieve training-free BsV classification is a valuable research direction.

10. Classification results with training: suggest also perform statistical tests to support your claims such as "The results demonstrate that the performance metrics of correctness and ITR for both the CA and BV paradigms are closely matched".

**A:** Thank you very much for your suggestion. We have now included statistical test results to support our claims.

Supplementary Tables:

1. Supplementary Table 1 can be combined with Table 1 in the paper.

**A:** We have combined Supplementary Table 1 with Table 1 in the main paper, as suggested. This consolidation should improve the clarity and accessibility of the data presented.

2. Supplementary Tables 2 and 3: make the units clear in your tables. Also show standard errors.

**A:** We have updated Supplementary Tables 2 and 3 to ensure that all units are clearly indicated. Additionally, we have included the standard errors for the measurements presented in these tables. These changes should enhance the comprehensibility and accuracy of the data.

IRB approval

Click here to access/download
**Supplementary Material**
Institutional_Review_Board_approval.pdf

Click here to access/download
**Supplementary Material**
Institutional_Review_Board_approval_Translate_to_English.pdf

Click here to access/download
**Supplementary Material**
Medical_Ethics_Informed_Consent_Form.pdf

Click here to access/download
**Supplementary Material**
Medical_Ethics_Informed_Consent_Form_Translate_to_English.pdf

Informed consent doc

Click here to access/download
**Supplementary Material**
Informed_Consent_Document.pdf

Informed consent doc English

Click here to access/download
**Supplementary Material**
Informed_Consent_Document_Translate_to_English.pdf

Click here to access/download
**Supplementary Material**
Supplementary materials.docx

Graphical Abstract

Click here to access/download;Graphical Abstract;Graphical Abstract.tif

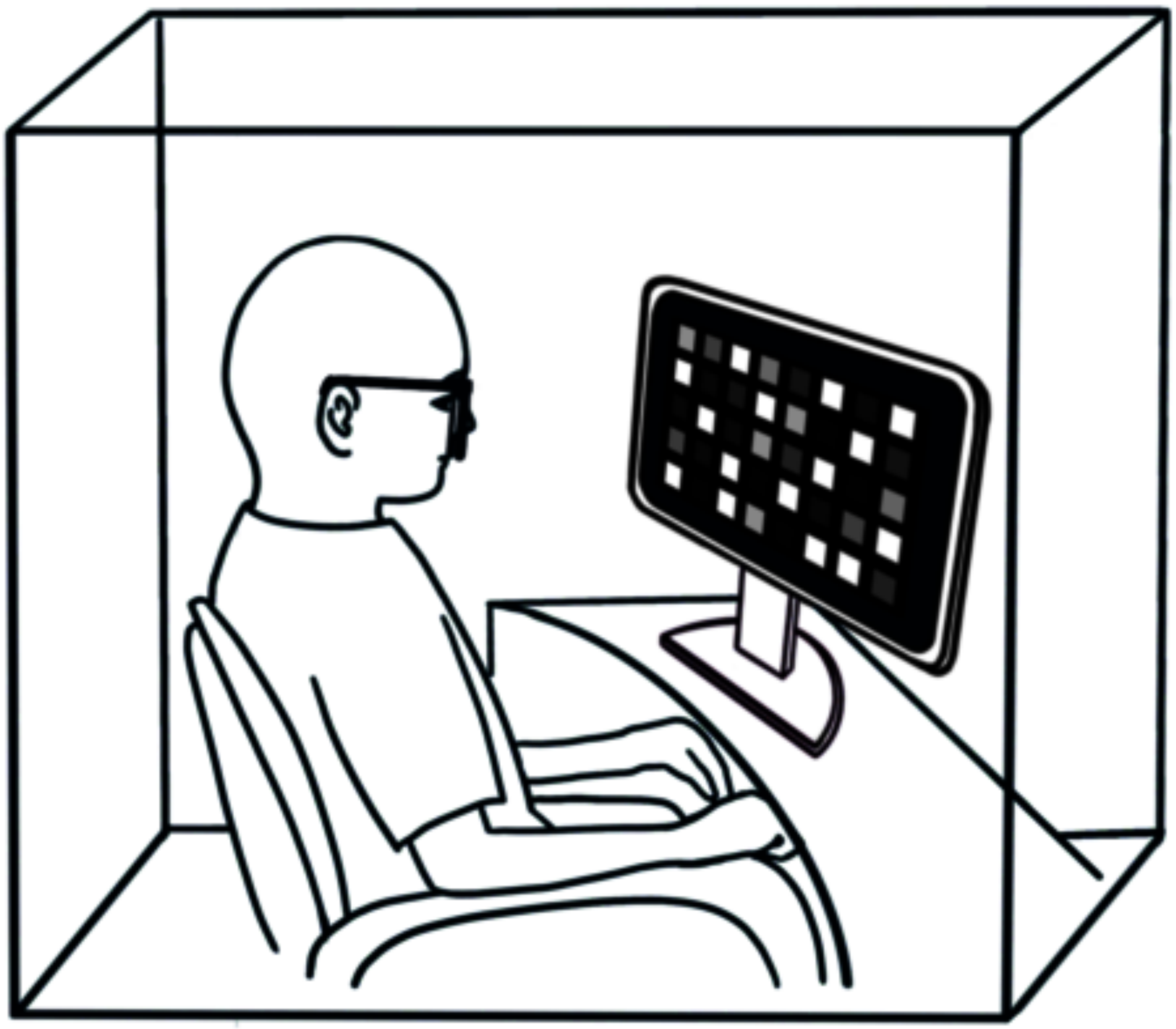

Supplement: giae041_GIGA-D-24-00125_Revision_1 [file giae041_giga-d-24-00125_revision_1.pdf]
