# Supplementary material for: Dual-Alpha: a large EEG study for dual-frequency SSVEP brain–computer interface
Source: Gigascience. 2024 Aug 7;13:giae041. doi: 10.1093/gigascience/giae041 (PMC11304967; doi:10.1093/gigascience/giae041)
Supplement: giae041_Supplemental_File [file giae041_supplemental_file.docx]

## Supplementary materials

**Supplementary Table 1.** Duplicate participants' information statistics

| **information** | **No. in CA** | **No. in BV** | **No. in BsV** |
| --- | --- | --- | --- |
| Gender: Male; Age: 23; Dominant eye: Right | Non-Participation | No.8 | No.14 |
| Gender: Female; Age: 24; Dominant eye: Right | Non-Participation | No.7 | No.19 |
| Gender: Male; Age: 26; Dominant eye: Right | Non-Participation | No.20 | No.29 |
| Gender: Female; Age: 23; Dominant eye: Left | Non-Participation | No.26 | No.30 |
| Gender: Female; Age: 22; Dominant eye: Right | No.35 | Non-Participation | No.35 |

**Supplementary Table 2.** Classification results of FBDCCA

| **Classification Accuracy of Binocular Vision (%)** | | | | | | | | | | | |
| --- | --- | --- | --- | --- | --- | --- | --- | --- | --- | --- | --- |
| **Name\Time** | **0** | **0.2** | **0.4** | **0.6** | **0.8** | **1** | **1.2** | **1.4** | **1.6** | **1.8** | **2** |
| Subject1 | 0.00 | 3.00 | 9.00 | 15.00 | 29.00 | 59.00 | 77.50 | 94.00 | 96.50 | 98.50 | 98.50 |
| Subject2 | 0.00 | 3.50 | 1.50 | 11.00 | 21.00 | 27.50 | 43.50 | 53.50 | 60.50 | 66.00 | 72.00 |
| Subject3 | 0.00 | 3.50 | 6.50 | 9.00 | 19.00 | 24.00 | 32.00 | 48.00 | 56.00 | 66.00 | 69.00 |
| Subject4 | 0.00 | 2.50 | 18.50 | 30.50 | 56.50 | 73.50 | 82.50 | 93.50 | 96.00 | 98.00 | 99.00 |
| Subject5 | 0.00 | 3.00 | 11.50 | 17.00 | 33.50 | 48.50 | 63.00 | 69.00 | 78.00 | 79.00 | 81.50 |
| Subject6 | 0.00 | 3.00 | 3.50 | 4.00 | 3.00 | 4.50 | 2.50 | 3.50 | 4.00 | 4.00 | 3.00 |
| Subject7 | 0.00 | 3.50 | 5.00 | 2.50 | 5.50 | 6.50 | 7.00 | 8.00 | 12.00 | 12.50 | 17.00 |
| Subject8 | 0.00 | 4.00 | 5.50 | 4.50 | 8.50 | 9.50 | 15.00 | 15.00 | 16.50 | 17.50 | 21.00 |
| Subject9 | 0.00 | 2.50 | 5.50 | 11.00 | 13.50 | 16.00 | 16.00 | 22.00 | 34.50 | 30.00 | 37.50 |
| Subject10 | 0.00 | 3.00 | 5.00 | 8.50 | 9.00 | 9.50 | 9.50 | 12.00 | 14.00 | 19.00 | 22.50 |
| Subject11 | 0.00 | 2.00 | 4.00 | 11.00 | 10.00 | 16.50 | 19.00 | 18.50 | 24.50 | 30.00 | 32.50 |
| Subject12 | 0.00 | 3.50 | 4.50 | 4.50 | 4.50 | 7.50 | 9.50 | 10.00 | 10.50 | 11.00 | 12.50 |
| Subject13 | 0.00 | 4.00 | 10.00 | 18.50 | 31.50 | 51.50 | 71.00 | 86.50 | 88.50 | 92.00 | 93.00 |
| Subject14 | 0.00 | 1.50 | 7.00 | 12.00 | 24.00 | 42.50 | 58.00 | 72.50 | 80.50 | 83.00 | 86.00 |
| Subject15 | 0.00 | 4.50 | 6.00 | 17.00 | 33.50 | 45.00 | 51.50 | 63.00 | 72.50 | 77.50 | 83.50 |
| Subject16 | 0.00 | 3.50 | 1.50 | 7.50 | 13.00 | 24.00 | 35.50 | 49.50 | 52.50 | 63.00 | 66.00 |
| Subject17 | 0.00 | 4.00 | 12.50 | 34.50 | 54.00 | 73.50 | 85.50 | 93.00 | 94.00 | 95.50 | 93.00 |
| Subject18 | 0.00 | 1.50 | 3.00 | 18.50 | 41.00 | 64.50 | 85.00 | 92.50 | 98.50 | 98.50 | 99.50 |
| Subject19 | 0.00 | 3.00 | 3.00 | 13.00 | 24.50 | 40.50 | 59.50 | 73.00 | 81.50 | 88.50 | 90.00 |
| Subject20 | 0.00 | 2.50 | 7.00 | 19.50 | 40.50 | 48.50 | 60.50 | 70.00 | 81.00 | 86.50 | 90.50 |
| Subject21 | 0.00 | 5.00 | 1.50 | 5.50 | 12.50 | 17.00 | 26.00 | 41.00 | 58.00 | 67.00 | 76.50 |
| Subject22 | 0.00 | 2.00 | 6.00 | 11.00 | 25.50 | 34.00 | 52.00 | 73.00 | 80.50 | 88.50 | 88.00 |
| Subject23 | 0.00 | 4.00 | 6.50 | 8.00 | 15.50 | 24.00 | 28.50 | 40.50 | 48.00 | 52.00 | 57.50 |
| Subject24 | 0.00 | 1.50 | 5.00 | 9.50 | 14.00 | 22.50 | 38.50 | 51.00 | 61.00 | 73.50 | 78.00 |
| Subject25 | 0.00 | 3.00 | 6.50 | 12.00 | 23.50 | 39.00 | 60.00 | 72.50 | 80.00 | 85.00 | 91.00 |
| Subject26 | 0.00 | 1.50 | 4.50 | 11.00 | 23.00 | 35.50 | 46.50 | 67.00 | 76.50 | 79.00 | 83.50 |
| Subject27 | 0.00 | 1.50 | 5.00 | 6.00 | 5.50 | 8.00 | 12.50 | 16.00 | 18.00 | 20.50 | 19.50 |
| Subject28 | 0.00 | 3.00 | 3.00 | 6.00 | 11.00 | 18.00 | 24.50 | 28.50 | 40.50 | 49.50 | 54.00 |
| Subject29 | 0.00 | 3.00 | 4.00 | 8.00 | 8.50 | 12.00 | 14.00 | 16.50 | 23.50 | 29.50 | 35.00 |
| Subject30 | 0.00 | 3.00 | 3.50 | 8.50 | 10.00 | 15.50 | 19.00 | 28.00 | 35.50 | 41.50 | 50.00 |
| Subject31 | 0.00 | 5.00 | 7.00 | 13.00 | 24.50 | 37.00 | 52.00 | 62.00 | 69.00 | 74.00 | 74.50 |
| Subject32 | 0.00 | 2.50 | 3.00 | 7.50 | 12.50 | 25.00 | 39.00 | 54.00 | 65.50 | 66.50 | 73.00 |
| Subject33 | 0.00 | 4.00 | 5.50 | 7.00 | 8.50 | 14.50 | 13.50 | 16.50 | 19.50 | 25.50 | 24.50 |
| Subject34 | 0.00 | 4.50 | 13.00 | 24.00 | 41.50 | 61.50 | 81.50 | 89.00 | 92.50 | 93.50 | 96.00 |
| Subject35 | 0.00 | 2.50 | 4.50 | 10.00 | 16.50 | 28.00 | 38.50 | 41.50 | 52.50 | 60.00 | 65.50 |
| Average  ±Standard Deviation | 0.00  ±0 | 3.07  ±0.99 | 5.96  ±3.58 | 11.89  ±7.11 | 20.79  ±13.83 | 30.97  ±19.77 | 40.84  ±25.28 | 49.83  ±28.88 | 56.36  ±29.45 | 60.61  ±29.77 | 63.83  ±29.62 |
| **Classification Accuracy of Checkerboard Arrangement (%)** | | | | | | | | | | | |
| **Name\Time** | **0** | **0.2** | **0.4** | **0.6** | **0.8** | **1** | **1.2** | **1.4** | **1.6** | **1.8** | **2** |
| Subject1 | 0.00 | 1.50 | 2.00 | 1.50 | 3.00 | 2.50 | 3.50 | 4.50 | 4.50 | 5.50 | 5.00 |
| Subject2 | 0.00 | 2.00 | 4.50 | 9.00 | 22.00 | 37.50 | 64.50 | 78.00 | 85.50 | 86.00 | 88.00 |
| Subject3 | 0.00 | 3.50 | 6.50 | 7.50 | 15.00 | 21.00 | 34.50 | 51.00 | 53.50 | 57.50 | 64.00 |
| Subject4 | 0.00 | 3.50 | 8.50 | 8.50 | 8.50 | 5.50 | 10.50 | 13.50 | 18.00 | 21.00 | 20.00 |
| Subject5 | 0.00 | 3.50 | 6.00 | 4.00 | 4.00 | 4.50 | 7.00 | 14.50 | 19.00 | 23.50 | 27.00 |
| Subject6 | 0.00 | 3.00 | 5.00 | 11.00 | 11.50 | 14.50 | 21.50 | 35.00 | 42.50 | 48.50 | 49.50 |
| Subject7 | 0.00 | 4.50 | 3.00 | 6.00 | 7.50 | 10.50 | 22.00 | 39.50 | 47.00 | 56.00 | 63.50 |
| Subject8 | 0.00 | 2.50 | 5.50 | 7.50 | 11.50 | 21.50 | 40.00 | 49.50 | 55.00 | 63.00 | 67.00 |
| Subject9 | 0.00 | 3.50 | 8.00 | 6.50 | 6.50 | 10.50 | 17.00 | 36.50 | 44.00 | 49.00 | 49.50 |
| Subject10 | 0.00 | 2.00 | 3.00 | 9.50 | 10.00 | 12.50 | 18.00 | 27.00 | 32.00 | 30.50 | 29.00 |
| Subject11 | 0.00 | 4.50 | 8.00 | 13.00 | 19.00 | 34.50 | 60.00 | 79.50 | 84.00 | 87.00 | 86.50 |
| Subject12 | 0.00 | 1.50 | 3.50 | 3.00 | 3.50 | 3.00 | 3.00 | 4.00 | 4.50 | 5.00 | 2.00 |
| Subject13 | 0.00 | 2.50 | 4.00 | 8.50 | 10.00 | 12.50 | 20.00 | 31.50 | 34.00 | 44.50 | 46.50 |
| Subject14 | 0.00 | 2.50 | 4.00 | 3.00 | 4.50 | 5.50 | 8.50 | 7.50 | 11.00 | 16.00 | 17.50 |
| Subject15 | 0.00 | 3.00 | 2.50 | 4.00 | 5.50 | 5.00 | 7.00 | 11.00 | 15.50 | 17.00 | 23.50 |
| Subject16 | 0.00 | 4.50 | 6.00 | 7.50 | 9.00 | 9.00 | 9.00 | 15.50 | 13.50 | 16.00 | 18.00 |
| Subject17 | 0.00 | 3.50 | 4.00 | 6.50 | 5.00 | 6.00 | 5.50 | 6.00 | 5.50 | 9.00 | 10.00 |
| Subject18 | 0.00 | 2.50 | 5.00 | 6.00 | 5.50 | 9.50 | 7.00 | 8.50 | 13.00 | 15.00 | 20.50 |
| Subject19 | 0.00 | 2.50 | 3.50 | 4.50 | 6.00 | 11.00 | 16.00 | 31.50 | 33.50 | 46.50 | 45.00 |
| Subject20 | 0.00 | 2.50 | 1.50 | 3.00 | 2.50 | 0.50 | 1.50 | 3.00 | 2.00 | 4.00 | 3.50 |
| Subject21 | 0.00 | 2.50 | 7.50 | 7.00 | 13.50 | 16.00 | 21.00 | 34.50 | 38.50 | 42.00 | 44.00 |
| Subject22 | 0.00 | 3.00 | 3.50 | 4.00 | 9.00 | 8.00 | 11.50 | 22.00 | 25.50 | 34.00 | 36.00 |
| Subject23 | 0.00 | 3.00 | 1.50 | 3.50 | 3.00 | 4.00 | 3.50 | 4.50 | 6.00 | 3.50 | 2.50 |
| Subject24 | 0.00 | 2.50 | 11.50 | 9.00 | 12.00 | 15.00 | 23.00 | 27.50 | 30.00 | 36.00 | 35.00 |
| Subject25 | 0.00 | 6.50 | 4.00 | 4.50 | 2.50 | 4.50 | 3.00 | 5.00 | 6.00 | 8.50 | 9.00 |
| Subject26 | 0.00 | 2.50 | 2.50 | 2.50 | 2.50 | 2.50 | 4.00 | 4.50 | 6.00 | 5.00 | 5.00 |
| Subject27 | 0.00 | 3.50 | 3.50 | 5.00 | 4.00 | 4.00 | 3.50 | 3.00 | 2.50 | 3.00 | 2.50 |
| Subject28 | 0.00 | 2.50 | 1.50 | 4.00 | 2.50 | 2.00 | 3.50 | 2.00 | 4.50 | 4.00 | 3.50 |
| Subject29 | 0.00 | 3.50 | 2.50 | 2.00 | 3.00 | 2.00 | 3.50 | 4.00 | 4.50 | 4.50 | 3.50 |
| Subject30 | 0.00 | 2.00 | 2.50 | 2.00 | 2.00 | 2.50 | 2.00 | 2.00 | 1.50 | 3.00 | 3.00 |
| Subject31 | 0.00 | 2.50 | 1.00 | 3.00 | 5.50 | 3.00 | 2.50 | 2.50 | 2.50 | 3.50 | 3.00 |
| Subject32 | 0.00 | 2.50 | 3.00 | 1.00 | 2.00 | 4.00 | 2.50 | 4.00 | 1.50 | 2.00 | 3.00 |
| Subject33 | 0.00 | 3.50 | 3.00 | 0.50 | 2.00 | 2.00 | 2.50 | 4.00 | 3.00 | 3.00 | 4.00 |
| Subject34 | 0.00 | 2.50 | 4.50 | 7.00 | 3.00 | 4.00 | 3.50 | 3.50 | 2.00 | 1.50 | 2.50 |
| Subject35 | 0.00 | 2.50 | 3.00 | 9.50 | 18.50 | 32.00 | 48.50 | 65.00 | 66.00 | 65.00 | 64.00 |
| Average  ±Standard Deviation | 0.00  ±0 | 2.97  ±0.96 | 4.26  ±2.33 | 5.56  ±3.04 | 7.29  ±5.33 | 9.79  ±9.44 | 14.69  ±16.49 | 21.00  ±22.11 | 23.36  ±23.92 | 26.26  ±25.37 | 27.31  ±26.17 |

**Supplementary Table 3.** Classification results of TRCA

| **Classification Accuracy of Binocular Vision (%)** | | | | | | | | | | | |
| --- | --- | --- | --- | --- | --- | --- | --- | --- | --- | --- | --- |
| **Name\Time** | **0** | **0.2** | **0.4** | **0.6** | **0.8** | **1** | **1.2** | **1.4** | **1.6** | **1.8** | **2** |
| Subject1 | 0.00 | 76.00 | 92.50 | 93.50 | 94.50 | 95.50 | 95.50 | 95.50 | 96.00 | 95.50 | 96.00 |
| Subject2 | 0.00 | 40.00 | 73.50 | 83.50 | 86.00 | 85.50 | 87.00 | 87.50 | 91.50 | 92.50 | 90.50 |
| Subject3 | 0.00 | 63.00 | 86.50 | 90.50 | 96.00 | 98.50 | 98.50 | 99.50 | 99.50 | 99.50 | 99.50 |
| Subject4 | 0.00 | 51.00 | 88.00 | 91.00 | 97.00 | 98.00 | 99.00 | 99.50 | 99.50 | 99.50 | 99.50 |
| Subject5 | 0.00 | 75.50 | 92.50 | 95.50 | 96.50 | 98.00 | 99.50 | 99.50 | 99.00 | 100.00 | 100.00 |
| Subject6 | 0.00 | 10.50 | 9.50 | 8.00 | 6.50 | 7.00 | 4.00 | 7.50 | 8.50 | 5.00 | 6.50 |
| Subject7 | 0.00 | 42.50 | 58.50 | 51.00 | 60.00 | 55.00 | 66.00 | 73.00 | 75.50 | 79.50 | 83.00 |
| Subject8 | 0.00 | 19.00 | 40.00 | 49.00 | 47.50 | 54.00 | 59.00 | 65.50 | 67.50 | 67.50 | 67.00 |
| Subject9 | 0.00 | 16.00 | 30.00 | 48.50 | 54.50 | 57.50 | 61.00 | 65.00 | 64.00 | 67.00 | 68.00 |
| Subject10 | 0.00 | 25.50 | 45.00 | 50.50 | 56.00 | 64.00 | 66.00 | 70.50 | 76.00 | 80.50 | 78.50 |
| Subject11 | 0.00 | 41.00 | 71.50 | 70.50 | 74.00 | 74.50 | 77.00 | 82.00 | 84.50 | 87.00 | 90.00 |
| Subject12 | 0.00 | 30.50 | 60.00 | 71.50 | 76.50 | 80.00 | 79.00 | 86.50 | 91.00 | 91.50 | 94.50 |
| Subject13 | 0.00 | 78.50 | 86.00 | 86.00 | 87.50 | 87.50 | 87.00 | 87.50 | 87.50 | 89.00 | 88.00 |
| Subject14 | 0.00 | 30.50 | 67.50 | 88.50 | 96.50 | 98.50 | 99.00 | 99.50 | 100.00 | 99.50 | 99.50 |
| Subject15 | 0.00 | 46.00 | 70.00 | 81.50 | 86.00 | 84.00 | 85.00 | 85.50 | 85.50 | 86.00 | 86.00 |
| Subject16 | 0.00 | 16.50 | 43.50 | 57.00 | 73.50 | 77.50 | 80.00 | 82.50 | 86.00 | 86.00 | 87.50 |
| Subject17 | 0.00 | 60.00 | 87.00 | 88.00 | 92.00 | 93.50 | 93.50 | 94.00 | 94.50 | 94.50 | 95.50 |
| Subject18 | 0.00 | 77.50 | 88.00 | 93.00 | 97.00 | 99.00 | 99.00 | 99.50 | 99.00 | 99.00 | 99.50 |
| Subject19 | 0.00 | 32.00 | 56.00 | 70.00 | 84.50 | 88.50 | 94.00 | 95.50 | 97.50 | 98.50 | 98.50 |
| Subject20 | 0.00 | 44.50 | 78.00 | 89.00 | 93.00 | 96.00 | 97.50 | 97.50 | 97.50 | 97.00 | 97.50 |
| Subject21 | 0.00 | 37.00 | 66.50 | 81.00 | 91.50 | 97.00 | 98.50 | 99.50 | 98.50 | 99.50 | 99.50 |
| Subject22 | 0.00 | 50.50 | 79.50 | 90.50 | 94.50 | 96.00 | 95.00 | 94.00 | 95.00 | 95.50 | 97.00 |
| Subject23 | 0.00 | 21.50 | 37.00 | 45.50 | 53.00 | 54.00 | 58.50 | 64.50 | 65.50 | 65.50 | 68.50 |
| Subject24 | 0.00 | 57.00 | 85.00 | 95.00 | 98.00 | 98.00 | 99.00 | 98.00 | 99.50 | 100.00 | 100.00 |
| Subject25 | 0.00 | 30.50 | 65.50 | 85.00 | 94.50 | 95.50 | 98.00 | 98.50 | 98.50 | 98.00 | 99.00 |
| Subject26 | 0.00 | 57.00 | 85.00 | 94.00 | 96.00 | 98.50 | 99.00 | 99.00 | 98.00 | 98.00 | 98.00 |
| Subject27 | 0.00 | 23.00 | 47.50 | 58.50 | 66.00 | 70.00 | 76.50 | 80.00 | 82.00 | 86.00 | 88.00 |
| Subject28 | 0.00 | 32.00 | 67.50 | 83.00 | 86.00 | 91.50 | 91.00 | 92.00 | 92.50 | 95.00 | 95.50 |
| Subject29 | 0.00 | 11.00 | 27.00 | 35.00 | 47.00 | 50.50 | 62.50 | 67.00 | 72.50 | 76.50 | 80.50 |
| Subject30 | 0.00 | 50.00 | 66.00 | 77.50 | 81.50 | 85.50 | 86.00 | 88.00 | 87.50 | 88.00 | 88.00 |
| Subject31 | 0.00 | 48.00 | 79.50 | 89.50 | 94.50 | 95.00 | 98.00 | 98.00 | 98.50 | 98.00 | 98.00 |
| Subject32 | 0.00 | 36.50 | 65.00 | 86.50 | 93.00 | 96.00 | 98.50 | 99.00 | 99.50 | 99.50 | 99.50 |
| Subject33 | 0.00 | 16.00 | 24.00 | 31.00 | 42.00 | 46.00 | 48.50 | 51.50 | 56.00 | 56.00 | 58.00 |
| Subject34 | 0.00 | 61.00 | 80.00 | 83.50 | 85.50 | 85.50 | 88.50 | 89.00 | 89.50 | 90.50 | 91.00 |
| Subject35 | 0.00 | 34.00 | 73.50 | 86.00 | 93.50 | 96.00 | 97.00 | 99.00 | 99.50 | 100.00 | 100.00 |
| Average  ±Standard Deviation | 0.00  ±0 | 41.17  ±19.52 | 64.91  ±21.66 | 73.64  ±21.79 | 79.19  ±21.30 | 81.34  ±21.07 | 83.46  ±20.28 | 85.43  ±18.82 | 86.64  ±18.18 | 87.44  ±18.39 | 88.16  ±17.99 |
| **Classification Accuracy of Checkerboard Arrangement (%)** | | | | | | | | | | | |
| **Name\Time** | **0** | **0.2** | **0.4** | **0.6** | **0.8** | **1** | **1.2** | **1.4** | **1.6** | **1.8** | **2** |
| Subject1 | 0.00 | 14.00 | 24.00 | 35.00 | 47.50 | 62.00 | 72.50 | 80.50 | 81.00 | 87.00 | 89.00 |
| Subject2 | 0.00 | 59.00 | 89.50 | 95.50 | 96.50 | 97.50 | 99.00 | 99.00 | 99.00 | 99.00 | 99.00 |
| Subject3 | 0.00 | 34.00 | 61.50 | 76.00 | 82.50 | 85.00 | 88.00 | 87.50 | 90.00 | 91.50 | 91.50 |
| Subject4 | 0.00 | 48.50 | 76.50 | 89.50 | 93.00 | 94.50 | 97.00 | 97.00 | 97.00 | 97.00 | 98.00 |
| Subject5 | 0.00 | 48.50 | 76.00 | 88.50 | 93.00 | 94.50 | 96.00 | 98.00 | 98.50 | 99.50 | 99.00 |
| Subject6 | 0.00 | 80.50 | 97.00 | 97.00 | 97.50 | 97.50 | 99.50 | 100.00 | 100.00 | 100.00 | 100.00 |
| Subject7 | 0.00 | 71.00 | 88.50 | 95.00 | 97.00 | 98.50 | 98.50 | 99.00 | 99.00 | 99.00 | 99.00 |
| Subject8 | 0.00 | 28.50 | 53.00 | 70.00 | 80.50 | 84.50 | 88.00 | 90.00 | 92.00 | 91.50 | 90.50 |
| Subject9 | 0.00 | 70.00 | 91.50 | 96.50 | 96.50 | 98.50 | 97.50 | 98.50 | 98.50 | 98.00 | 98.50 |
| Subject10 | 0.00 | 21.50 | 46.50 | 63.50 | 70.00 | 78.50 | 81.00 | 86.50 | 87.00 | 90.50 | 91.50 |
| Subject11 | 0.00 | 72.50 | 98.00 | 100.00 | 99.50 | 100.00 | 100.00 | 100.00 | 100.00 | 100.00 | 100.00 |
| Subject12 | 0.00 | 33.00 | 66.00 | 80.00 | 86.00 | 91.50 | 94.50 | 96.00 | 99.00 | 100.00 | 100.00 |
| Subject13 | 0.00 | 26.50 | 53.50 | 71.50 | 76.50 | 83.50 | 88.00 | 89.50 | 89.00 | 89.00 | 92.50 |
| Subject14 | 0.00 | 52.00 | 68.50 | 81.50 | 87.00 | 88.50 | 92.00 | 93.50 | 95.00 | 95.50 | 95.50 |
| Subject15 | 0.00 | 49.00 | 77.00 | 80.00 | 84.00 | 85.00 | 85.00 | 87.50 | 87.50 | 88.50 | 88.50 |
| Subject16 | 0.00 | 70.50 | 87.50 | 90.50 | 90.50 | 91.00 | 92.50 | 93.50 | 95.00 | 95.00 | 95.50 |
| Subject17 | 0.00 | 86.50 | 95.00 | 95.50 | 95.50 | 95.00 | 95.00 | 95.00 | 95.00 | 95.50 | 95.00 |
| Subject18 | 0.00 | 88.00 | 92.00 | 93.00 | 93.50 | 95.00 | 95.00 | 95.00 | 95.50 | 95.50 | 95.00 |
| Subject19 | 0.00 | 40.00 | 71.50 | 81.50 | 81.00 | 88.00 | 91.00 | 93.00 | 94.50 | 94.00 | 94.50 |
| Subject20 | 0.00 | 31.50 | 60.00 | 74.50 | 77.00 | 87.00 | 86.50 | 90.00 | 90.00 | 91.00 | 92.50 |
| Subject21 | 0.00 | 85.00 | 95.50 | 94.50 | 95.00 | 95.00 | 95.00 | 96.00 | 96.50 | 95.50 | 96.00 |
| Subject22 | 0.00 | 85.00 | 94.00 | 95.00 | 95.00 | 95.50 | 95.50 | 95.50 | 96.00 | 96.00 | 96.00 |
| Subject23 | 0.00 | 56.00 | 86.00 | 94.50 | 99.50 | 99.00 | 99.50 | 99.50 | 99.50 | 99.50 | 100.00 |
| Subject24 | 0.00 | 79.00 | 90.00 | 93.00 | 91.00 | 91.50 | 91.50 | 94.50 | 93.50 | 93.00 | 94.00 |
| Subject25 | 0.00 | 57.50 | 81.50 | 87.50 | 89.50 | 90.50 | 94.00 | 94.50 | 94.00 | 95.50 | 95.00 |
| Subject26 | 0.00 | 14.00 | 30.00 | 46.50 | 57.50 | 61.50 | 69.00 | 72.50 | 76.50 | 81.00 | 84.00 |
| Subject27 | 0.00 | 74.00 | 87.00 | 94.50 | 98.50 | 99.00 | 99.00 | 99.50 | 99.50 | 100.00 | 100.00 |
| Subject28 | 0.00 | 45.00 | 75.50 | 86.00 | 89.50 | 97.00 | 99.50 | 99.00 | 99.50 | 100.00 | 100.00 |
| Subject29 | 0.00 | 23.00 | 44.00 | 56.00 | 57.50 | 64.50 | 67.00 | 74.00 | 76.00 | 83.00 | 83.50 |
| Subject30 | 0.00 | 31.50 | 52.50 | 59.50 | 72.50 | 70.00 | 77.50 | 78.00 | 81.50 | 83.00 | 87.50 |
| Subject31 | 0.00 | 70.50 | 93.50 | 98.00 | 99.50 | 99.50 | 100.00 | 100.00 | 100.00 | 100.00 | 100.00 |
| Subject32 | 0.00 | 22.00 | 28.50 | 41.50 | 42.00 | 42.50 | 49.00 | 54.50 | 54.50 | 58.50 | 62.50 |
| Subject33 | 0.00 | 14.50 | 34.00 | 51.00 | 65.50 | 78.00 | 80.00 | 86.00 | 88.50 | 91.50 | 93.50 |
| Subject34 | 0.00 | 49.50 | 67.50 | 70.50 | 75.50 | 84.00 | 89.50 | 93.00 | 94.50 | 96.50 | 96.50 |
| Subject35 | 0.00 | 37.00 | 69.50 | 77.50 | 87.00 | 89.50 | 93.00 | 94.50 | 96.50 | 96.50 | 96.50 |
| Average  ±Standard Deviation | 0.00  ±0 | 50.53  ±23.42 | 71.49  ±21.75 | 80.00  ±17.66 | 83.99  ±15.11 | 87.21  ±13.16 | 89.59  ±11.31 | 91.43  ±9.69 | 92.26  ±9.30 | 93.33  ±8.01 | 94.00  ±7.11 |
| **Classification Accuracy of Binocular-swap vision (%)** | | | | | | | | | | | |
| **Name\Time** | **0** | **0.2** | **0.4** | **0.6** | **0.8** | **1** | **1.2** | **1.4** | **1.6** | **1.8** | **2** |
| Subject1 | 0.00 | 42.50 | 83.00 | 93.00 | 95.00 | 97.50 | 97.50 | 98.50 | 98.50 | 99.50 | 99.50 |
| Subject2 | 0.00 | 17.00 | 50.50 | 60.00 | 65.00 | 72.50 | 83.50 | 84.50 | 86.00 | 89.00 | 90.00 |
| Subject3 | 0.00 | 10.00 | 17.00 | 21.50 | 24.50 | 28.50 | 32.00 | 33.50 | 39.00 | 44.50 | 48.50 |
| Subject4 | 0.00 | 10.50 | 31.00 | 38.50 | 53.00 | 54.00 | 66.00 | 67.00 | 67.00 | 72.50 | 76.00 |
| Subject5 | 0.00 | 24.00 | 58.50 | 75.50 | 83.50 | 86.00 | 86.00 | 88.50 | 87.50 | 87.00 | 87.50 |
| Subject6 | 0.00 | 15.50 | 41.00 | 54.00 | 65.50 | 69.00 | 71.50 | 69.50 | 72.00 | 74.00 | 75.50 |
| Subject7 | 0.00 | 9.00 | 42.00 | 55.50 | 68.00 | 75.50 | 79.00 | 80.50 | 80.50 | 82.00 | 84.50 |
| Subject8 | 0.00 | 20.00 | 47.00 | 59.00 | 63.50 | 68.50 | 70.50 | 75.00 | 74.50 | 76.00 | 77.00 |
| Subject9 | 0.00 | 38.50 | 80.50 | 91.00 | 95.00 | 97.00 | 98.00 | 99.50 | 100.00 | 100.00 | 100.00 |
| Subject10 | 0.00 | 57.50 | 94.00 | 97.00 | 100.00 | 100.00 | 100.00 | 100.00 | 100.00 | 99.50 | 100.00 |
| Subject11 | 0.00 | 5.50 | 12.50 | 23.00 | 34.50 | 44.50 | 57.00 | 65.00 | 69.00 | 72.50 | 75.00 |
| Subject12 | 0.00 | 13.00 | 35.50 | 56.50 | 63.50 | 75.50 | 80.50 | 85.50 | 90.50 | 94.00 | 95.00 |
| Subject13 | 0.00 | 24.00 | 59.50 | 68.00 | 75.00 | 83.00 | 86.00 | 89.50 | 91.50 | 92.00 | 95.50 |
| Subject14 | 0.00 | 11.00 | 39.00 | 59.50 | 72.00 | 83.00 | 91.00 | 91.00 | 93.50 | 95.00 | 95.00 |
| Subject15 | 0.00 | 38.00 | 69.00 | 80.00 | 82.50 | 85.00 | 85.50 | 84.50 | 86.00 | 86.00 | 87.00 |
| Subject16 | 0.00 | 13.00 | 23.50 | 27.50 | 35.00 | 35.00 | 45.00 | 51.00 | 48.50 | 50.50 | 48.50 |
| Subject17 | 0.00 | 15.50 | 48.50 | 63.00 | 75.00 | 77.50 | 82.00 | 86.00 | 86.50 | 90.00 | 88.50 |
| Subject18 | 0.00 | 77.00 | 96.00 | 100.00 | 99.50 | 93.50 | 93.00 | 91.00 | 90.00 | 90.50 | 91.00 |
| Subject19 | 0.00 | 48.00 | 85.00 | 94.50 | 94.00 | 96.00 | 96.00 | 97.50 | 97.00 | 97.50 | 97.00 |
| Subject20 | 0.00 | 7.50 | 22.50 | 27.00 | 43.50 | 48.00 | 57.00 | 63.50 | 73.00 | 76.00 | 77.50 |
| Subject21 | 0.00 | 4.50 | 5.00 | 8.00 | 13.50 | 21.00 | 22.50 | 27.50 | 36.50 | 38.50 | 41.50 |
| Subject22 | 0.00 | 8.50 | 7.50 | 6.50 | 6.00 | 10.00 | 9.50 | 9.00 | 7.00 | 6.50 | 7.00 |
| Subject23 | 0.00 | 32.50 | 65.00 | 75.00 | 79.00 | 81.00 | 81.00 | 82.00 | 82.50 | 83.50 | 83.50 |
| Subject24 | 0.00 | 10.00 | 31.00 | 42.00 | 47.00 | 53.00 | 53.00 | 56.50 | 57.50 | 59.50 | 61.50 |
| Subject25 | 0.00 | 8.50 | 27.50 | 37.00 | 49.50 | 69.00 | 74.00 | 76.00 | 83.00 | 85.00 | 89.00 |
| Subject26 | 0.00 | 65.50 | 97.00 | 98.50 | 99.50 | 99.00 | 100.00 | 100.00 | 100.00 | 100.00 | 100.00 |
| Subject27 | 0.00 | 9.50 | 46.50 | 69.00 | 75.00 | 80.00 | 84.00 | 86.00 | 91.50 | 92.50 | 94.50 |
| Subject28 | 0.00 | 10.50 | 25.50 | 46.00 | 61.50 | 74.50 | 82.50 | 89.00 | 89.00 | 91.50 | 92.00 |
| Subject29 | 0.00 | 7.50 | 15.50 | 33.50 | 38.00 | 36.00 | 42.50 | 42.50 | 39.00 | 42.00 | 42.50 |
| Subject30 | 0.00 | 9.50 | 14.00 | 25.00 | 27.50 | 31.50 | 35.00 | 39.50 | 43.00 | 46.50 | 50.00 |
| Subject31 | 0.00 | 30.00 | 61.00 | 69.50 | 73.00 | 78.00 | 80.50 | 82.00 | 82.00 | 81.50 | 81.00 |
| Subject32 | 0.00 | 11.50 | 25.00 | 52.00 | 70.50 | 77.00 | 77.50 | 80.00 | 79.50 | 80.00 | 80.50 |
| Subject33 | 0.00 | 16.00 | 38.00 | 53.00 | 65.00 | 73.00 | 75.50 | 78.50 | 83.50 | 82.50 | 85.50 |
| Subject34 | 0.00 | 7.00 | 11.00 | 21.00 | 42.50 | 46.50 | 52.00 | 52.00 | 51.00 | 55.50 | 55.00 |
| Subject35 | 0.00 | 19.50 | 55.50 | 73.00 | 78.00 | 89.00 | 91.00 | 95.00 | 97.00 | 97.00 | 97.00 |
| Average  ±Standard Deviation | 0.00  ±0 | 21.36  ±18.10 | 44.59  ±26.72 | 55.80  ±26.60 | 63.23  ±24.87 | 68.23  ±24.15 | 71.93  ±23.13 | 74.19  ±22.69 | 75.79  ±22.47 | 77.43  ±21.85 | 78.54  ±21.57 |
