# Supplementary material for: Dual-Alpha: a large EEG study for dual-frequency SSVEP brain–computer interface
Source: Gigascience. 2024 Aug 7;13:giae041. doi: 10.1093/gigascience/giae041 (PMC11304967; doi:10.1093/gigascience/giae041)
Supplement: giae041_Response_to_Reviewer_Comments_Original_Submission [file giae041_response_to_reviewer_comments_original_submission.pdf]

Author's Response To Reviewer Comments

Response to Revision Request
Dear Dr. Nogoy,
Thank you for your email and the constructive feedback provided by the reviewers regarding our manuscript titled "Dual-Alpha: A Large EEG Study for Dual-Frequency SSVEP Brain-Computer Interface" (GIGA-D-24-00125). We are grateful for the opportunity to revise our manuscript and address the concerns raised to enhance its quality and suitability for publication in GigaScience.
We have carefully considered each comment and have undertaken the necessary revisions as suggested. As advised, we have critically reviewed our reference list and reduced the number of self-citations. We now only include five of our previous works that are directly relevant to the current study. This adjustment ensures our references are comprehensive and unbiased, adhering closely to the journal's guidelines. And the RRID identifiers have been included in the revised manuscript.
In accordance with the guidelines outlined in your letter, we have uploaded the revised manuscript file. Additionally, we have included a copy of the original manuscript with all the modifications highlighted in purple for your convenience. Alongside this letter, we have attached our detailed point-by-point response to the reviewers' comments. The comments have been reproduced, and our corresponding responses are provided in a distinct color (purple).
We appreciate the detailed guidance from the reviewers and the editorial team throughout this process. Please do not hesitate to contact us if further information or additional revisions are required.
Thank you for considering our work for publication. We eagerly await your response.
Warm regards,
Professor, Xiaorong Gao, Tsinghua University
Reviewer's Comments:
Reviewer: 1

This study presented a large-scale SSVEP dataset, encompassing over one hundred participants. The acquisition and validation of SSVEP data across CA, BV, and BsV paradigms were detailed introduced. This dataset has the potential to drive the development of SSVEP technology. The following comments are provided to help improve the manuscript before publication.
R: Thanks very much for your constructive comments and for recognizing the potential impact of our large-scale SSVEP dataset. We appreciate the time you have taken to review our manuscript and are grateful for your suggestions, which we believe will significantly enhance the quality and clarity of our paper.
1. Page 1: "In response, a study in 2022 introduced a dual-frequency SSVEP paradigm using 3D display technology, leveraging polarized light to effectively separate the dual frequencies and reduce UIHC generation [13]"
We did not find any relevant introduction or literature annotation regarding the abbreviation "BV" in the "Context" section.
A: Apologies for the oversight in our manuscript. The abbreviation "BV" stands for Binocular Vision. We have now added a clarification to the sentence you referenced. We deeply regret any confusion this may have caused and appreciate your attention to detail.
2. The authors should thoroughly compare and explain the differences between "BV" and "BsV" in the "Stimulus interface and encoding" section.
A: Thank you very much for your suggestion. We have added detailed explanations clarifying the differences between BV and BsV in multiple sections of our manuscript, including "Context," "Participant Information and Experimental Setup," and the "Stimulus Interface and Encoding" section you mentioned. Your feedback has been invaluable and has significantly enhanced the quality of our paper.
3. Page 5: "Panel I. illustrates the frequency-phase encoding scheme used for the CA and BV paradigms …. Panel II. displays the encoding scheme for the BV and BsV paradigms.
Q: Which panel illustrates the BV paradigm, Panel I or Panel II?
A: We apologize for the confusion caused by not clearly differentiating between the BV and BsV paradigms earlier in the manuscript. In fact, the main differences between these two paradigms lie in their encoding and decoding algorithms and the features they focus on, though they appear similar externally during data collection. To clarify, Panel II serves as a shared illustration for both the BV and BsV paradigms. We have amended the text to better explain this and hope it resolves any confusion. Thank you for bringing this to

our attention.
4. The parameters of the classification model need to be described in detail in the "SSVEP classification algorithm" section.
A: Thank you for your suggestion. We have now provided a more detailed description of the algorithm model and its parameters in the relevant section. We hope this addresses your concerns and clarifies any uncertainties.
5. We recommend the authors to include an analysis of the impact of the number of channels on classification accuracy in the "Data Validation and quality control" section.
A: Thank you very much for your suggestion. We have added a subsection at the end of the "Data Validation and Quality Control" section to analyze the relationship between the number of channels and classification accuracy. Your feedback is crucial to enhancing the quality of our paper.

Reviewer: 2

The authors presented a large dual-SSVEP dataset consisting of over 100 participants performing three different stimulation paradigms on a 40-target SSVEP speller interface. This dataset is valuable in terms of its size and novel dual-frequency SSVEP paradigms, which can be used as a binocular dual-SSVEP benchmark.
R: We greatly appreciate your positive feedback on our dataset and your recognition of its contributions to the field of SSVEP research. We are grateful for your supportive and insightful feedback, which encourages us to continue our work with even greater rigor and enthusiasm. We look forward to possibly incorporating any additional suggestions you might have to further enhance the manuscript.
Nonetheless, addressing the following questions can further help readers better understand the dataset.
1. Is there any bad trials detection and rejection step performed? Do all subjects have the same amount of data?
A: Thank you for your inquiry. We maintain strict quality control during data collection in our experiments. Each trial is assessed in real-time, and any problematic trials are immediately recollected. Therefore, the data used in our article should be free from bad trials. Regarding your second question, yes, each subject has an equal amount of data. We have added clarifications in the corresponding sections of our paper to emphasize these points. We hope this addresses your concerns.
2. Figure 4 shows the PSD, which does not seem to have any alpha band noise. Since the alpha rhythm should be common during SSVEP experiments, is baseline correction performed to eliminate the alpha power? How would alpha wave influence the CCA analysis if the stimulating frequency falls in the alpha band range?
A: Thank you very much for your comments. Unfortunately, there may have been some confusion in our description, and I apologize for any uncertainty this may have caused. The graph shown in Figure 4 is not a result of the Power Spectral Density (PSD) analysis, but rather a spectral plot from the Canonical Correlation Analysis (CCA), which represents the distribution of correlation values across frequencies computed by iterating the CCA for each frequency. We opted for this method because our encoding precision is up to 0.1 Hz, and theoretically, a clear PSD would require ten seconds of data, which we do not have. The CCA spectral method enhances frequency resolution, thus providing clearer results.
Regarding your second question, since CCA seeks to identify a linear vector that weights the multi-channel signals to maximize correlation with sine and cosine templates, the influence of large-scale EEG activities, such as alpha waves, is minimal. In each channel, the signals are very similar, so the weighting in this context has a negligible effect, akin to an overall proportional scaling up or down. Consequently, prominent bands like the alpha wave are unlikely to appear distinctly in the CCA spectrum.
Research using the CCA spectrum for analysis is quite sparse, which can indeed lead to confusion. We are currently preparing another paper to clearly explain how this method improves frequency resolution and we hope to extend its application to other signal analysis tasks in various fields.
We have also added an explanation in the relevant section of our article to address this concern. We deeply appreciate your feedback, as it is invaluable in enhancing the quality of our work.
3. Authors performed detailed classification between 40 targets. How about with an NA target (no stimulation at all), since SSVEP detection is also vital for an asynchronous SSVEP system, it would be interesting to study the detection accuracy as well.
A: Thank you very much for your suggestion. We also believe that asynchronous systems are an important direction in this research field. Currently, there is indeed a lack of relevant datasets. We have added a discussion section in the paper to highlight and remind readers of this issue, acknowledging it as one of the

current limitations of our dataset. Collecting data for an asynchronous system is one of our future research objectives.
Reviewer 3:

This article describes a dual-frequency SSVEP dataset stimulated using checkerboard arrangement, binocular vision, and binocular-swap vision paradigms. 35 participants were recruited for each paradigm. Although the participant groups are different in each paradigm, I agree that this dataset is a valuable asset to the community as, to my knowledge, there is yet a comprehensive binocular vision dual-frequency SSVEP dataset.
R: Thank you for recognizing the value of our dataset and its contribution to the field of SSVEP research. We are glad that the novelty of the dual-frequency SSVEP dataset stimulated through checkerboard arrangement, binocular vision, and binocular-swap vision paradigms is acknowledged as a significant asset to the community. We believe that these clarifications and future research directions will strengthen the manuscript and provide a more comprehensive understanding of the dataset's implications.
The article is well written in general, however, some further details and edits are needed.
General:
1. The article is missing a Usage Notes section which helps readers to understand how to use this dataset.
A: We agree that a "Usage Notes" section is essential for guiding users on how to effectively utilize the dataset. To this end, we will add a detailed "Usage Notes" section to our manuscript. This section will include information on how to access the dataset, an explanation of the data structure, potential use cases, and recommendations for data handling to assist users in navigating and exploiting the dataset effectively for their research purposes.
2. More details should be presented regarding the data structure. This could be included in the Usage Notes section or README file in your repository, or both.
A: In response to your suggestion, we will provide a comprehensive description of the data structure. This will be included in both the "Usage Notes" section of the manuscript and as a detailed README file in the dataset repository. This documentation will ensure that users can easily understand and manipulate the data according to their research needs.
3. It would be very helpful if you can list the participants who have completed more than 1 paradigm in this study. And list their subject numbers in each paradigm.
A: Thank you for your suggestion. We have added a table in the supplementary materials and dataset to clearly indicate which participants are the same across different paradigms.
Context:
1. The last paragraph is not very well justified. It is not clear why 40-target set up matters. Please consider reorganising this paragraph to highlight why this dataset is valuable and how it is different to the others.
A: Thank you for your feedback. We have completely rewritten the last paragraph to better emphasize the significance of the 40-target dataset and to clarify its unique value compared to other datasets.
Methods:
1. Participant information: please comment on if they have any conditions or impairments with their eyes or any neurological conditions?
A: Thank you very much for your query. None of the participants recruited for our study have any ophthalmic or neurological conditions. We have added this information to the manuscript to clarify.
2. Explain how the participants were recruited and how they were assigned to different groups (paradigms).
A: Thank you for your suggestion. Our dataset was compiled through collaborations across multiple teams, with each team responsible for data collection under a specific paradigm. Hence, participant recruitment for the three paradigms was conducted separately, and there was no process of assigning participants to different groups by us. Any overlap in participants occurred because individuals, who had a keen interest in BCI, voluntarily signed up for multiple experiments. We have not actively assigned participants to different groups. We have now added this information to the manuscript to clarify this process.
3. Table 1: explain everything in the table. It is not clear what you are presenting following the mean age. Is it standard deviation, standard error, or something else?
A: Thank you very much for your helpful comment. The values presented following the mean age are indeed standard deviations. We have now added this clarification to the appropriate place in Table 1 to ensure this is clear.
4. First line below Table 1: avoid using subjective words and expressions like "the experimental procedure was rigorously designed".

A: We appreciate your suggestion and have revised the statement to remove subjective language. The revised sentence now objectively describes the experimental procedure.
5. Did any of your participants wore glasses? If yes, explain how the polarised glasses work with their own prescription glasses.
A: Thank you very much for your feedback. We had two types of polarized glasses: clip-on and frame-style. For participants who wore glasses, we provided clip-on polarized glasses. For those who did not wear glasses, we provided frame-style polarized glasses. Relevant content has also been added to the text.
6. Stimulation systems: need further details on how the targets were laid out on the screen.
A: We have added additional details regarding the layout of the targets on the screen in the Stimulation Systems section of the manuscript, ensuring that the setup is clearly described for reproducibility.
7. From my own experience, the Psychophysics Toolbox may have lagging issues sometimes and may influence the accuracy of frequency delivery. Did you experience this issue? If yes, any corrections performed?
A: Without seeing your specific code and setup, it's challenging to pinpoint the exact problem. In our experiments, we use photodiodes and other sensors to verify the accuracy of the stimuli before proceeding. So far, I haven't encountered similar issues. From my experience, you might consider preloading the data into the GPU memory and evaluating whether the transmission protocol between the computer and the monitor (we typically use DisplayPort) can support the image refresh rate. Lastly, it's crucial to test and validate the stimulus screen. In my experience, even monitors of the same model can have significant differences in speed of refresh.
8. Include screen refresh rate information in the same place where you introduced the model of the screen and its resolution.
A: We have included the screen refresh rate information alongside the model and resolution of the screen as per your suggestion. This should provide a more comprehensive specification of the display setup.
9. Equation 1 needs more explanation and corrections:
1) What type of rounding was used when converting float to integers?
A: We used floor rounding, and this detail has been added to the text.
2) Should the scaling factor for the cosine function be 0.5 instead of 0.25 so that it can use the full brightness range (0-255)?
A: Apologies, this was a mistake in our writing process. The factor should indeed be 0.5. Thank you very much for pointing that out.
3) 'r' was not explained.
A: Apologies for the oversight. 'r' stands for the refresh rate, and we have now included this explanation in the text.
4) The definition of sigma has problems. From your definition "sigma varies from 1 to 60 multiplied by the stimulation duration", it ranges from 1 to 120 which changes the scale.
A: Thank you for pointing this out. Sigma should indeed be in the numerator. This was a writing error on our part, and we apologize for the confusion.
10. Please provide more details explanation on the differences between BV and BsV.
A: Thank you for your suggestion. We have incorporated detailed explanations clarifying the differences between BV and BsV in several sections of our manuscript, including "Context," "Participant Information and Experimental Setup," and "Stimulus Interface and Encoding". Your feedback has been invaluable and has significantly improved the quality of our paper.
11. The last sentence on light intensity above Figure 2 is unclear. Please further explain.
A: Thank you very much for your suggestion. We have rewritten this sentence to ensure clearer expression.
12. Why not have CA cells be 1*1 pixel as well?
A: From the perspective of writing the paper, the reason for using three pixels is that previous studies have adopted this approach. However, on a personal note, I can tell you that we have tried single-pixel stimulation, but the results were not as effective as the three-pixel system. Since we haven't conducted a systematic scientific comparison, I cannot provide a definitive answer. However, I suspect that a single pixel may not be spatially distinguishable to the human eye, thus the stimulus sequence might be perceived as an envelope signal rather than a combination of two frequencies.
13. How were the frequencies and phases selected? How were the stimuli frequency and phase layouts determined?
A: Thank you for your question. We directly used the frequency-phase encoding and spatial arrangements from previous research. We have emphasized this point and added the relevant references in the "Stimulus

interface and encoding" section.
14. In Figure 3 caption, remove BV on the third line.
A: Thank you very much for your reminder. We have corrected this error in the text.
15. In SNR definitions, delta f definition is not clear.
A: Thank you very much for your reminder. The unclear definition was actually due to a mistake in the formula 2. We have corrected this error and hope this resolves your concern.
16. At the bottom of page 6/15, you mentioned that the data length is too short for plotting the spectra, a potential solution is to zero-pad your data to make it longer for better frequency resolution.
A: Thank you very much for your suggestion. Actually, zero-padding acts as a form of smooth interpolation for the spectrum and does not provide new information. The resolution achieved through zero-padding is only of computational significance, not of actual physical significance. The true improvement in spectral resolution can only be achieved by increasing the effective sample length. For a detailed explanation on this, you can refer to Steven W. Smith's "Digital Signal Processing: A Practical Guide for Engineers and Scientists."
17. The CCA spectra should be further explained with more details.
A: Thank you very much for your suggestion. We have added more details about the CCA spectra. However, it should be noted that this method is currently not widely used, and its underlying principles have not been fully analyzed. Since this is not the main focus of our paper, we have only provided a brief introduction to the method. We are in the process of writing another paper that will systematically summarize and analyze this method.
18. 'x(t)' in equation 3 was not explained.
A: Thank you very much for pointing that out. We have added the relevant information at the corresponding position.
Data validation and quality control:
1. On Figures 4 and 5, suggest labelling the linear combinations and harmonics on the graphs. E.g., 19.6Hz = f1+f2, as how you did with 11.6 Hz.
A: Thank you very much for your suggestion. We have modified the figures accordingly to label the linear combinations and harmonics as you recommended.
2. Figure 4: it would be great if you have participants who did both paradigms and if you can show their data for a more direct comparison.
A: Thank you for your suggestion. Unfortunately, after careful review, we found that there were no participants who completed both the CA and BV paradigms.
3. Figure 4: could you please explain why in the BV paradigm topography plots, why all of them showed strong activity on only one side?
A: This is indeed an interesting observation. I personally believe this is due to a stronger response in the right visual field compared to the left visual field. The underlying cause could be related to habitual eye use or individual differences in neural development. However, this is quite normal. In my personal experience, many people exhibit a lateralized SSVEP response area, and those with responses centered precisely at Oz are actually in the minority.
4. Figure 4 caption has a typo: 'CV' should be changed to 'CA'.
A: Thank you for pointing this out. We have corrected this error.
5. Figure 5: please consider having the topography of the same frequency vertically aligned and have the two waveforms plotted on the same figure to help visualise a direct comparison.
A: Thank you very much for your suggestion. However, I personally believe that this method might not be very intuitive. The overlap between the two waveforms is quite high, and if plotted together, readers might find it difficult to distinguish between them. I have attached an example figure for your reference to illustrate the effect in the word file.
Additionally, if we follow your previous suggestion to label the chart as "f1+f2" and overlay the two graphs, the different f1 and f2 for the two targets might confuse readers. For example, regarding 10.9Hz, one graph would show f1 and the other f2. Should I label it as f1 or f2? Or should I annotate it as f1 for Target A and f2 for Target B in the figure? Either way, I feel it would make the readers more confused. I believe the current arrangement is clearer. However, I still greatly appreciate your feedback as it is very important for improving the quality of our paper.
6. Third line in SNR ratio distribution analysis: the normal distribution does not support your claim "attesting to the robustness and reliability of the dataset". To show this, you will need to compare to other studies. For example reference 20 if you like.
A: Thank you very much for your comments. We realize that our initial statement might be confusing. We

have now added a comparison with previous seminal datasets to substantiate our point.
7. Average SNR: (second sentence) in narrowband SNR, BV>BsV according to your plot. Suggest running statistical tests on these comparisons. It looks like there might not be any significant differences between the different paradigms.
A: Thank you very much for your suggestion. We have incorporated the results of the statistical tests into Figure 7. Although the mean values appear to show only slight differences, the differences are actually statistically significant.
8. Figure 7: please update them to have the same scale. The differences are actually quite small, but with the zoomed in view, it may mislead reader to have a false perception.
A: Thank you very much for your suggestion. We apologize for any confusion caused by not presenting the statistical test results earlier. In fact, these differences are significant, and the zoomed-in view is necessary to clearly demonstrate these distinctions.
9. My rough thoughts on training-free classification on BsV: since differences were observed from the topography, perhaps applying different spatial filters may help in decoding BsV with a training-free method.
A: Your idea is quite interesting. However, the challenge lies in the fact that each individual's topographical map is different, with significant individual variability. It is challenging to identify a universal prior spatial filter that works for everyone. Nevertheless, I also believe that exploring methods to achieve training-free BsV classification is a valuable research direction.
10. Classification results with training: suggest also perform statistical tests to support your claims such as "The results demonstrate that the performance metrics of correctness and ITR for both the CA and BV paradigms are closely matched".
A: Thank you very much for your suggestion. We have now included statistical test results to support our claims.
Supplementary Tables:
1. Supplementary Table 1 can be combined with Table 1 in the paper.
A: We have combined Supplementary Table 1 with Table 1 in the main paper, as suggested. This consolidation should improve the clarity and accessibility of the data presented.
2. Supplementary Tables 2 and 3: make the units clear in your tables. Also show standard errors.
A: We have updated Supplementary Tables 2 and 3 to ensure that all units are clearly indicated. Additionally, we have included the standard errors for the measurements presented in these tables. These changes should enhance the comprehensibility and accuracy of the data.
