# Supplementary material for: Dual-Alpha: a large EEG study for dual-frequency SSVEP brain–computer interface
Source: Gigascience. 2024 Aug 7;13:giae041. doi: 10.1093/gigascience/giae041 (PMC11304967; doi:10.1093/gigascience/giae041)
Supplement: giae041_Reviewer_1_Report_Original_Submission — Yichuan Jiang -- 5/13/2024 Reviewed [file giae041_reviewer_1_report_original_submission.pdf]

Reviewer Report

Title: Dual-Alpha: A Large EEG Study for Dual-Frequency SSVEP Brain-Computer Interface

Version: Original Submission **Date:** 5/13/2024

Reviewer name: Yichuan Jiang

Reviewer Comments to Author:

This study presented a large-scale SSVEP dataset, encompassing over one hundred participants. The acquisition and validation of SSVEP data across CA, BV, and BsV paradigms were detailed introduced. This dataset has the potential to drive the development of SSVEP technology. The following comments are provided to help improve the manuscript before publication.Comment 1Page 1: "In response, a study in 2022 introduced a dual-frequency SSVEP paradigm using 3D display technology, leveraging polarized light to effectively separate the dual frequencies and reduce UIHC generation [13]"We did not find any relevant introduction or literature annotation regarding the abbreviation "BV" in the "Context" section.Comment 2Page 5:The authors should thoroughly compare and explain the differences between "BV" and "BsV" in the "Stimulus interface and encoding" section.Comment 3Page 5: "Panel I. illustrates the frequency-phase encoding scheme used for the CA and BV paradigms …. Panel II. displays the encoding scheme for the BV and BsV paradigms.Q: Which panel illustrates the BV paradigm, Panel I or Panel II?Comment 4The parameters of the classification model need to be described in detail in the "SSVEP classification algorithm" section.Comment 5We recommend the authors to include an analysis of the impact of the number of channels on classification accuracy in the "Data Validation and quality control" section.

Level of Interest

Please indicate how interesting you found the manuscript: Choose an item.

Quality of Written English

Please indicate the quality of language in the manuscript: Choose an item.

Declaration of Competing Interests

Please complete a declaration of competing interests, considering the following questions:

- Have you in the past five years received reimbursements, fees, funding, or salary from an organisation that may in any way gain or lose financially from the publication of this manuscript, either now or in the future?
- Do you hold any stocks or shares in an organisation that may in any way gain or lose financially from the publication of this manuscript, either now or in the future?

- Do you hold or are you currently applying for any patents relating to the content of the manuscript?
- Have you received reimbursements, fees, funding, or salary from an organization that holds or has applied for patents relating to the content of the manuscript?
- Do you have any other financial competing interests?
- Do you have any non-financial competing interests in relation to this paper?

If you can answer no to all of the above, write 'I declare that I have no competing interests' below. If your reply is yes to any, please give details below.

I declare that I have no competing interests

I agree to the open peer review policy of the journal. I understand that my name will be included on my report to the authors and, if the manuscript is accepted for publication, my named report including any attachments I upload will be posted on the website along with the authors' responses. I agree for my report to be made available under an Open Access Creative Commons CC-BY license (http://creativecommons.org/licenses/by/4.0/). I understand that any comments which I do not wish to be included in my named report can be included as confidential comments to the editors, which will not be published.

Choose an item.

To further support our reviewers, we have joined with Publons, where you can gain additional credit to further highlight your hard work (see: https://publons.com/journal/530/gigascience). On publication of this paper, your review will be automatically added to Publons, you can then choose whether or not to claim your Publons credit. I understand this statement.

Yes Choose an item.
