# Supplementary material for: Dual-Alpha: a large EEG study for dual-frequency SSVEP brain–computer interface
Source: Gigascience. 2024 Aug 7;13:giae041. doi: 10.1093/gigascience/giae041 (PMC11304967; doi:10.1093/gigascience/giae041)
Supplement: giae041_Reviewer_2_Report_Original_Submission — Marc Van Hulle -- 5/15/2024 Reviewed [file giae041_reviewer_2_report_original_submission.pdf]

Reviewer Report

Title: Dual-Alpha: A Large EEG Study for Dual-Frequency SSVEP Brain-Computer Interface

Version: Original Submission **Date:** 5/15/2024

Reviewer name: Marc Van Hulle

Reviewer Comments to Author:

The authors presented a large dual-SSVEP dataset consisting of over 100 participants performing three different stimulation paradigms on a 40-target SSVEP speller interface. This dataset is valuable in terms of its size and novel dual-frequency SSVEP paradigms, which can be used as a binocular dual-SSVEP benchmark.Nonetheless, addressing the following questions can further help readers better understand the dataset.1. Is there any bad trials detection and rejection step performed? Do all subjects have the same amount of data?2. Figure 4 shows the PSD, which does not seem to have any alpha band noise. Since the alpha rhythm should be common during SSVEP experiments, is baseline correction performed to eliminate the alpha power? How would alpha wave influence the CCA analysis if the stimulating frequency falls in the alpha band range?3. Authors performed detailed classification between 40 targets. How about with an NA target (no stimulation at all), since SSVEP detection is also vital for an asynchronous SSVEP system, it would be interesting to study the detection accuracy as well.

Level of Interest

Please indicate how interesting you found the manuscript: Choose an item.

Quality of Written English

Please indicate the quality of language in the manuscript: Choose an item.

Declaration of Competing Interests

Please complete a declaration of competing interests, considering the following questions:

- Have you in the past five years received reimbursements, fees, funding, or salary from an organisation that may in any way gain or lose financially from the publication of this manuscript, either now or in the future?
- Do you hold any stocks or shares in an organisation that may in any way gain or lose financially from the publication of this manuscript, either now or in the future?
- Do you hold or are you currently applying for any patents relating to the content of the manuscript?
- Have you received reimbursements, fees, funding, or salary from an organization that holds or has applied for patents relating to the content of the manuscript?
- Do you have any other financial competing interests?

- Do you have any non-financial competing interests in relation to this paper?

If you can answer no to all of the above, write 'I declare that I have no competing interests' below. If your reply is yes to any, please give details below.

No

I agree to the open peer review policy of the journal. I understand that my name will be included on my report to the authors and, if the manuscript is accepted for publication, my named report including any attachments I upload will be posted on the website along with the authors' responses. I agree for my report to be made available under an Open Access Creative Commons CC-BY license (http://creativecommons.org/licenses/by/4.0/). I understand that any comments which I do not wish to be included in my named report can be included as confidential comments to the editors, which will not be published.

Choose an item.

To further support our reviewers, we have joined with Publons, where you can gain additional credit to further highlight your hard work (see: https://publons.com/journal/530/gigascience). On publication of this paper, your review will be automatically added to Publons, you can then choose whether or not to claim your Publons credit. I understand this statement.

Yes Choose an item.
