# Supplementary material for: Dual-Alpha: a large EEG study for dual-frequency SSVEP brain–computer interface
Source: Gigascience. 2024 Aug 7;13:giae041. doi: 10.1093/gigascience/giae041 (PMC11304967; doi:10.1093/gigascience/giae041)
Supplement: giae041_Reviewer_3_Report_Original_Submission — Jing Mu -- 5/16/2024 Reviewed [file giae041_reviewer_3_report_original_submission.pdf]

Reviewer Report

Title: Dual-Alpha: A Large EEG Study for Dual-Frequency SSVEP Brain-Computer Interface

Version: Original Submission **Date:** 5/16/2024

Reviewer name: Jing Mu

Reviewer Comments to Author:

This article describes a dual-frequency SSVEP dataset stimulated using checkerboard arrangement, binocular vision, and binocular-swap vision paradigms. 35 participants were recruited for each paradigm. Although the participant groups are different in each paradigm, I agree that this dataset is a valuable asset to the community as, to my knowledge, there is yet a comprehensive binocular vision dual-frequency SSVEP dataset.The article is well written in general, however, some further details and edits are needed.General:1. The article is missing a Usage Notes section which helps readers to understand how to use this dataset.2. More details should be presented regarding the data structure. This could be included in the Usage Notes section or README file in your repository, or both.3. It would be very helpful if you can list the participants who have completed more than 1 paradigm in this study. And list their subject numbers in each paradigm.Context:1. The last paragraph is not very well justified. It is not clear why 40-target set up matters. Please consider reorganising this paragraph to highlight why this dataset is valuable and how it is different to the others.Methods:1. Participant information: please comment on if they have any conditions or impairments with their eyes or any neurological conditions?2. Explain how the participants were recruited and how they were assigned to different groups (paradigms).3. Table 1: explain everything in the table. It is not clear what you are presenting following the mean age. Is it standard deviation, standard error, or something else?4. First line below Table 1: avoid using subjective words and expressions like "the experimental procedure was rigorously designed".5. Did any of your participants wore glasses? If yes, explain how the polarised glasses work with their own prescription glasses.6. Stimulation systems: need further details on how the targets were laid out on the screen.7. From my own experience, the Psychophysics Toolbox may have lagging issues sometimes and may influence the accuracy of frequency delivery. Did you experience this issue? If yes, any corrections performed?8. Include screen refresh rate information in the same place where you introduced the model of the screen and its resolution.9. Equation 1 needs more explanation and corrections:1) What type of rounding was used when converting float to integers?2) Should the scaling factor for the cosine function be 0.5 instead of 0.25 so that it can use the full brightness range (0-255)?3) 'r' was not explained.4) The definition of sigma has problems. From your definition "sigma varies from 1 to 60 multiplied by the stimulation duration", it ranges from 1 to 120 which changes the scale.10. Please provide more details explanation on the differences between BV and BsV.11. The last sentence on light intensity above Figure 2 is unclear. Please further explain.12. Why not have CA cells be 1*1 pixel as well?13. How were the frequencies and phases selected? How were the stimuli frequency and phase layouts determined?14. In Figure 3 caption, remove BV on the third line.15. In SNR definitions, delta f definition is not clear.16. At the bottom of page 6/15, you mentioned that the data length is too short for plotting the spectra, a potential solution is to zero-pad your data to make it longer for better

frequency resolution.17. The CCA spectra should be further explained with more details.18. 'x(t)' in equation 3 was not explained.Data validation and quality control:1. On Figures 4 and 5, suggest labelling the linear combinations and harmonics on the graphs. E.g., 19.6Hz = f1+f2, as how you did with 11.6 Hz.2. Figure 4: it would be great if you have participants who did both paradigms and if you can show their data for a more direct comparison.3. Figure 4: could you please explain why in the BV paradigm topography plots, why all of them showed strong activity on only one side?4. Figure 4 caption has a typo: 'CV' should be changed to 'CA'.5. Figure 5: please consider having the topography of the same frequency vertically aligned and have the two waveforms plotted on the same figure to help visualise a direct comparison.6. Third line in SNR ratio distribution analysis: the normal distribution does not support your claim "attesting to the robustness and reliability of the dataset". To show this, you will need to compare to other studies. For example reference 20 if you like.7. Average SNR: (second sentence) in narrowband SNR, BV>BsV according to your plot. Suggest running statistical tests on these comparisons. It looks like there might not be any significant differences between the different paradigms.8. Figure 7: please update them to have the same scale. The differences are actually quite small, but with the zoomed in view, it may mislead reader to have a false perception.9. My rough thoughts on training-free classification on BsV: since differences were observed from the topography, perhaps applying different spatial filters may help in decoding BsV with a training-free method.10. Classification results with training: suggest also perform statistical tests to support your claims such as "The results demonstrate that the performance metrics of correctness and ITR for both the CA and BV paradigms are closely matched".Supplementary Tables:1. Supplementary Table 1 can be combined with Table 1 in the paper.2. Supplementary Tables 2 and 3: make the units clear in your tables. Also show standard errors.

Level of Interest

Please indicate how interesting you found the manuscript: Choose an item.

Quality of Written English

Please indicate the quality of language in the manuscript: Choose an item.

Declaration of Competing Interests

Please complete a declaration of competing interests, considering the following questions:

- Have you in the past five years received reimbursements, fees, funding, or salary from an organisation that may in any way gain or lose financially from the publication of this manuscript, either now or in the future?
- Do you hold any stocks or shares in an organisation that may in any way gain or lose financially from the publication of this manuscript, either now or in the future?
- Do you hold or are you currently applying for any patents relating to the content of the manuscript?

- Have you received reimbursements, fees, funding, or salary from an organization that holds or has applied for patents relating to the content of the manuscript?
- Do you have any other financial competing interests?
- Do you have any non-financial competing interests in relation to this paper?

If you can answer no to all of the above, write 'I declare that I have no competing interests' below. If your reply is yes to any, please give details below.

I was in the same field in the past few year and now exploring other areas. I have a multi-frequency (including dual-frequency) SSVEP dataset published early this year closely related to this work. No other competing interest was identified.

I agree to the open peer review policy of the journal. I understand that my name will be included on my report to the authors and, if the manuscript is accepted for publication, my named report including any attachments I upload will be posted on the website along with the authors' responses. I agree for my report to be made available under an Open Access Creative Commons CC-BY license (http://creativecommons.org/licenses/by/4.0/). I understand that any comments which I do not wish to be included in my named report can be included as confidential comments to the editors, which will not be published.

Choose an item.

To further support our reviewers, we have joined with Publons, where you can gain additional credit to further highlight your hard work (see: https://publons.com/journal/530/gigascience). On publication of this paper, your review will be automatically added to Publons, you can then choose whether or not to claim your Publons credit. I understand this statement.

Yes Choose an item.
